# Hydroxyoleoside-type *seco*-iridoids from *Symplocos cochinchinensis* and their insulin mimetic activity

**DOI:** 10.1038/s41598-018-38013-4

**Published:** 2019-02-19

**Authors:** Ba-Wool Lee, Thi Kim Quy Ha, Ha Thanh Tung Pham, Quynh Hoa Hoang, Van On Tran, Won Keun Oh

**Affiliations:** 1 Korea Bioactive Natural Material Bank, Research Institute of Pharmaceutical Sciences, College of Pharmacy, Seoul National University, Seoul, 151-742 Republic of Korea; 2 Department of Botany, Hanoi University of Pharmacy, Hanoi, Vietnam

## Abstract

As part of an ongoing study of new insulin mimetic agents from medicinal plants, the 70% EtOH extract of *Symplocos cochinchinensis* was found to have a stimulatory effect on glucose uptake in 3T3-L1 adipocyte cells. The intensive targeted isolation of this active extract resulted in ten new hydroxyoleoside-type compounds conjugated with a phenolic acid and monoterpene (**1**–**6** and **8**–**11**), as well as four known compounds (**7** and **12**–**14**). The chemical structures of the new compounds were determined based on spectroscopic data analysis (^1^H and ^13^C NMR, HSQC, HMBC, NOESY and MS). The absolute configurations of the isolated compounds were determined by electronic circular dichroism (ECD) analysis of derivatives obtained after a series of reactions, such as those with dirhodium (ІІ) tetrakis (trifluoroacetate) and dimolybdenum (ІІ) tetraacetate. *In vitro*, compounds **3**, **7** and **8** moderately increased the 2-deoxy-2-[(7-nitro-2,1,3-benzoxadiazol-4-yl)amino]-D-glucose (2-NBDG) uptake level in differentiated 3T3-L1 adipocytes. For further studies, we evaluated their effects on the expression of glucose transporter-4 (GLUT4), its translocation, protein tyrosine phosphatase 1B (PTP1B) inhibition and expression of phosphorylated Akt. Our results strongly suggest that the traditional uses of this plant can be described as active constituents by hydroxyoleoside-type compounds.

## Introduction

Globally, the number of people with diabetes mellitus is growing rapidly, and the incidence rate of diabetes is also accelerating, especially as the elderly and obese population increases^1^. The number of patients with diabetes is expected to increase from 171 million in 2000 to 366 million globally by 2030. According to the American Diabetes Association, the incidence of diabetes is approximately 25.2% of the total elderly population in the United States and 12.0 million seniors suffered from diabetes in 2015. As the increase in diabetic patients is associated with a dramatic increase in the cost of diabetes-related complications, direct healthcare costs and productivity losses in the US alone are estimated to be $ 176 billion and $ 69 billion in 2012, respectively^2^.

Diabetes is a chronic disease that occurs when the pancreas does not produce enough insulin (type 1 diabetes) or the insulin produced does not function effectively at the site of action (type 2 diabetes, T2D). When insulin does not function properly, the increased blood sugar in the body can cause serious damage to the heart, blood vessels, kidneys, eyes and nerves. Since T2D associated with insulin resistance is the most prevalent^3^, it is urgently necessary to develop new anti-diabetic agents, especially those of targeting type 2 diabetes.

While many diabetes medications lower blood glucose levels in the short term, they often cause weight gain as a side effect and prolonged use worsens the insulin resistance of diabetic patients^3^. Insulin mimetics used as oral diabetic agents, which act similar to insulin but do not synthesize fats, have been suggested as a good solution for the treatment for diabetes. Interestingly, food intake and body weight decrease when insulin is selectively delivered to the brain, but not when it is delivered to the whole body^4^. These results suggest that insulin mimetics that separate glucose-lowering action from the weight gain are a very good pharmacological solution for overcoming insulin resistance as the side effects of diabetes therapies.

*Symplocos cochinchinensis* (Lour.) S. Moore (www.theplantlist.org) is an evergreen tree that grows up to 35 meters in height and belongs to the Symplocaceae family. This plant is distributed in East Asia, including China, Japan, India, Vietnam and Malaysia^5^. Ethnobotanical uses of this plant include treatment of diabetes mellitus in traditional “Ayurvedic” Indian medicine^6^. There are several reports on extracts from this plant showing antidiabetic^7,8^, antilipidemic and antioxidant activity^9^, but there have been few studies on the chemical constituents of *S*. *cochinchinensis*. The genus *Symplocos* contains a large amount of *seco*-iridoid and phenolic compounds^10^. Recent reports on the antidiabetic activity of oleuropein, which is abundant in olive tree leaves^11^, led us to isolate active compounds by a special dereplication method aimed at *seco*-iridoids.

**Figure 1:**
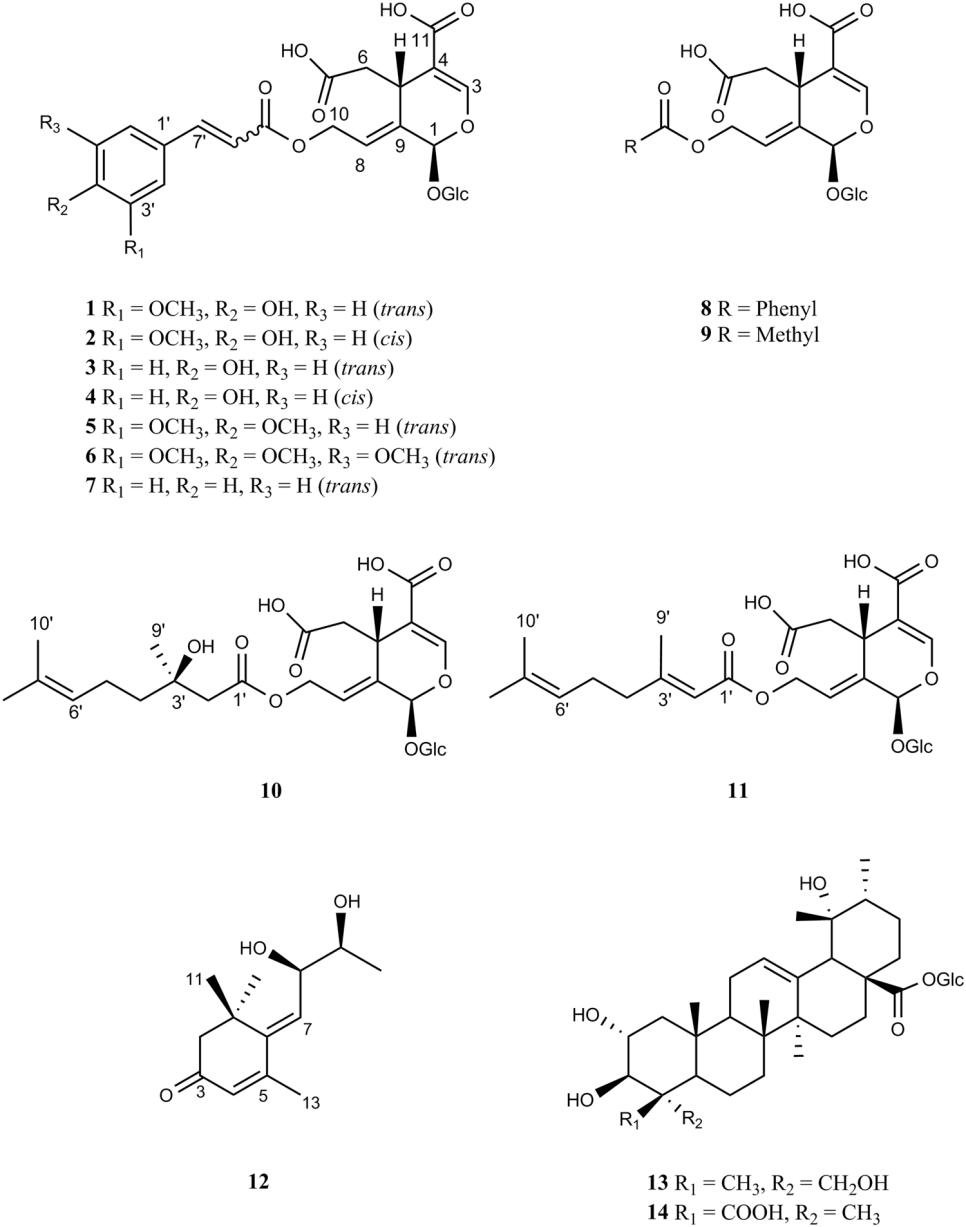
Chemical structures of compounds isolated from *S*. *cochinchinensis*.

In the search for new insulin mimetics from *S*. *cochinchinensis*, the 70% EtOH extract of the plant showed a moderate increase in glucose uptake in differentiated adipocyte cells. Bioassay-guided fractionation resulted in the isolation of ten new hydroxyoleoside-type compounds, including eight phenolic hydroxyoleosides, symplocochinside A-H (**1**–**6**, **8** and **9**), and two monoterpene-derivatized hydroxyoleosides, symplocochinside I-J (**10**–**11**), along with four known compounds, including a megastigmane and triterpene glycosides (Fig. 1). The absolute configurations of the monoterpene attached to hydroxyoleoside (**10**) and megastigmane (**12**) were assigned by chemical methods coupled with spectroscopic analysis. All isolates were evaluated for glucose uptake level, GLUT-4 translocation, PTP1B activity and Akt phosphorylation. In this paper, we report the isolation, structural elucidation, determination of absolute configuration and anti-diabetic properties of these isolates.

## Results and Discussion

### Structural elucidation of new compounds

The 70% EtOH extract of the aerial part of *S*. *cochinchinensis* was separated by silica gel, Sephadex LH-20, ODS open column chromatography and preparative HPLC to yield ten new 10-hydroxyoleoside-type *seco*-iridoids, symplocochinsides A-F (**1**–**6**) and G-J (**8**–**11**), together with four known compounds (Fig. 1).

**Figure 2:**
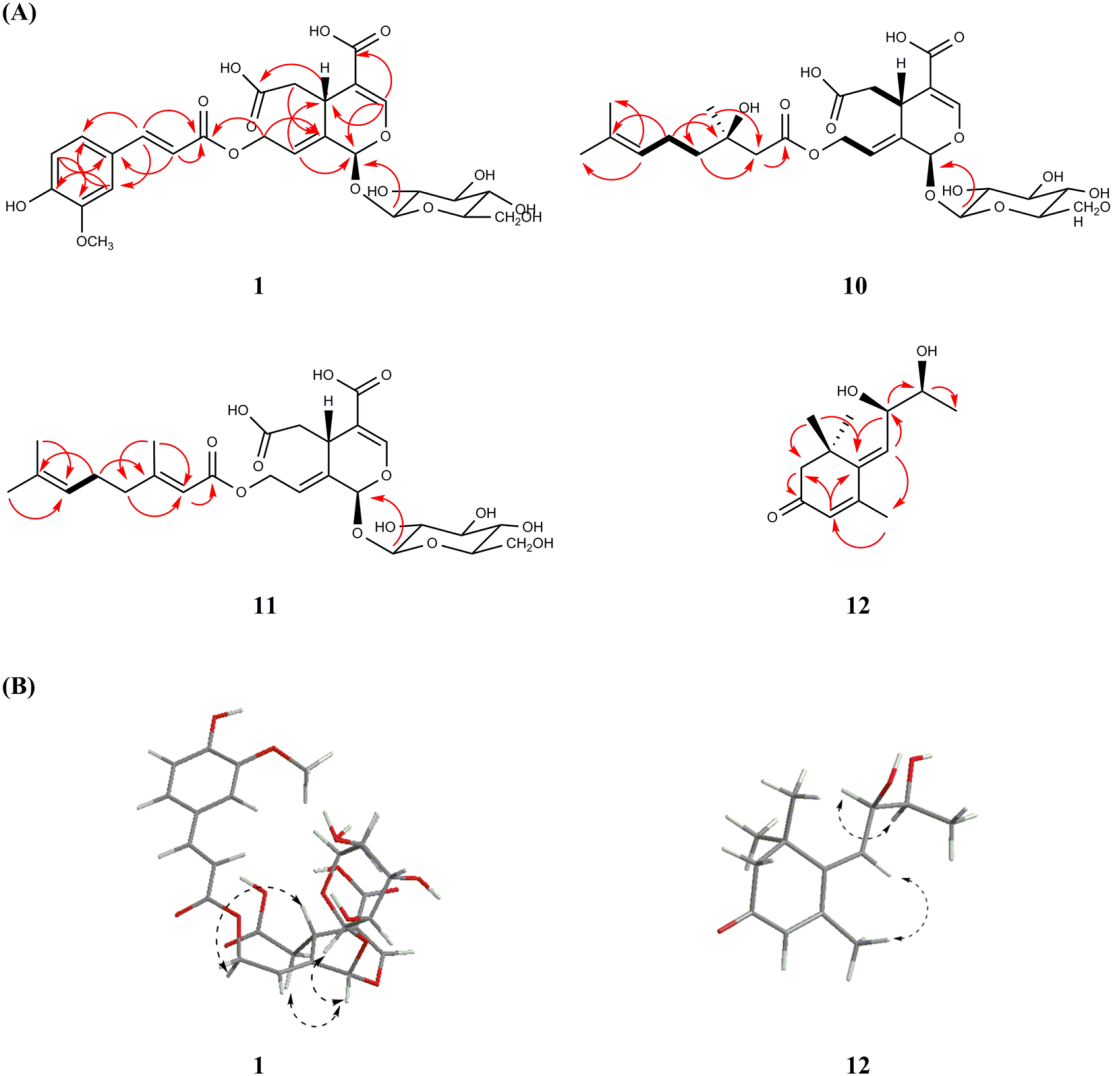
(**A**) Key HMBC and COSY correlations, (**B**) ROESY correlations for the compound **1** and **10**–**12**.

**Figure 3:**
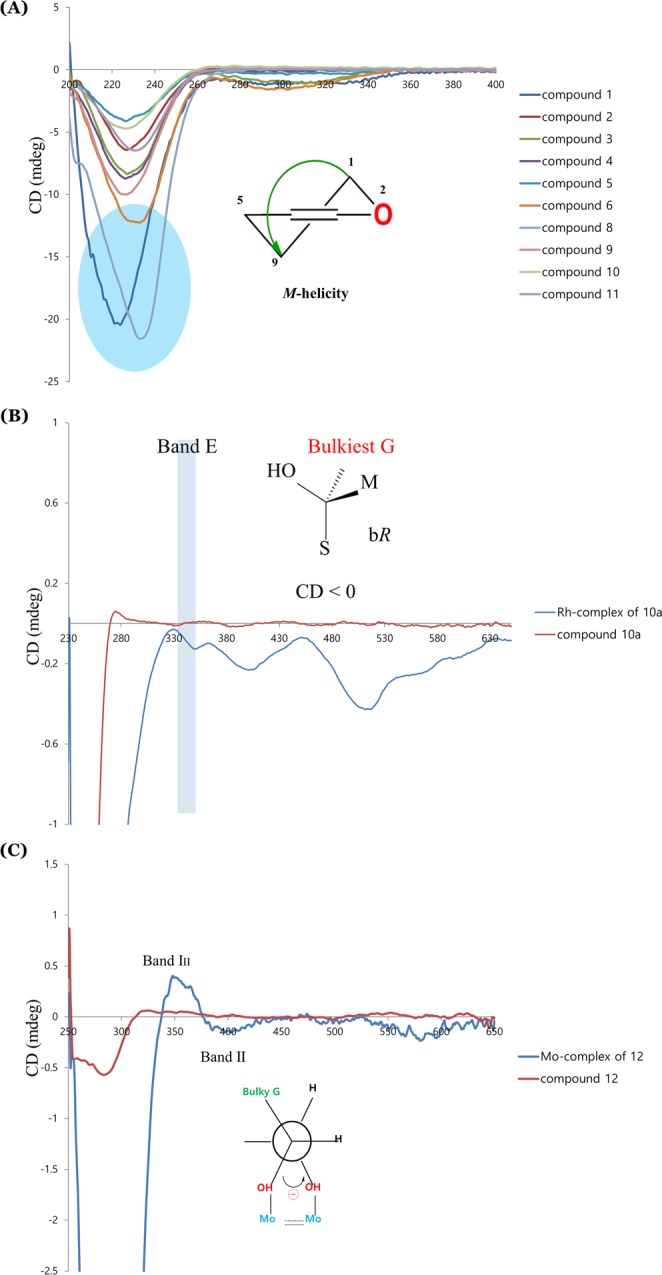
(**A**) Application of a helicity rule in experimental CD spectra (MeOH) for the absolute configuration of the dihydropyran moiety from all isolated *seco*-iridoids. (**B**) Empirical CD method using [Rh_2_(OCOCF_3_)_4_] in CDCl_3_ of compound **10a**. (**C**) Experimental CD method using [Mo_2_(OAc)_4_] in DMSO for compound **12**.

Symplocochinside A (**1)** was obtained as a brownish gum, and the molecular formula was established as C_26_H_30_O_15_ by HREIMS, which showed a ion peak at *m*/*z* 605.1478 [M + Na]^+^ (calcd for C_26_H_30_NaO_15_, 605.1477). The IR spectrum of **1** showed the presence of hydroxyl (3351 cm^−1^), carboxylic (1692 cm^−1^) and olefinic (1599 cm^−1^) functionalities. The ^1^H NMR spectrum (Table 1) of **1** displayed the typical signals of the 10-hydroxyoleoside skeleton, which is considered an important biosynthetic precursor as one of the major types of *seco*-iridoids, including one acetal proton of H-1 (*δ*_H_ 5.90, br s), two vinyl protons of H-3 (*δ*_H_ 7.46, br s), one methine group of H-5 (*δ*_H_ 3.88, br d, *J* = 9.6 Hz), one methylene group of H-6 [(*δ*_H_ 2.68, d, *J* = 14.4 Hz) and (*δ*_H_ 2.37, dd, *J* = 15.2, 10.4 Hz)], one olefinic proton of H-8 (*δ*_H_ 6.02, t, *J* = 6.4 Hz) and oxymethylene protons of H-10 [(*δ*_H_ 4.94, dd, *J* = 13.6, 8.8 Hz) and (*δ*_H_ 4.80, dd, *J* = 12.8, 4.8 Hz)]. The corresponding *seco*-iridoid carbon signals, which consisted of 26 carbon resonances (Table 2) from the ^13^C NMR spectrum of compound **1**, were observed at *δ*_C_ 92.0 (C-1), 152.3 (C-3), 109.0 (C-4), 31.1 (C-5), 40,0 (C-6), 122.3 (C-8) and 133.2 (C-9), as well as one oxygenated methylene carbon at 60.4 (C-10) and two carbonyl carbons at *δ*_C_ 172.8 (C-7) and 167.5 (C-11). Further analysis of the HMBC data of compound **1** (Fig. 2) confirmed the presence of a 10-hydroxyoleoside containing pyran ring moiety by the key correlations from H-8 (*δ*_H_ 6.02, t, *J* = 6.4 Hz) to C-1/C-5, from H-5 to C-1/C-3, from H-3 to C-1, and from H-10a/H-10b of the oxymethylene to C-8/C-9. In addition, HMBC correlations of H-5 with C-6/C-7/C-11 and of H-3 with C-11 indicated the positions of the carboxylic acids. The relatively large coupling constant (7.2 Hz) for the anomeric proton and six typical carbon resonances at *δ*_C_ 99.1 (C-1″), 73.3 (C-2″), 76.6 (C-3″), 70.0 (C-4″), 77.4 (C-5″) and 61.1 (C-6″) suggested the presence of a *β*-glucopyranosyl group. The splitting patterns of H-2′ (*δ*_H_ 7.32, d, *J* = 1.6 Hz), H-5′ (*δ*_H_ 6.79, d, *J* = 8.0 Hz) and H-6′ (*δ*_H_ 7.12, dd, *J* = 8.0, 0.8 Hz) in the lower field indicated the presence of a benzene ring with an ABX spin system. The key HMBC correlations from OMe (*δ*_H_ 3.80, s) to *δ*_C_ 148.0 (C-3′) and the carbon resonance at *δ*_C_ 149.4 (C-4′) are indicative of the presence of one methoxy and one hydroxy group, respectively. The large coupling constant between H-7′ (*δ*_H_ 7.55, d, *J* = 16.0 Hz) and H-8′ (*δ*_H_ 6.49, d, *J* = 16.0 Hz), the benzene ring system and the distinct UV absorption maximum at 324 nm strongly suggested the presence of a ferulic acid group with *α*,*β*-unsaturated ketone moiety. The HMBC correlation from H-10 to C-9′ at *δ*_C_ 166.5 proved that the ferulic acid moiety is linked to C-10 of hydroxyoleoside. The ROESY correlations of compound **1** (Fig. 2) suggested that the C-8 is in the *E* configuration and H-5 is in the *β* orientation because H-5 and H-10 become closer to each other, while the H-1/H-1″ and H-1/H-6 are on the same plane. Therefore, the relative configuration could be determined easily as [1S*, 5S*]. The electronic circular dichroism (ECD) spectrum of **1** with oleuropein^12^ displayed a negative Cotton effect (CE) at 226 nm (Fig. 3A) arising from the ^1^L_a_ band of the 4-carboxy-5,6-dihydro-[*1H*]-pyrano chromophore^13^. Since compound **1** assumes a distorted dihydropyran conformation^14^ and the helicity rule is applicable in this case, the CE with shorter wavelength predicts the 1*S* and 5*S* configuration^15,16^. In addition, chemical shifts of compounds with the 1*β* configuration generally appeared at approximately 94–96 ppm, while oleonin with the 1*α* configuration showed a chemical shift of 105.7 ppm in CD_3_OD^17^. Thus, compound **1** was determined as 10-*O*-*trans*-feruloyl-10-hydroxyoleoside with 1*S* and 5*S* (Fig. 1).

**Table 1:** ^1^H NMR data of compounds 1–6 and 8–11 (*δ* in ppm, *J* in Hz).

H	1^*a*^	2^*a*^	3^*a*^	4^*a*^	5^*b*^	6^*b*^	8^*b*^	9^*a*^	10^*a*^	11^*a*^
1	5.90, br s	5.86, s	5.90, br s	5.83, br s	5.99, br s	6.00, br s	6.02, br s	5.88, s	5.88, s	5.82, s
3	7.46, br s	7.42, br s	7.46, br s	7.38, br s	7.53, br s	7.54, br s	7.54, br s	7.47, s	7.46, s	7.37, s
5	3.88, br d (9.6)	3.86, br d (7.2)	3.88, d (9.6)	3.82, overlap	4.08, d (7.2)	4.09, dd (9.9, 2.4)	4.14, d (8.8)	3.82, dd (10.4, 3.2)	3.82, dd (10.6, 2.8)	3.79, d (10.4)
6	2.68, d (14.4)	2.66, d (13.9)	2.68, d (15.2)	2.67, d (15.2)	2.85, d (12.4)	2.86, dd (15.5, 2.0)	2.89, d (12.8)	2.66, dd (16.0, 3.2)	2.66, dd (16.1, 2.8)	2.63, d (13.4)
	2.37, dd (15.2, 10.4)	2.34, dd (15.2, 10.3)	2.37, dd (13.6, 10.4)	2.35, dd (13.6, 10.4)	2.43, dd (12.1, 12.1)	2.45, dd (14.2,11.4)	2.46, t (8.8)	2.36, dd (16.0, 10.4)	2.37, dd (15.7, 10.3)	2.25, d
8	6.02, t (6.4)	5.98, t (6.5)	6.02, t (6.4)	5.95, t (6.4)	6.12, t (6.4)	6.20, t (6.6)	6.26, t (6.4)	5.96, t (6.4)	5.96, t (6.5)	5.91, t (6.0)
10	4.94, dd (13.6, 8.8)	4.92, dd (13.4, 8.3)	4.94, dd (13.6, 8.8)	4.88, dd (13.6, 8.8)	5.04, dd (13.4, 7.9)	5.04, dd (13.5, 8.3)	5.18, dd (12.8, 8.0)	4.81, dd (13.6, 8.0)	4.81, dd (13.4, 8.3)	4.82, dd (13.8, 8.2)
	4.80, dd (12.8, 4.8)	4.73, dd (13.4, 4.8)	4.79, dd (12.8, 4.0)	4.72, dd (12.8, 4.0)	4.86, overlap	4.87, overlap	5.00, dd (12.8, 4.8)	4.65, m	4.66, m	4.67, dd (13.3, 4.6)
2′	7.32, d (1.6)	7.72, d (1.6)	7.55, d (8.8)	7.67, d (8.8)	7.20, d (1.6)	6.92, br s	8.02, d (7.2)		2.42, d (13.6)	5.68, d (0.8)
									2.38, d (13.7)	
3′			6.79, d (8.8)	6.76, d (8.0)			7.47, t (7.8)			
4′							7.60, t (7.5)		1.43, m	2.11–2.15, overlap
5′	6.79, d (8.0)	6.77, d (8.2)	6.79, d (8.8)	6.76, d (8.0)	6.97, d (8.4)		7.47, t (7.8)		1.97, m	2.11–2.15, overlap
6′	7.12, dd (8.0, 0.8)	7.17, dd (8.4, 1.9)	7.54, d (8.8)	7.65, d (8.8)	7.17, dd (8.3, 1.7)	6.92, s	8.02, d (7.2)		5.07, m	5.06, m
7′	7.55, d (16.0)	6.86, d (13.0)	7.57, d (15.9)	6.87, d (11.2)	7.63, d (15.9)	7.62, d (15.9)				
8′	6.49, d (16.0)	5.78, d (13.0)	6.40, d (16.0)	5.77, d (12.8)	6.39, d (15.9)	6.46, d (16.0)			1.56, br s	1.64, br s
9′									1.16, s	2.10, d (0.8)
10′									1.63, br s	1.57, s
1″	4.67, d (7.2)	4.65, d (7.6)	4.66, d (8.0)	4.65, d (8.0)	4.82, d (7.9)	4.82, d (7.8)	4.82,d (8.0)	4.66, d (8.0)	4.66, d (8.4)	4.65, d (8.1)
2″	3.09, m	3.08, m	3.08, m	3.09, dd (9.0, 8.4)	3.32, m	3.32, m	3.33, m	3.08, dd (8.1, 8.1)	3.08, m	3.07, m
3″	3.20, m	3.20, m	3.20, m	3.20, dd (9.2, 8.6)	3.40, dd (8.9, 8.9)	3.40, dd (8.8, 8.8)	3.41, dd (8.8, 8.8)	3.19, m	3.20, m	3.20, m
4″	3.08, m	3.08, m	3.08, m	3.07, dd (9.6, 9.0)	3.32, overlap	3.32, overlap	3.32, overlap	3.07, dd (8.1, 8.1)	3.08, m	3.08, m
5″	3.18, m	3.18, m	3.18, m	3.17, overlap	3.34, overlap	3.34, overlap	3.34, overlap	3.17, m	3.16, m	3.17, m
6″	3.68, br d (11.2)	3.67, br d (11.3)	3.68, br d (11.2)	3.68, br d (11.2)	3.88, dd (11.8, 1.7)	3.89, br d (12.8)	4.14, br d (8.8)	3.68, dd (12.0, 1.6)	3.67, dd (10.2, 3.0)	3.67, d (10.3)
	3.45, dd (11.2, 5.6)	3.45, dd (12.0, 6.3)	3.45, dd (12.0, 6.4)	3.45, dd (12.0, 6.4)	3.67, dd (12.1, 5.7)	3.67, dd (12.0, 5.8)	3.89, dd (11.2, 1.6)	3.45, dd (12.0, 6.4)	3.45, dd (12.0, 6.0)	3.44, dd (11.8, 6.5)
OAc								2.01, s		
OCH_3_-3′	3.80, s	3.76, s			3.86, s	3.86,s				
OCH4-4′					3.86, s	3.78, s				
OCH_3_-5′						3.86, s				

^*a*^Data were measured in DMSO-*d*_*6*_ at 800 MHz. ^*b*^Data were measured in CD_3_OD at 800 MHz.

**Table 2:** ^13^C NMR data of compounds 1–6 and 8–11 (*δ* in ppm).

C	1^*a*^	2^*a*^	3^*a*^	4^*a*^	5^*b*^	6^*b*^	8^*b*^	9^*a*^	10^*a*^	11^*a*^
1	92.0	92.0	92.0	92.0	94.3	94.3	94.2	92.0	92.1	91.9
3	152.3	151.8	152.5	151.3	154.6	154.7	154.6	152.7	152.4	151.5
4	109.0	109.6	108.7	110.3	109.2	109.9	110.1	108.4	108.6	106.7
5	31.1	31.1	31.0	31.2	32.9	32.7	32.9	30.9	30.9	31.2
6	40.0	40.1	39.7	41.2	41.4	41.1	41.4	40.0	40.1	40.4
7	172.8	172.8	172.6	173.0	†	175.0	175.3	171.8	172.5	172.9
8	122.3	121.7	122.3	121.4.	124.2	124.2	124.0	122.3	122.4	121.7
9	133.2	133.7	133.0	134.1	134.5	134.5	134.8	132.9	132.8	131.7
10	60.4	60.4	60.4	60.2	62.0	62.1	62.6	60.4	60.3	59.6
11	167.5	167.5	167.3	167.7	170.1	169.8	170.0	167.5	167.2	165.6
1′	125.6	125.6	125.1	125.3	128.8	131.6	131.4		170.6	167.6
2′	111.3	114.3	130.4	132.7	112.6	106.8	130.6		46.0	114.8
3′	148.0	146.9	115.8	115.0	150.8	154.8	129.6		70.1	160.5
4′	149.4	148.6	159.9	159.0	152.9	141.3	134.3		41.6	40.1
5′	115.6	115.0	115.8	115.0	112.6	154.8	129.6		22.2	25.47
6′	123.2	125.8	130.4	132.7	124.0	106.8	130.6		124.6	123.2
7′	145.3	144.2	145.0	143.8	146.6	146.5	167.8		130.5	131.7
8′	114.3	114.9	114.0	114.9	116.3	118.1			17.5	25.49
9′	166.5	165.8	166.4	165.7	168.7	168.4			27.0	18.5
10′									25.5	17.6
1″	99.1	99.1	99.0	99.1	101.0	101.0	100.9	99.1	99.1	99.0
2″	73.3	73.3	73.3	73.3	74.7	74.7	74.7	73.3	73.3	73.3
3″	76.6	76.5	76.5	76.5	77.9	77.9	77.9	76.6	76.6	76.5
4″	70.0	69.9	69.9	70.0	71.4	71.4	71.4	70.0	69.9	69.9
5″	77.4	77.5	77.4	77.4	78.5	78.5	78.5	77.4	77.4	77.4
6″	61.1	61.0	61.0	61.0	62.7	62.7	62.7	61.1	61.0	61.0
OAc								170.2		
								20.7		
OCH_3_-3′	55.7	55.5			56.51^*c*^	56.7				
OCH_3_-4′					56.41^*c*^	61.2				
OCH_3_-5′						56.7				

^*a*^Data were measured in DMSO-*d*_*6*_ at 200 MHz.

^*b*^Data were measured in CD_3_OD at 200 MHz.

^*c*^These values can be interchangeable.

^†^Peaks too small to be observed.

Symplocochinside B (**2)** was purified as a brownish gum, and its molecular formula was established as C_26_H_30_O_15_ from the HRESI mass data, which showed a ion peak at *m*/*z* 605.1476 [M + Na]^+^ (calcd for C_26_H_30_NaO_15_, 605.1477). The ^1^H NMR data of **2** (Table 1) were similar to those of **1** except for the configuration of the ferulic acid double bond. The *J* values of H-7′ (*δ*_H_ 6.86, d, *J* = 13.0 Hz) and H-8′ (*δ*_H_ 5.78, d, *J* = 13.0 Hz) of compound **1** are indicative of its *cis*-configuration. Whether the *cis* form of compound **2** is a plant-derived compound was determined by the retention time and abundance when the partial extract of *S*. *cochinchinensis* was co-injected with **2** on LC/MS. Hence, the structure of **2** was characterized as 10-*O*-*cis*-feruloyl-10-hydroxyoleoside.

Symplocochinside C (**3)** (Fig. 1) was isolated as a brownish gum, and its molecular formula was established as C_25_H_28_O_14_ by HRESI mass spectrum, which showed a ion peak at *m*/*z* 575.1371 [M + Na]^+^ (calcd for C_25_H_28_NaO_14_, 575.1371). The distinct UV patterns of **3**, which indicate the presence of a cinnamic acid moiety, and the characteristic proton peaks of H-1 (*δ*_H_ 5.90, s) and H-3 (*δ*_H_ 7.46, br s) showed the common features of this *seco*-iridoid. The ^1^H and ^13^C NMR spectra of **3** were similar to those of **1** except for the absence of the methoxy group. Since the *trans*-cinnamic acid derivative can be converted to the *cis*-isomer through photoisomerization^18^, the structure of compound **3** was determined after conversion to the *trans* form by the reaction with iodine^19^. Thus, the structure of **3** was determined as 10-*O*-*trans*-*p*-coumaroyl-10-hydroxyoleoside.

Symplocochinside D (**4)** was obtained as a brownish gum, and its molecular formula was established as C_25_H_28_O_14_ from the HRESI mass spectrum with a peak at *m*/*z* 575.1381 [M + Na]^+^ (calcd for C_25_H_28_NaO_14_, 575.1371). The 1D NMR of **4** (Tables 1 and 2) showed almost same patterns as those of **3** except for the coupling constants of H-7′ (*δ*_H_ 6.87, d, *J* = 11.2 Hz) and H-8′ (*δ*_H_ 5.77, d, *J* = 12.8 Hz), indicating that compound **4** is the *cis*-isomer of compound **3**. The pure form of *cis*-configured compound **4** could be obtained. Hence, the structure of **4** was identified as 10-*O*-*cis*-*p*-coumaroyl-10-hydroxyoleoside.

Symplocochinside E (**5)** (Fig. 1) was isolated as a brownish gum, and its molecular formula was established as C_27_H_32_O_15_ from the HRESI mass spectrum, which showed a sodium adduct ion peak at *m*/*z* 619.1673 [M + Na]^+^ (calcd for C_27_H_32_NaO_15_, 619.1633). The NMR spectra of **5** (Tables 1 and 2) were similar to those of **1** except for one additional methoxy group. The HMBC correlations (Supplementary Fig. S18) of OMe (*δ*_H_ 3.86, s) with the carbon at *δ*_C_ 152.9 (C-4′), of H-2′/H-6′ with C-4′ and of H-5′ with C-3′ revealed that the benzene ring had two methoxy groups at the *para* and *meta* positions. The coupling constants of H-7′ (*δ*_H_ 7.63, d, *J* = 15.9 Hz) and H-8′ (*δ*_H_ 6.39, d, *J* = 15.9 Hz) are indicative of the *trans*-configuration. Accordingly, the structure of **5** was assigned as 10-*O*-*trans*-3′,4′-dimethoxycinnamoyl-10-hydroxyoleoside.

Symplocochinside F (**6)**, a brownish gum, had the molecular formula C_28_H_34_O_16_, as deduced from the HRESI mass spectrum, which showed a deprotonated ion peak at *m*/*z* 625.1776 [M − H]^−^ (calcd for C_28_H_33_O_16_, 625.1774). The NMR spectra of **6** (Tables 1 and 2) were similar to those of **5** except for one additional methoxy groups, which is supported by the HMBC correlation (Supplementary Fig. S22) of OMe (*δ*_H_ 3.86, s) with the carbon at 154.8 (C-5′). The coupling constants of H-7′ (*δ*_H_ 7.62, d, *J* = 15.9 Hz) and H-8′ (*δ*_H_ 6.46, d, *J* = 16.0 Hz) are indicative of the *trans*-configuration. Thus, the structure of **6** was established as 10-*O*-*trans*-3′,4′,5′-trimethoxycinnamoyl-10-hydroxyoleoside.

Symplocochinside G (**8)** was obtained as a brownish gum, and its molecular formula was established as C_23_H_26_O_13_ from the HRESI mass spectrum with a peak at *m*/*z* 533.1292 [M + Na]^+^ (calcd for C_23_H_26_NaO_13_, 533.1266). In the ^1^H and ^13^C spectra of compound **8**, chemical shifts and splitting patterns of H-2′/6′ (*δ*_H_ 8.02, d, *J* = 7.2), H-3′/5′ (*δ*_H_ 7.47, t, *J* = 7.8) and H-4′ (*δ*_H_ 7.60, t, *J* = 7.5) in the aromatic regions clearly indicated the presence of a monosubstituted aromatic ring. The key HMBC correlation (Supplementary Fig. S26) of H-10 with C-7′ at *δ*_C_ 167.8 showed that the benzoic acid group is connected to C-10. Thus, the chemical structure of **8** was assigned as 10-*O*- benzoyl-10-hydroxyoleoside.

Symplocochinside H (**9)** (Fig. 1) was purified as a brownish gum, and its molecular formula was established as C_18_H_24_O_13_ from the HRESI mass spectrum with a peak at *m*/*z* 447.1149 [M − H]^−^ (calcd for C_18_H_23_O_13_, 447.1144). Analysis of the ^1^H and ^13^C-NMR data of compound **9** (Tables 1 and 2) showed similar chemical shifts except for the characteristic peaks at *δ*_C_ 170.2 and 20.7 resulting from the acetyl group. The key HMBC correlations (Supplementary Fig. S30) from the acetyl group at *δ*_H_ 2.01 and H-10 [*δ*_H_ 4.81 (dd, *J* = 13.6, 8.0 Hz) and *δ*_H_ 4.65 (m)] to the carbonyl carbon at *δ*_C_ 170.2 supported that this acetyl group is linked to C-10. Hence, the structure of **9** was elucidated as 10-*O*-acetyl-10-hydroxyoleoside.

Symplocochinside I (**10)**, a yellowish gum, had the molecular formula C_26_H_38_O_14_, as deduced from the HRESI mass spectrum, which showed a deprotonated ion peak at *m*/*z* 573.2200 [M − H]^−^ (calcd for C_26_H_37_O_14_, 573.2189). The three singlet methyl groups of H-8′ (*δ*_H_ 1.56, br s), H-9′ (*δ*_H_ 1.16, s) and H-10′ (*δ*_H_ 1.63, br s) in ^1^H-NMR spectrum of compound **10** (Table 1) indicated that these methyl groups are bound to quaternary carbons. The methylene protons of H-2′ [(*δ*_H_ 2.42, d, *J* = 13.6 Hz) and (*δ*_H_ 2.38, d, *J* = 13.7 Hz)] were geminally coupled with a large coupling constant, suggesting a lack of adjacent protons. The ^13^C-NMR and HSQC spectral data of compound **10** showed the presence of 10 peaks other than the carbon peaks of 10-hydroxyoleoside (Supplementary Figs S33 and S34), which are three sp^3^ primary carbons, three sp^3^ methylene carbons and one sp^3^ quaternary carbon with a deshielded chemical shift (*δ*_C_ 70.1) implying the presences of one oxygenated quaternary carbon, one carbonyl group, and two olefinic carbons. The HMBC correlations of H-10 with C-1′, H-9′ with C-2′/C-4′, of H-6′ with C-4′/C-5′, of H-5′ with C-3′ and of H-4′ with C-2′/C-5′ suggested the connectivity of the 10-hydroxyoleoside skeleton with 3-hydroxydimethyloctenoic acid^20,21^, which is a monoterpene also known as 3-hydroxycitronellic acid. The isolation of 3-hydroxycitronellic acid from **10** by selective hydrolysis was not successful due to racemization (data not shown). Therefore, after the reaction with dirhodium (ІІ) tetrakis (trifluoroacetate), the empirical ECD method was employed for the determination of the absolute configuration at C-3′^22^. The acetate form of compound **10** was purified after the peracetylation reaction (Supplementary Table S1) and was subjected to complexation with [Rh_2_(OCOCF_3_)_4_] in CDCl_3_. According to the bulkiness rule, the negative Cotton effect at 350 nm (band E) was observed (Fig. 3B), which means that the absolute configuration of C-3′ is 3′*R*. Thus, compound **10** was elucidated as (3′*R*)-10-*O*-3′-hydroxycitronellyl-10-hydroxyoleoside.

Symplocochinside J (**11)** was obtained as a yellowish gum, and its molecular formula was established as C_26_H_36_O_13_ from the HRESI mass spectrum which showed a deprotonated ion peak at *m*/*z* 555.2088 [M − H]^−^ (calcd for C_26_H_35_O_13_, 555.2083). The characteristic peaks of H-1 (*δ*_H_ 5.82, s)/H-3 (*δ*_H_ 7.37, s)/H-10 [(*δ*_H_ 4.82, dd, *J* = 13.8, 8.2 Hz) and (*δ*_H_ 4.67, dd, *J* = 13.3, 4.6)] and additional methyl groups at *δ*_C_ 25.49, 18.5 and 17.6 implied it is also an analogue of 10-hydroxyoleoside with a terpene (Tables 1 and 2). Compared to compound **10**, the additional double bond carbons of C-2′(*δ*_C_ 114.8)/C-3′(*δ*_C_ 160.5), the lack of any oxygenated quaternary carbon and the same small *J*-value of H-2′ (*δ*_H_ 5.68, d, *J* = 0.8)/H-9′ (*δ*_H_ 2.10, d, *J* = 0.8) indicated that the methyl group of C-9′ is bound to the sp^2^ quaternary carbon of C-3′. The key HMBC correlation (Supplementary Fig. S38) of H-9′ with C-2′/C-4′ along with the similar pattern of HMBC correlations with **10** signified that **11** is an analogue of 10-hydroxyoleoside substituted with a different monoterpene, namely, geranic acid, which is supported by the comparison with previously reported data^23^. Thus, the structure of compound **11** was elucidated as 10-*O*-geranyl-10-hydroxyoleoside.

Compound **12** (Fig. 1) was obtained as a brownish gum, and its molecular formula was established as C_13_H_20_O_3_ from the HRESI mass spectrum with a peak at *m*/*z* 225.1484 [M + H]^+^ (calcd for C_13_H_21_O_3_, 225.1485). The comparison with previously reported NMR data^24^ showed the compound has the same planar megastigmane structure (Supplementary Table S1). However, the NOESY spectrum (Supplementary Fig. S43) indicated the possibility of different configurations at C-8 and C-9 from those of the known compound 8,9-dihydromegastigmane-4,6-diene-3-one. In the NOESY spectrum, the correlations between H-7 at *δ*_H_ 6.06 (d, *J* = 9.2 Hz)/H-13 at *δ*_H_ (2.15, d, *J* = 0.8 Hz) and H-8 at *δ*_H_ (4.68, dd, *J* = 9.4, 5.3 Hz)/H-9 at *δ*_H_ (3.75, m) indicated that the olefinic carbon of C-7 is in the *E* configuration and that the relative configuration of C-8 and C-9 is [8R*, 9S*]. The specific rotation of the known compound is +54.0 (*c* 1.52 MeOH), whereas that of **12** was −88.9 (*c* 0.2 MeOH). Since the planar structure of the known compound was reported without the absolute configuration, the absolute configuration of compound **12** was determined by the helicity rule using the ECD measurement after derivatization with dimolybdenum tetraacetate^25,26^. It is possible to apply this method because compound **12** is erythro-1,2-diol and there is a bulkiness difference between the two substituents around the hydroxyl groups. The CD measurement after complexation with [Mo_2_(OAc)_4_] showed a negative CE in the band II region (403 nm, −0.15 mdeg) used as the diagnostic band (Fig. 3C). Accordingly, the structure of **12** was determined as (8*R*,9*S*)-8,9-dihydromegastigmane-4,6-diene-3-one.

Based on NMR, MS and optical rotation data and comparison with literature values, the known compounds were elucidated as, 10-cinnamoyloxyoleoside (**7**)^27^, (8*R*,9*S*)-8,9-dihydromegastigmane-4,6-diene-3-one (**12**)^24^, nigaichigoside F1 (**13**)^28^, and trachelosperoside A1 (**14**)^29^ (Fig. 1).

### Measurements of insulin mimetic activity with 2-NBDG on differentiated 3T3-L1 adipocytes

**Figure 4:**
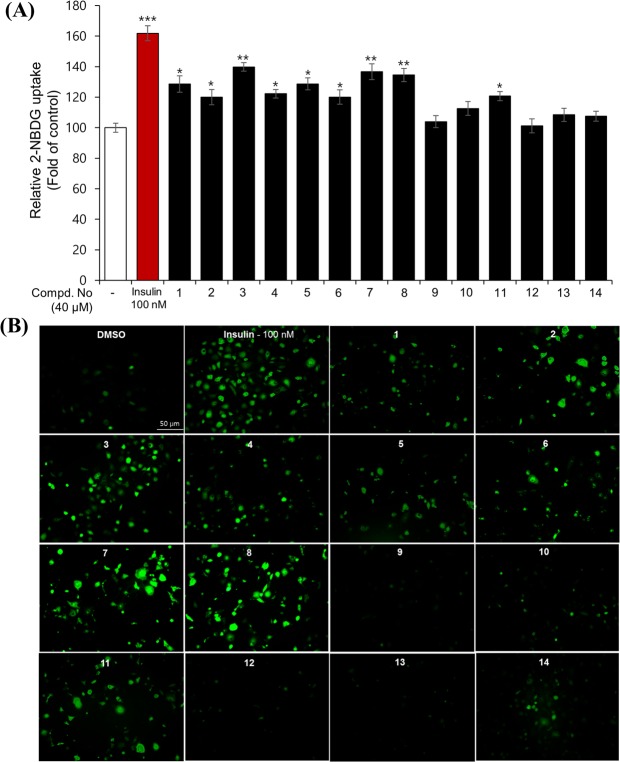
Enhancement of glucose uptake by compounds **1**–**14** (40 *μ*M) in 3T3-L1 adipocytes using a fluorescent glucose probe 2-NBDG. (**A**) After 1 hour of incubation with test compounds, the fluorescence signals were measured and the results were presented as the mean ± SD (*n* = 3), *p < 0.05 and **p < 0.01, and ***p < 0.001, compared to vehicle. (**B**) The green fluorescent signals significantly increased in the compounds treated-cells, which indicate that 2-NBDG was successfully transported into the cells.

**Figure 5:**
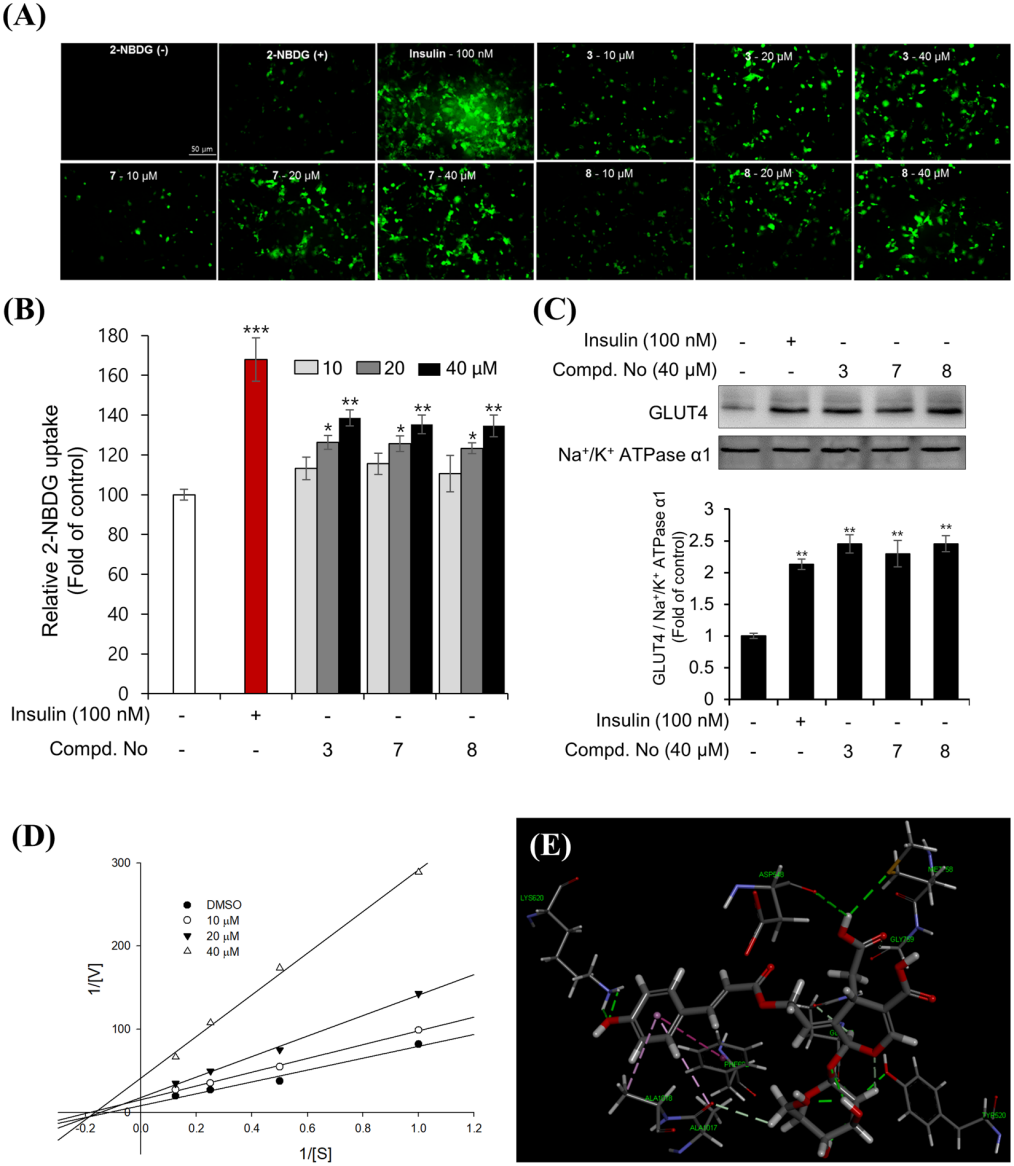
(**A**) Stimulation of the glucose uptake by compounds **3**, **7**, and **8** at different concentrations in 3T3-L1 adipocytes were obtained using the fluorescence microscopy method and (**B**) the microplate reader detection. The 3T3-L1 adipocytes were incubated with compounds for 1 hour. The cells were then captured for fluorescence images or measured fluorescence intensities. Data were calculated as the mean ± SD (*n* = 3), *p < 0.05 and **p < 0.01, and ***p < 0.001, compared to negative control. (**C**) Effect of compounds **3**, **7** and **8** on GLUT4 translocation to the plasma membrane of 3T3-L1 adipocytes. The cells were incubated with compounds for 24 hours or insulin for 2 hours. The levels of GLUT4 expression were evaluated using Western blot method. Full length western blots are provided in Supplementary Fig. S56. Results were calculated as the mean ± SD (*n* *=* 6), **p < 0.01, compared to negative control. (**D**) Graphical representation of the type of inhibition for compounds **3** at different concentrations (10, 20 and 40 *μ*M, respectively) on the PTP1B enzyme. (**E**) Docking simulation of compound **3** into the active site of PTP1B enzyme (PDB code 1Q6T) and its interaction.

Unlike the insulin in the brain, insulin at the periphery acts as an anabolic factor and causes weight gain as a side effect if the energy usage of the patient is not increased. Insulin mimetics have similar characteristics to insulin in terms of the reduction in food intake and body weight in rats when administered intracerebroventricularly. Considering the abovementioned difference, insulin memetics appear to be more advantageous than insulin because of their potential to pass through the blood-brain barrier (BBB), which allows us to find insulin mimetics from natural resources with fewer side effects. To measure for insulin mimetics, the 2-NBDG assay, which is used a fluorescent-tagged glucose analogue for monitoring the glucose uptake in cells, was introduced. All isolates (**1**–**14**) were evaluated for 2-NBDG uptake in differentiated 3T3-L1 adipocytes at a concentration of 40 *μ*M (Fig. 4A and Supplementary Fig. S49). Most of the phenolic acid- derivatized *seco*-iridoid showed activity, whereas compound **9** with an acetyl group and compound **10** with a monoterpene showed weak activity. Comparison of the activities of the compounds with the *trans* and *cis* forms showed that the *trans* isomers had stronger activity than *cis*. Among these compounds, compounds **3**, **7** and **8** showed stronger activities compared to others. Thus, fluorescent signals measurement was performed using a fluorescence microscopy for assessing the transport efficacy of 2-NBDG into cells. Increased signal intensities after treatment of the compounds were more strongly observed in cells treated with **3**, **7** and **8** at 40 *μ*M compared to those in cells of the control group (DMSO, Fig. 4B). It was also observed that selected compounds **3**, **7** and **8** increased the 2-NBDG uptake in a dose-dependent manner (Fig. 5A,B). Taken together, these results suggest that derivatization of *seco*-iridoids with *trans*-configured phenolic acids is relevant to activity. These results are consistent with the ethnopharmacological history of this plant as a diabetes remedy in Ayurvedic formulations.

### GLUT4 translocation by selected compounds in the differentiated 3T3-L1 adipocytes

Concentrations of plasma glucose levels are normally regulated by glucose transporter proteins in muscle and adipose tissue. Among the 14 sugar transporter proteins, GLUT4 is the principal isoform that plays a major role in glucose homeostasis. Intracellular glucose uptake occurs via GLUT4 recruitment to the cell surfaces of muscle and adipose cells. GLUT4 trafficking is stimulated acutely by both insulin via the PI3K/Akt pathway and exercise via the AMPK pathway^30^. Therefore, the potential of insulin mimetics can be estimated partially by the measurement of GLUT4 translocation or/and expression. Compounds **3**, **7** and **8**, which showed activity in the 2-NBDG assay, were subjected to the measurement of GLUT4 translocation in the plasma membrane. Western blot analysis showed increased GLUT4 translocation in the plasma membrane of differentiated adipocytes upon treatment with the compounds at 40 *μ*M (Fig. 5C). Compound **3** (40 *μ*M) and insulin (100 nM) clearly increased GLUT4 expression in whole cell lysate, while compounds **7** and **8** showed relatively weak effects on expression of GLUT4 in adipocytes (Supplementary Figs S52 and S57). However, compounds **3**, **7** and **8** significantly induce the expression of phosphorylated Akt (Supplementary Figs S61 and S62). These results provide insight into how these molecules stimulate glucose uptake through up-regulation of the Akt pathway. The present data strongly indicate that compound **3** promotes glucose uptake by GLUT4 translocation associated with increased level of GLUT4 expression. Although the mechanism of **3** is still unclear and further studies are in need near future, we propose this compound could regulate the glucose metabolism and insulin sensitivity.

### Protein tyrosine phosphatase 1B (PTP1B) inhibitory activity of isolated compounds

Upon insulin binding, glucose uptake is increased by a series of signals including phosphorylation of the insulin receptor (IR), transformation of phosphatidylinositol (4,5)-bisphosphate (PIP2) to phosphatidylinositol (3,4,5)-triphosphate (PIP3) by phosphatidylinositol 3-kinase (PI3K), activation of Akt, and translocation of GLUT4. PTP1B exerts negative regulation of insulin and leptin receptor signalling by dephosphorylation of activated IR and Janus kinase 2^31^. Since some *seco*-iridoids increased glucose uptake through 2-NBDG and GLUT4 translocation, the PTP1B inhibitory activity, which is important in the relevant signal transduction process, was also assessed^32^. When all isolated compounds were subjected to the PTP1B assay, compound **3** was found to be a good candidate for use as a PTP1B inhibitor, whereas **7** and **8** showed moderate activity at 50 *μ*M (Supplementary Fig. S53). Among these compounds, the IC_50_ of **3** is 19.54 ± 0.76 *μ*M, showing moderate activity compared to the positive control tested (Supplementary Fig. S54). Compound **3** was further examined in kinetic experiments and was shown to inhibit PTP1B in a non-competitive manner at different concentrations (10, 20 and 40 *μ*M, Fig. 5D). Molecular docking analysis of compound **3** into the active site of PTP1B (PDB ID code 1Q6T) was performed according to the CDOCKER protocol in the CHARMm-based docking algorithm^33^. As shown in Fig. 5E, the C-7 carboxylic acid group forms a conventional hydrogen bond and a carbon-hydrogen bond with Asp548 and GLY759, respectively. Moreover, the benzene ring shows affinity for Phe682 via a π-π interaction (Supplementary Fig. S55). These key residues have been proposed as active sites A and B of PTP1B. Additionally, the CDOCKER interaction energy was calculated to be −61.18 kcal/mol. Overall, the observed effects appear to contribute to the inhibitory activity of compound **3** against PTP1B.

## Conclusions

In this paper, eight new analogues of phenolic acid-conjugated 10-hydroxyoleoside type (**1**–**6** and **8**–**9**) and two new monoterpene-conjugated compounds (**10** and **11**), along with one known *seco*-iridoid (**7**), one megastigmane (**12**) and two triterpenoids, (**13**–**14**) were isolated from *S*. *cochinchinensis*. The absolute configurations of these compounds were determined by ECD analysis, dirhodium (ІІ) tetrakis (trifluoroacetate) reaction or dimolybdenum (ІІ) tetraacetate reaction. Compounds **3**, **7** and **8** exhibited 2-NBDG uptake increasing activity in differentiated 3T3-L1 adipocytes by GLUT4 translocation which was evident in Western blot analysis. Compound **3** increased GLUT4 expression level and direct GLUT4 translocation through the PI3K/Akt pathway via a PTP1B inhibition. These results also imply the structure activity relationship to some degree such that derivatization with a phenolic acid and *trans* configuration contribute to the activity. In conclusion, investigation of new *seco*-iridoids as ant-diabetic compounds enriches the chemical profile of *S*. *cochinchinensis* and provides evidence for the traditional ethnopharmacological uses of this plant.

## Methods

### General experimental procedures

Optical rotations were recorded on a JASCO P-2000 polarimeter (JASCO International Co. Ltd., Tokyo, Japan). ECD spectra were measured using Chirascan plus (Applied photophysics Ltd., Surrey, United Kingdom). IR data were obtained using a Nicolet 6700 FT-IR spectrometer (Thermo Electron Corp., Waltham, MA, USA). The 1D and 2D NMR spectra were obtained in deuterated solvents using an AVANCE 800 MHz spectrometer (Bruker, Germany). HRESIMS values were obtained using an Agilent Technologies 6530 Q-TOF MS spectrometer (Agilent Technologies, Inc., Santa Clara, CA, USA). Regular column chromatography (CC) was carried out with silica gel (particle size: 63–200 *μ*m, Zeochem, Lake Zurich, Switzerland), RP-C_18_ (particle size: 75 *μ*m, nacalai tesque, Kyoto, Japan), and Sephadex LH-20 (GE Healthcare, Little Chalfont, UK). Silica gel 60 F_254_ and RP-18 F_254_S TLC plates were obtained from Merck (Darmstadt, Germany). A Gilson HPLC purification system was used at a flow of 2 mL/min and UV detection at 205, 254, and 300 nm using an Optima Pak C_18_ column (10 × 250 mm, 5 *μ*m particle size; RS Tech, Seoul, Korea) and a COSMOSIL 5C_18_-MS-II column (10 × 250 mm, 5 *μ*m particle size; Nacalai Tesque, Kyoto, Japan). Analytical-grade solvents were used for extraction and isolation.

### Plant material

*S*. *cochinchinensis* was collected in Kim Boi district, Hoa Binh province, Vietnam and authenticated by Dr. Tran Van On, Head of the Department of Botany, Hanoi University of Pharmacy, Vietnam. A voucher specimen (SCL-02) was deposited at the Korea Bioactive Natural Material Bank, Research Institute of Pharmaceutical Sciences, College of Pharmacy, Seoul National University, Seoul, Republic of Korea.

### Extraction and isolation

The stems and leaves of *S*. *cochinchinensis* (4 kg) were extracted with 70% EtOH (4 × 11 L, for 4 h each) at 60 °C. The combined extract was concentrated by an evaporator to yield a dried residue (752.6 g). Dried extract was suspended in H_2_O and then partitioned with *n*-hexane, EtOAc and *n*-BuOH successively. The EtOAc portion (57.3 g) was subjected to silica gel CC (8 × 50 cm) and eluted with gradient system of *n*-hexane/acetone from 5:1 to 0:1 to yield five fractions (F.1–F.5). F.5 (12 g) was subjected to reversed-phase chromatography (5 × 40 cm) with 45% MeOH to obtain six subfractions (F.5.1–F.5.6). F.5.1 (2.93 g) was subjected to Sephadex-20 (2 × 40 cm) with 50% MeOH to obtain two subfractions (F.5.1.1–F5.1.2). Compounds **1** (9 mg), **2** (4.4 mg), **3** (11.2 mg) and **4** (3.5 mg) were purified from F.5.1.2 with ODS silica gel HPLC and eluted with MeCN/H_2_O (v/v, 19/81 to 21/79). F5.1.1 (566.4 mg) was subjected to reversed-phase chromatography (2 × 50 cm) with 35% MeOH to give six subfractions (F.5.1.1.1–F5.1.1.6). Compound **8** (4 mg) was purified from subfraction F.5.1.1.6 by ODS silica gel HPLC with MeCN/H_2_O (v/v, 24/76). F.5.1.1.1 was subjected to semi-prep HPLC with MeCN/H_2_O (v/v, 12:88), resulting in the isolation of compound **9** (16.0 mg). F.5.2 (730 mg) was subjected to reversed-phase chromatography with 40% MeOH to obtain six subfractions (F.5.2.1–F.5.2.6). F.5.2.3 was subjected to semi-prep HPLC with MeCN/H_2_O (v/v, 23:77), resulting in the isolation of compounds **5** (1.5 mg) and **6** (0.5 mg). F.5.3 was separated by Sephadex LH-20 CC into two subfractions (F.5.3.1–F5.3.2). Subfraction F.5.3.1 was subjected to semi-prep HPLC with MeCN/H_2_O (v/v, from 28/72 to 32/68), resulting in the isolation of compound **10** (3 mg). Subfraction F.5.3.2 was subjected to semi-prep HPLC with a MeCN/H_2_O (v/v, 28/72 to 32/68), resulting in the isolation of compound **7** (1.6 mg). Subfraction F.5.5.1 was subjected to Sephadex LH-20 and reversed-phase column sequentially, resulting in the isolation of compound **13** (7.7 mg). Subfraction F.5.6 was subjected to a reversed-phase column (3.5 × 50 cm) and eluted with 10% MeCN and compounds **11** (1.9 mg) and **14** (5.1 mg) were isolated from subfraction F.5.6.1 and F.5.6.2, respectively by semi-prep HPLC with MeOH/H_2_O (v/v, 55/45 to 61/39). F.2 was subjected to direct semi-prep HPLC with MeCN/H_2_O (v/v, 19/81 to 21/79), resulting in the isolation of compound **12** (2.2 mg).

#### 10-O-trans-Feruloyl-10-hydroxyoleoside (**1**)

Brownish gum; ${[{\rm{\alpha }}]}_{{\rm{D}}}^{20}$ ‒211.0 (*c* 0.10, MeOH); UV (MeOH) *λ*_max_ (log *ε*) 224 (2.3), 324 (1.9) nm; IR *ν*_max_ 3350, 2918, 2352, 1692, 1599, 1393, 1161 cm^−1^; ^1^H and ^13^C NMR, see Tables 1 and 2; HRESIMS *m*/*z* 605.1478 [M + Na]^+^ (calcd for C_26_H_30_NaO_15_, 605.1477).

#### 10-O-cis-Feruloyl-10-hydroxyoleoside (**2**)

Brownish gum; ${[{\rm{\alpha }}]}_{{\rm{D}}}^{20}$ ‒80.6 (*c* 0.10, MeOH); UV (MeOH) *λ*_max_ (log *ε*) 228 (2.4), 319 (1.9) nm; IR *ν*_max_ 3424, 2973, 2349, 1705, 1647, 1517, 1164, 1052, 1033 cm^−1^; ^1^H and ^13^C NMR, see Tables 1 and 2; HRESIMS *m*/*z* 605.1476 [M + Na]^+^ (calcd for C_26_H_30_NaO_15_, 605.1477).

#### 10-O-trans-p-Coumaroyl-10-hydroxyoleoside (**3**)

Brownish gum; ${[{\rm{\alpha }}]}_{{\rm{D}}}^{20}$ ‒124.9 (*c* 0.10, MeOH); UV (MeOH) *λ*_max_ (log *ε*) 228 (2.6), 312 (2.6) nm; IR *ν*_max_ 3407, 2980, 2349, 1747, 1658, 1516, 1171, 1052, 678, 9 cm^−1^; ^1^H and ^13^C NMR, see Tables 1 and 2; HRESIMS *m*/*z* 575.1371 [M + Na]^+^ (calcd for C_25_H_28_NaO_14_, 575.1371).

#### 10-O-cis-p-Coumaroyl-10-hydroxyoleoside (**4**)

Brownish gum; ${[{\rm{\alpha }}]}_{{\rm{D}}}^{20}$ ‒29.2 (*c* 0.10, MeOH); UV (MeOH) *λ*_max_ (log *ε*) 228 (2.5), 308 (2.3) nm; IR *ν*_max_ 3420, 2981, 2349, 1680, 1647, 1516, 1397, 1163, 1051, 671 cm^−1^; ^1^H and ^13^C NMR, see Tables 1 and 2; HRESIMS *m*/*z* 575.1381 [M + Na]^+^ (calcd for C_25_H_28_NaO_14_, 575.1371).

#### 10-O-trans-3′,4′-Dimethoxycinnamoyl-10-hydroxyoleoside (**5**)

Brownish gum; ${[{\rm{\alpha }}]}_{{\rm{D}}}^{20}$ ‒44.7 (*c* 0.50, MeOH); UV (MeOH) *λ*_max_ (log *ε*) 232 (2.2), 298 (1.9), 324 (2.0) nm; IR *ν*_max_ 2927, 2373, 1746, 1509 cm^−1^; ^1^H and ^13^C NMR, see Tables 1 and 2; HRESIMS *m*/*z* 619.1673 [M + Na]^+^ (calcd for C_27_H_32_NaO_15_, 619.1633).

#### 10-O-trans-3′,4′,5′-Trimethoxycinnamoyl-10-hydroxyoleoside (**6**)

Brownish gum; ${[{\rm{\alpha }}]}_{{\rm{D}}}^{20}$ ‒105.1 (*c* 0.50, MeOH); UV (MeOH) *λ*_max_ (log *ε*) 230 (1.8), 306 (1.6) nm; IR *ν*_max_ 3419, 2917, 2361, 1716, 1509, 1156 cm^−1^; ^1^H and ^13^C NMR, see Tables 1 and 2; HRESIMS *m*/*z* 625.1776 [M − H]^−^ (calcd for C_28_H_33_O_16_, 625.1774).

#### 10-O-Benzoyl-10-hydroxyoleoside (**8**)

Brownish gum; ${[{\rm{\alpha }}]}_{{\rm{D}}}^{20}$ ‒159.2 (*c* 0.10, MeOH); UV (MeOH) *λ*_max_ (log *ε*) 236 (2.3) nm; IR *ν*_max_ 3428, 2972, 1705, 1278, 1055, 1014, 716 cm^−1^; ^1^H and ^13^C NMR, see Tables 1 and 2; HRESIMS *m*/*z* 533.1292 [M + Na]^+^ (calcd for C_23_H_26_NaO_13_, 533.1266).

#### 10-O-Acetyl-10-hydroxyoleoside (**9**)

Brownish gum; ${[{\rm{\alpha }}]}_{{\rm{D}}}^{20}$ ‒95.1 (*c* 0.10, MeOH); UV (MeOH) *λ*_max_ (log *ε*) 226 (2.3) nm; IR *ν*_max_ 3386, 2972, 1712, 1637, 1397, 1254, 1053 cm^−1^; ^1^H and ^13^C NMR, see Tables 1 and 2; HRESIMS *m*/*z* 447.1149 [M − H]^−^ (calcd for C_18_H_23_O_13_, 447.1144).

#### (3′R)-10-O-3′-Hydroxycitronellyl-10-hydroxyoleoside (**10**)

Yellowish gum; ${[{\rm{\alpha }}]}_{{\rm{D}}}^{20}$ ‒117.9 (*c* 0.10, MeOH); UV (MeOH) *λ*_max_ (log *ε*) 226 (2.4) nm; IR *ν*_max_ 3405, 2919, 2850, 2349, 1715, 1638, 1435, 1202, 1078, 672 cm^−1^; ^1^H and ^13^C NMR, see Tables 1 and 2; HRESIMS *m*/*z* 573.2200 [M − H]^−^ (calcd for C_26_H_37_O_14_, 573.2189).

#### Tetraacetate of compound **10** (**10a**)

Colourless gum; ${[{\rm{\alpha }}]}_{{\rm{D}}}^{25}$ −113.1 (*c* 0.10, MeOH); UV (MeOH) *λ*_max_ (log *ε*) 233 (2.5) nm; IR *ν*_max_ 3271, 2898, 1748, 1646, 1508, 1224, 676 cm^−1^; ^1^H and ^13^C NMR, see supporting data; HRESIMS *m*/*z* 741.2621 [M − H]^−^ (calcd for C_34_H_45_O_18_, 741.2611).

#### 10-O-Geranyl-10-hydroxyoleoside (**11**)

Yellowish gum; ${[{\rm{\alpha }}]}_{{\rm{D}}}^{25}$ −91.1 (*c* 0.10, MeOH); UV (MeOH) *λ*_max_ (log *ε*) 222 (2.4) nm; IR *ν*_max_ 3316, 3121, 2894, 1748, 1646, 1508, 676 cm^−1^; ^1^H and ^13^C NMR, see Tables 1 and 2; HRESIMS *m*/*z* 555.2088 [M − H]^−^ (calcd for C_26_H_35_O_13_, 555.2083).

#### (8R,9S)-8,9-Dihydromegastigmane-4,6-diene-3-one (**12**)

Brownish gum; ${[{\rm{\alpha }}]}_{{\rm{D}}}^{20}$ ‒88.9 (*c* 0.20, MeOH); UV (MeOH) *λ*_max_ (log *ε*) 209 (2.3), 285 (2.6) nm; IR *ν*_max_ 3400, 2972, 2360, 1649, 1387, 1033, 671 cm^−1^; ^1^H and ^13^C NMR, see Tables 1 and 2; HRESIMS *m*/*z* 225.1484 [M + H]^+^ (calcd for C_13_H_21_O_3_, 225.1485).

### Determination of absolute configuration of sugars

Compound **9** (1.0 mg) was hydrolysed by 0.5 M HCl (1.0 mL) at 90 °C for 1 hour^35^. The solvent was neutralized with Na_2_CO_3_ and concentrated *in vacuo*. L-Cysteine methyl ester hydrochloride in anhydrous pyridine (0.5 mg) was added to the resulting residue, followed by heating for 1 hour at 60 °C. Phenyl isothiocyanate (0.1 mL) was added and heated at 60 °C for 1 hour. The solution was then analyzed by reversed-phase HPLC under the following conditions: an INNO C_18_ column (120 Å, 4.6 × 250 mm, 5 *μ*m); MeCN/H_2_O mobile phase (27:73, v/v); a diode array detector; a detection wavelength of 254 nm; and a flow rate of 0.6 mL/min. Comparisons of the retention time of the derivative of compound **9** with that of the derivative of an authentic sample of D-glucose (retention time: 23.18 min) proved the D-configuration of the glucose moiety in compound **9**.

### Absolute configuration of the tertiary alcohol moiety in 10

Compound **10** (13.2 mg) was kept in pyridine/Ac_2_O 1:1 (4 mL) at room temperature for 19 hours to give peracetylated compound **10a**. The mixture was neutralized with NaHCO_3_ and dried under vacuum. A colorless gum (10.6 mg, 80.3%) was obtained by extraction with EtOAc and the product was further purified to over 99% using prep-HPLC. Compound **10a** (0.5 mg) was then dissolved in a dry solution of [Rh_2_(OCOCF_3_)_4_] (1.0 mg) in CDCl_3_ (600 *μ*L). The resulting mixture was used for CD measurements and the obtained CD spectrum was compared with that of compound **10a** for clarity. The Cotton effect at 350 nm (E band) was correlated with the absolute configuration of the tertiary alcohol^36^.

### Preparation of Mo_2_-complexes of compound 12

The CD spectra were measured at room temperature in DMSO with 1.0 nm/step scans using a 2 mm cell over the range of 250–650 nm according to the Snatzke’s method^25^. To form complexes, compound **12** (0.18 mg, 1.33 mM/L) was dissolved in a solution of [Mo_2_(OAc)_4_] (0.34 mg, 1.33 mM/L) in DMSO at a 1/1 ratio of the molybdenum complex to the diol.

### Measurement of glucose uptake using 2-NBDG in differentiated 3T3-L1 adipocytes

To determine the level of glucose uptake into 3T3-L1 adipocytes, a fluorescent derivative of glucose (2-NBDG) (Invitrogen, OR, USA) was used as previously described with slight modifications^34,37^. First, 3T3-L1 preadipocytes were differentiated by Dulbecco’s Modified Eagle’s Medium (DMEM) (HyClone, IL, USA) containing 10% fetal bovine serum (FBS) (Gibco, NY, USA), 1 *μ*M dexamethasone (Sigma, MO, USA), 520 *μ*M 3-isobutyl-1-methyl-xanthine (Sigma, MO, USA) and 1 *μ*g/mL insulin (Roche, Germany). After 2 days of incubation, the cells were replaced with fresh media [10% FBS, 1 *μ*g/mL insulin, 100 U/mL penicillin and 100 *μ*g/mL streptomycin (Gibco, NY, USA)] and allowed to continue differentiating for 4–6 days. The glucose uptake assay was carried out as follows. 3T3-L1 adipocytes were seeded onto 96 well plates using glucose-free media supplemented with 10% FBS and incubated for 1 day. The cells were then incubated with media containing the test compounds and with or without 2-NBDG. After incubation for 1 hour at 37 °C, the cells were washed with cold PBS, and fluorescence measurements were obtained using a fluorescence microplate reader (Spectra Max GEMINI XPS, Molecular Devices, Sunnyvale, CA, USA) at ex/em = 450/535 nm. To capture bright and fluorescence images, 3T3-L1 adipocytes were maintained on sterilized glass coverslips in glucose-free media containing 10% FBS. The glucose uptake assay was performed as described above (Supplementary Figs S50 and S51). After washing with cold PBS, the cells were replaced with PBS containing 1% BSA (Sigma, MO, USA) and the slides were analysed by fluorescence microscopy (Olympus ix70 Fluorescence Microscope, Olympus Corporation, Tokyo, Japan).

### Western blot analysis for GLUT4 translocation and expression

3T3-L1 adipocytes were grown in 6-well plates with DMEM containing 10% FBS (Gibco, NY, USA). The cells were then incubated with the tested compounds for 24 hours or insulin (as a positive control) for 2 hours using serum free media. To evaluate GLUT4 expression in the plasma membrane, the plasma membrane fraction was isolated from the whole cell protein extract as previously described with slight modifications^38^. The clear isolation of the plasma membrane fraction was verified by comparing the expression of *β*-actin protein by Western blotting in the whole cell lysates and the plasma membrane fractions (Supplementary Fig. S59). The whole cell lysate was lysed using RIPA buffer containing protease and phosphatase inhibitors. Protein concentrations were measured using a BCA protein assay kit (Bio-Rad Laboratories, Inc., CA, USA). Equal amounts of proteins were then electrophoresed on 12% SDS-polyacrylamide gels and then transferred to polyvinylidene fluoride (PVDF) membranes (PVDF 0.45 *µ*m, Immobilon-P, USA). After blocking with 5% skim milk for 1 hour, the membrane was incubated overnight with different primary antibodies for GLUT4 (Santa Cruz, CA, USA), Na^+^/K^+^ ATPase α1 (Cell Signaling, MA, USA) or mouse monoclonal *β*-actin (Thermo Fisher Scientific, Rockford, IL, USA). The membrane was then incubated with HRP-conjugated secondary antibodies for 2 hours at room temperature. The membrane was detected using a LAS 4000 luminescent image analyzer (Fuji Film, Tokyo, Japan).

### Western blot analysis of Akt phosphorylation

3T3-L1 adipocytes were treated with compound **3** for different time points (Supplementary Fig. S60) or test compounds for 2 hours using serum free media. Then, cell lysates were collected using a lysis buffer [50 mM Tris-HCl (pH 7.6), 120 mM NaCl, 1 mM EDTA, 0.5% NP-40, 50 mM NaF]. Western blotting was performed as described above. The membranes were incubated with the primary antibodies for *p*-Akt (Ser473) and Akt (Cell Signaling, MA, USA) or mouse monoclonal *β*-actin (Thermo Fisher Scientific, Rockford, IL, USA).

### PTP1B inhibition and kinetic assay

PTP1B enzyme (human, recombinant) was purchased from BIOMOL International LP (USA) and the enzyme assay was performed as previously described^34^. Briefly, the assay was performed in 96-well plate containing 4 mM *p*-NPP and PTP1B (0.05–0.1 *μ*g) in enzyme buffer [50 mM citrate (pH 6.0), 0.1 M NaCl, 1 mM dithiothreitol (DTT), and 1 mM EDTA] with or without test compounds in triplicate. In addition, an assay was carried out to exclude the fluorescence effect of the test compounds in the absence of PTP1B enzyme. After incubation at 37 °C for 30 minutes, the reaction was terminated with 10 M NaOH solution. The *p*-nitrophenol product was then measured at 405 nm using an absorbance microplate reader (VersaMax^TM^, Randor, PA, USA). The enzyme kinetics were determined using Lineweaver-Burk plots. The PTP1B inhibition mode was examined at different concentrations of the *p*-NPP substrate (from 1 to 8 mM) in the absence and presence of the test compounds (10, 20 and 40 *μ*M). Half-maximal inhibitory concentration (IC_50_) values and inhibition type of PTP1B were calculated by Sigma Plot 10.0 (Systat Software Inc., San Jose, CA, USA).

### Molecular docking simulation

Docking studies between the structure of the PTP1B protein and compound **3** were successfully performed using Discovery Studio 4.0/CDOCKER software (Accelrys, San Diego, CA). The PTP1B protein structure was obtained from the Protein Data Bank (http://www.pdb.org) (PDB ID code: 1Q6T). Protein-ligand binding affinities were optimized and calculated according to the CDOCKER interaction energy arising from several interacting bonds, such as conventional hydrogen bonds, carbon-hydrogen bond, π-π T-shaped and π-alkyl interactions.

### Statistical analysis

Data were calculated as the mean ± SD of three independent experiments. The mean value of difference groups was calculated using ANOVA, which was carried out using SPSS Statistics 23 (SPSS, Inc., Chicago, IL, USA). IC_50_ values and inhibition modes were determined by Sigma Plot 10.0 software (Systat Software Inc., San Jose, CA, USA). Statistically significant *p* values were established at *p < 0.05, **p < 0.01, and ***p < 0.001.

## Supplementary information

SUPPLEMENTARY DATA

## Data Availability

All data generated or analysed for this study are included in this published paper (and its Supplementary Information files).
