# Supplementary material for: Hydroxyoleoside-type seco-iridoids from Symplocos cochinchinensis and their insulin mimetic activity
Source: Sci Rep. 2019 Feb 19;9:2270. doi: 10.1038/s41598-018-38013-4 (PMC6381099; doi:10.1038/s41598-018-38013-4)
Supplement: Supplementary file 1 — SUPPLEMENTARY DATA [file 41598_2018_38013_MOESM1_ESM.docx]

**SUPPLEMENTARY DATA**

**Hydroxyoleoside-type *seco*-iridoids from *Symplocos cochinchinensis* and their insulin mimetic activity**

Ba-Wool Lee^1^, Thi Kim Quy Ha^1^, Ha Thanh Tung Pham^1^, Quynh Hoa Hoang^2^, Van On Tran^2^ & Won Keun Oh^1,*^

^1^Korea Bioactive Natural Material Bank, Research Institute of Pharmaceutical Sciences, College of Pharmacy, Seoul National University, Seoul 151-742, Republic of Korea

^2^Department of Botany, Hanoi University of Pharmacy, Hanoi, Vietnam

*To whom correspondence should be addressed. Tel and Fax: +82-02-880-7872. E-mail: wkoh1@snu.ac.kr. .

**TABLE OF CONTENTS**

**Figure S1:** ^1^H and ^13^C NMR spectra of compound **1** (DMSO-*d*_6_).

**Figure S2:** HSQC and HMBC spectra of compound **1** (DMSO-*d*_6_).

**Figure S3:** ROESY NMR spectrum of compound **1** (DMSO-*d*_6_).

**Figure S4:** HR-ESIMS of compound **1**.

**Figure S5:** ^1^H and ^13^C NMR spectra of compound **2** (DMSO-*d*_6_).

**Figure S6:** HSQC and HMBC spectra of compound **2** (DMSO-*d*_6_).

**Figure S7:** NOESY NMR spectrum of compound **2** (DMSO-*d*_6_).

**Figure S8:** HR-ESIMS of compound **2.**

**Figure S9:** ^1^H and ^13^C NMR spectra of compound **3** (DMSO-*d*_6_).

**Figure S10:** HSQC and HMBC spectra of compound **3** (DMSO-*d*_6_).

**Figure S11:** NOESY NMR spectrum of compound **3** (DMSO-*d*_6_).

**Figure S12:** HR-ESIMS of compound **3.**

**Figure S13:** ^1^H and ^13^C NMR spectra of compound **4** (DMSO-*d*_6_).

**Figure S14:** HSQC and HMBC spectra of compound **4** (DMSO-*d*_6_).

**Figure S15:** NOESY NMR spectrum of compound **4** (DMSO-*d*_6_).

**Figure S16:** HR-ESIMS of compound **4.**

**Figure S17:** ^1^H and ^13^C NMR spectra of compound **5** (CD_3_OD).

**Figure S18:** HSQC and HMBC spectra of compound **5** (CD_3_OD).

**Figure S19:** ROESY NMR spectrum of compound **5** (CD_3_OD).

**Figure S20:** HR-ESIMS of compound **5.**

**Figure S21:** ^1^H and ^13^C NMR spectra of compound **6** (CD_3_OD).

**Figure S22:** HSQC and HMBC spectra of compound **6** (CD_3_OD).

**Figure S23:** ROESY NMR spectrum of compound **6** (CD_3_OD).

**Figure S24:** HR-ESIMS of compound **6.**

**Figure S25:** ^1^H and ^13^C NMR spectra of compound **8** (CD_3_OD).

**Figure S26:** HSQC and HMBC spectra of compound **8** (CD_3_OD).

**Figure S27:** NOESY and 1D-ROE NMR spectra of compound **8** (CD_3_OD).

**Figure S28:** HR-ESIMS of compound **8.**

**Figure S29:** ^1^H and ^13^C NMR spectra of compound **9** (DMSO-*d*_6_).

**Figure S30:** HSQC and HMBC spectra of compound **9** (DMSO-*d*_6_).

**Figure S31:** NOESY NMR spectrum of compound **9** (DMSO-*d*_6_).

**Figure S32:** HR-ESIMS of compound **9.**

**Figure S33:** ^1^H and ^13^C NMR spectra of compound **10** (DMSO-*d*_6_).

**Figure S34:** HSQC and HMBC spectra of compound **10** (DMSO-*d*_6_).

**Figure S35:** COSY and ROESY NMR spectra of compound **10** (DMSO-*d*_6_).

**Figure S36:** HR-ESIMS of compound **10.**

**Figure S37:** ^1^H and ^13^C NMR spectra of compound **11** (DMSO-*d*_6_).

**Figure S38:** HSQC and HMBC spectra of compound **11** (DMSO-*d*_6_).

**Figure S39:** COSY and ROESY NMR spectra of compound **11** (DMSO-*d*_6_).

**Figure S40:** HR-ESIMS of compound **11.**

**Figure S41:** ^1^H and ^13^C NMR spectra of compound **12** (CD_3_OD).

**Figure S42:** HSQC and HMBC spectra of compound **12** (CD_3_OD).

**Figure S43:** NOESY NMR spectrum of compound **12** (CD_3_OD).

**Figure S44:** HR-ESIMS of compound **12.**

**Figure S45:** Experimental CD spectrum of compound **12** (MeOH).

**Figure S46:** ^1^H and ^13^C NMR spectra of compound **10a** (DMSO-*d*_6_).

**Figure S47:** HSQC and HMBC spectra of compound **10a** (DMSO-*d*_6_).

**Figure S48:** HR-ESIMS of compound **10a.**

**Figure S49:** The effect of all compounds **1**-**14** on cytotoxicity of 3T3-L1 adipocytes**.** The cells were exposed with compounds (40 *μ*M) for 24 hours at 37 °C. The MTT assay was then performed as described in the experimental section. Data were calculated as the mean ± SD (*n* = 3), compared to the vehicle.

**Figure S50:** Effects of all compounds **1-14** (40 *μ*M) on 2-NBDG uptake in 3T3-L1 adipocytes. Images were obtained by fluorescence microscopy.

**Figure S51:** The effects of compounds **3**, **7**, and **8** at different concentrations on 2-NBDG uptake in 3T3-L1 adipocytes. Images were obtained by the fluorescence microscopy method.

**Figure S52:** The effect of compounds **3**, **7** and **8** (40 *μ*M) on the expression of GLUT4 in whole cell lysates. After treatment with the test compounds, cells were lysed using RIPA buffer and Western blot assay was carried out as described in the experimental Section. The protein expression levels were normalized against *β*-actin. Results were calculated as the mean ± SD (*n*=2), ** p* < 0.05, compared to negative control.

**Figure S53:** Inhibitory effect of all compounds **1**-**14** on PTP1B enzyme. Results were expressed as the mean ± SD of three independent experiments. Statistical significance was accepted at * *p* < 0.05, and *** *p* < 0.001, compared to the negative control.

**Figure S54:** Inhibitory effect of ursolic acid and compounds **3** on PTP1B enzyme with IC_50_ values of 4.58 ± 0.56 and 19.54 ± 0.67 *μ*M, respectively. Results were calculated as the mean ± SD (*n* = 3).

**Figure S55:** (**A**) 3D docking simulation of compound **3** into the active site of PTP1B (PDB code 1Q6T). (**B**) 2D diagram of the ligand interactions between compound **3** and the PTP1B enzyme.

**Figure S56**: The effects of compounds **3**, **7** and **8** from *S. cochinchinensis* on GLUT4 translocation to the plasma membrane of 3T3-L1 adipocytes; original uncropped blots.

**Figure S57**: The effects of compounds **3**, **7** and **8** (40 *μ*M) on the expression of GLUT4 in whole cell lysates, original uncropped blots.

**Figure S58**: Determination of the Ki value for noncompetitive inhibition of compound **3**.

**Figure S59:** Comparison the expression of *β*-actin protein in the whole cell lysates and the plasma membrane fractions were evaluated by Western blotting.

**Figure S60:** The effects of incubation time on the phosphorylation of Akt by compound **3** (40 *μ*M), original uncropped blots.

**Figure S61:** The effects of compounds **3**, **7**, and **8** (40 *μ*M) on the phosphorylation of Akt protein. After incubated with test compounds or insulin for 2 hours, 3T3-L1 adipocytes were lysed and Western blotting was performed as described in the experimental section. The protein expression levels were normalized against Akt protein. Data were calculated as the mean ± SD (*n*=4), ** p* < 0.05, compared to negative group.

**Figure S62:** The effects of compounds **3**, **7**, and **8** (40 *μ*M) on the phosphorylation of Akt in whole cell lysates, original uncropped blots.

**Table S1:** ^1^H and ^13^C NMR Data of compounds **10a** and **12** (^1^H 800 MHz and ^13^C 200 MHz).


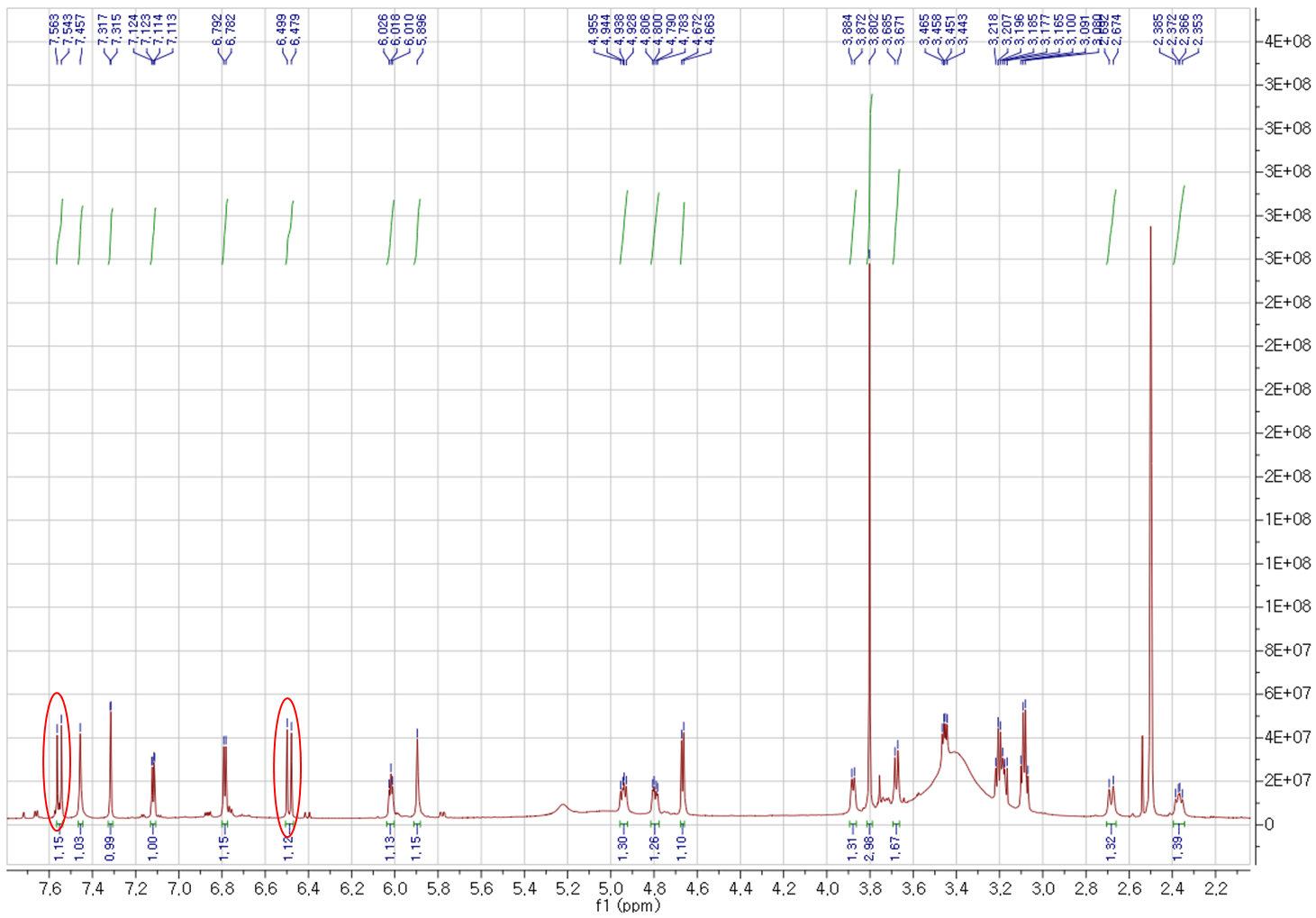


**
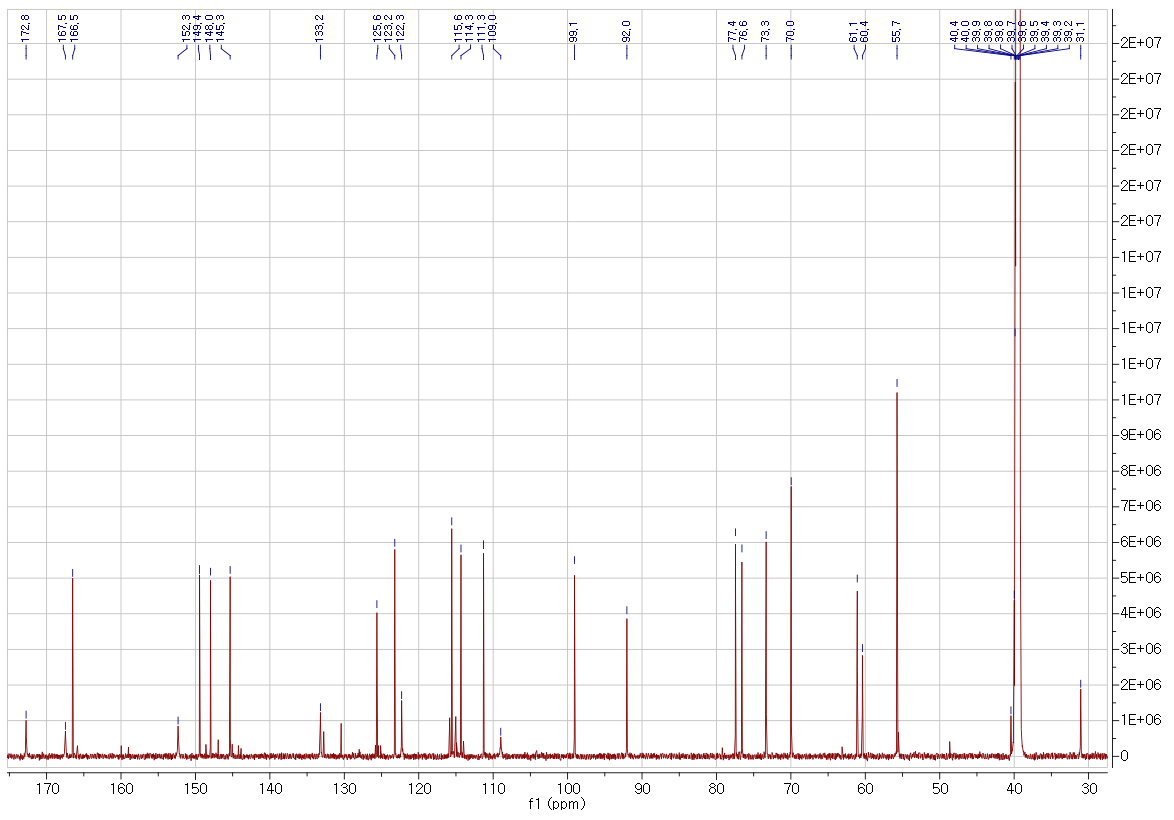
**

**Figure S1:**^1^H and ^13^C NMR spectra of compound **1** (800, 200 MHz, DMSO-*d*_6_)


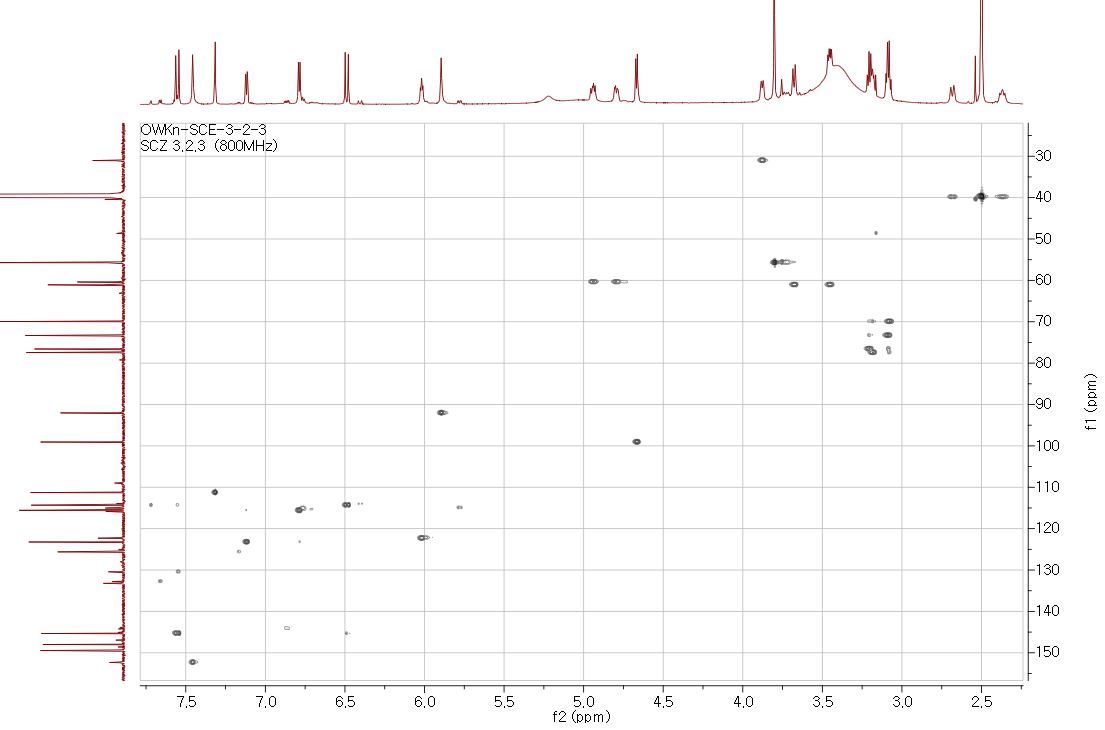


**
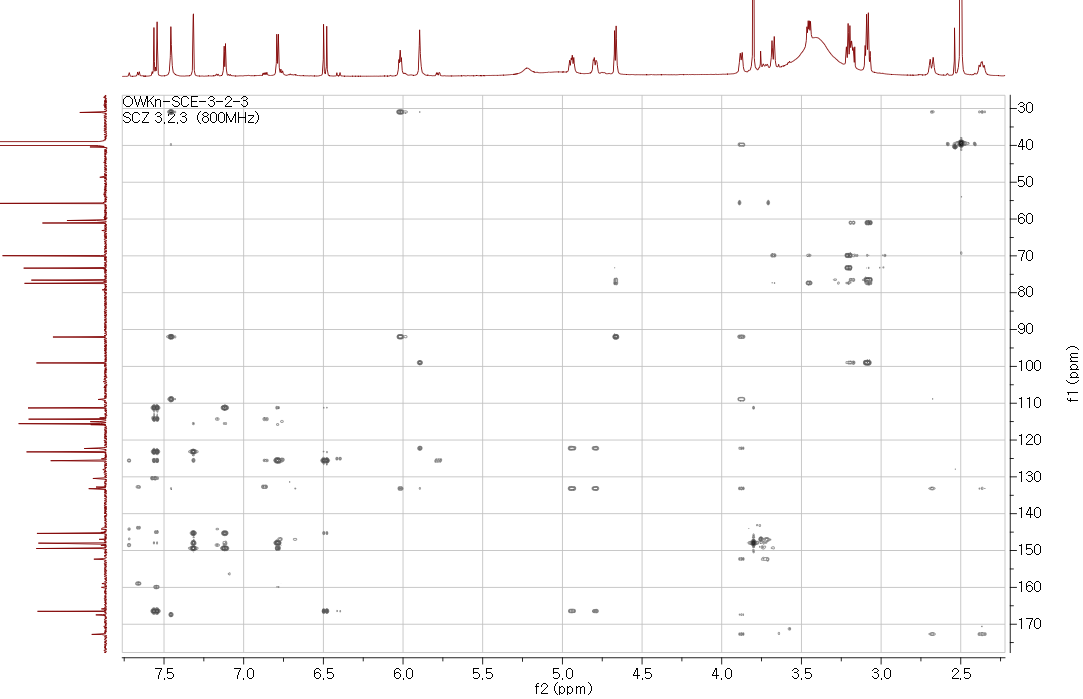
**

**Figure S2:** HSQC and HMBC NMR spectra of compound **1** (800 MHz, DMSO-*d_6_*)

**
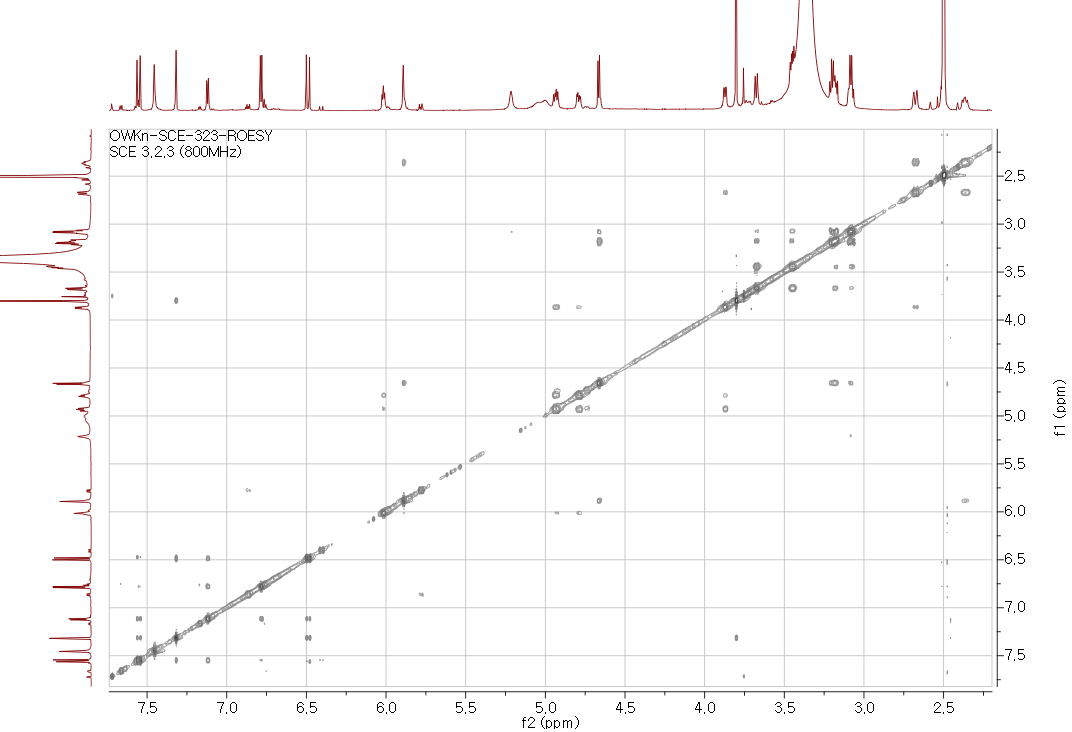
Figure S3:** ROESY NMR spectrum of compound **1** (800 MHz, DMSO-*d*_6_)

**
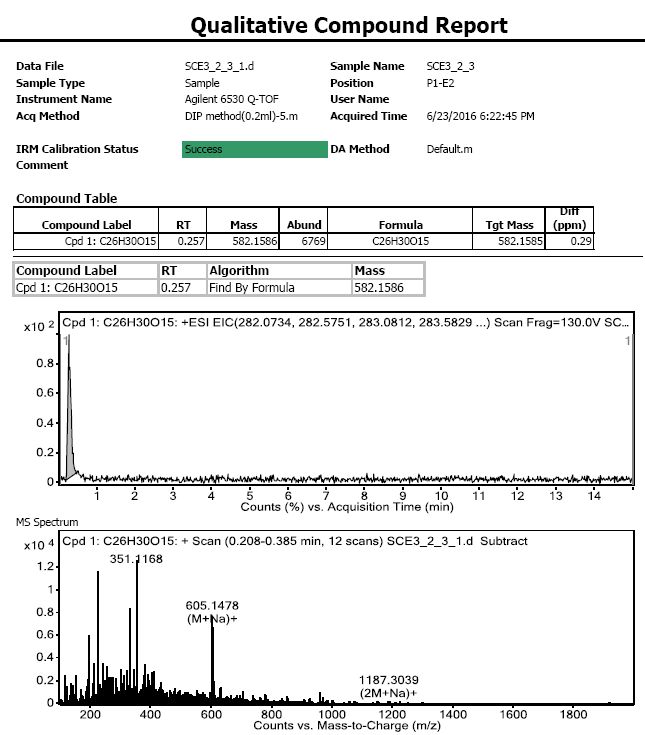
**

**Figure S4:** HR-ESI(+)MS of compound **1**


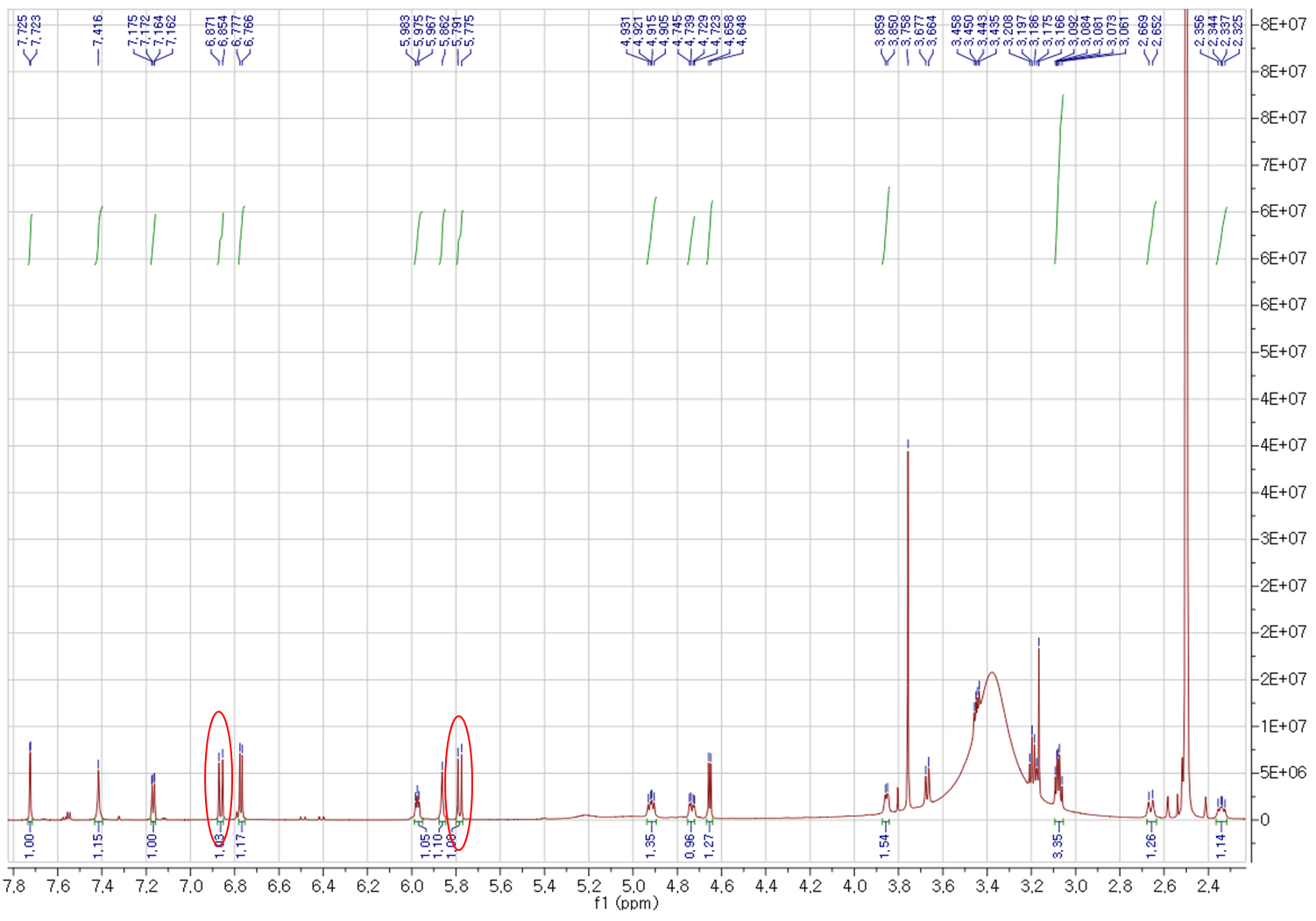


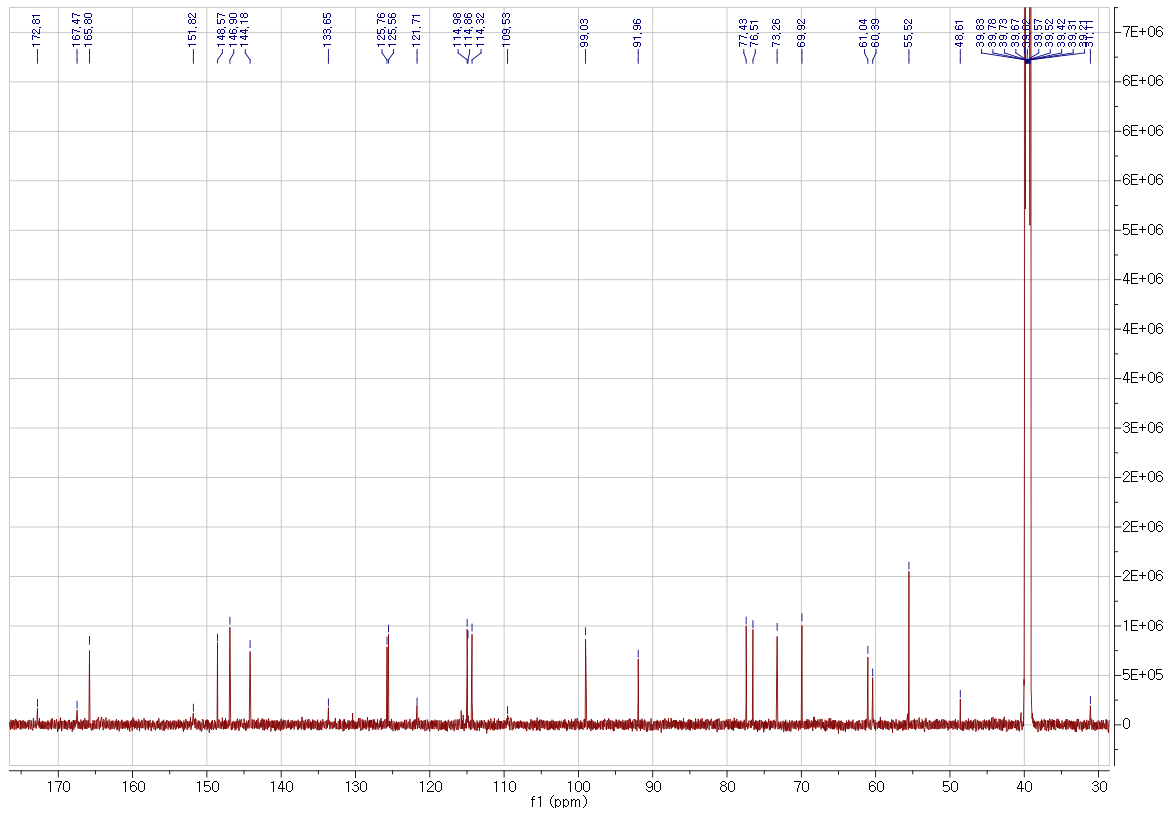


**Figure S5:** ^1^H and ^13^C NMR spectra of compound **2** (800, 200 MHz, DMSO-*d*_6_)

**
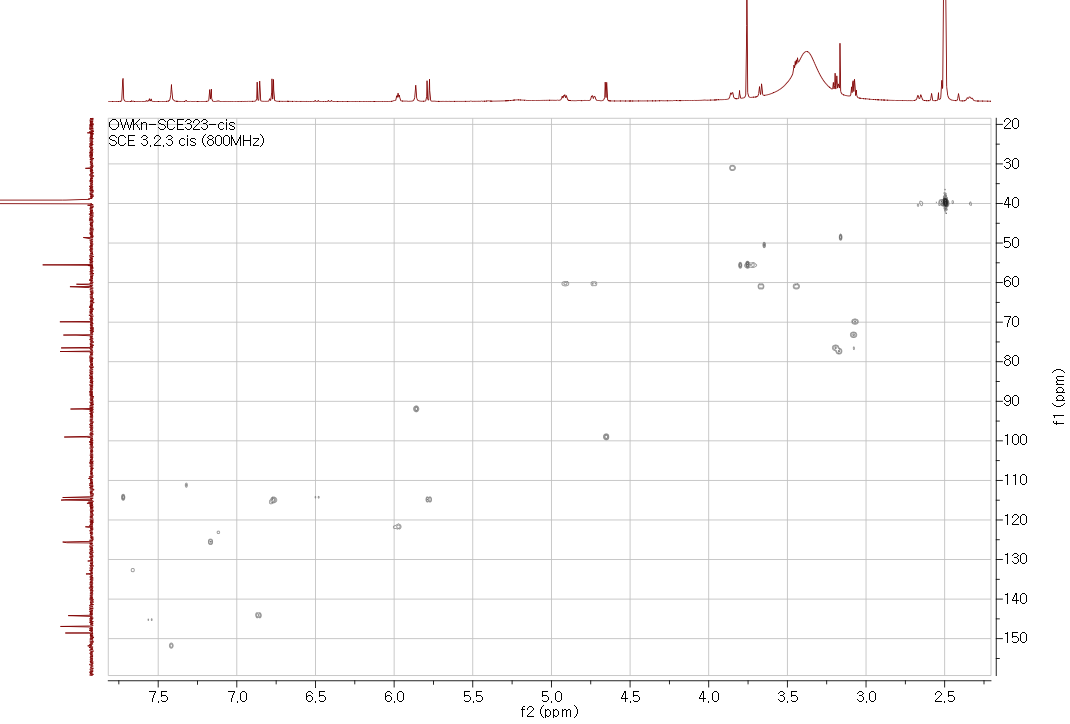
**

**
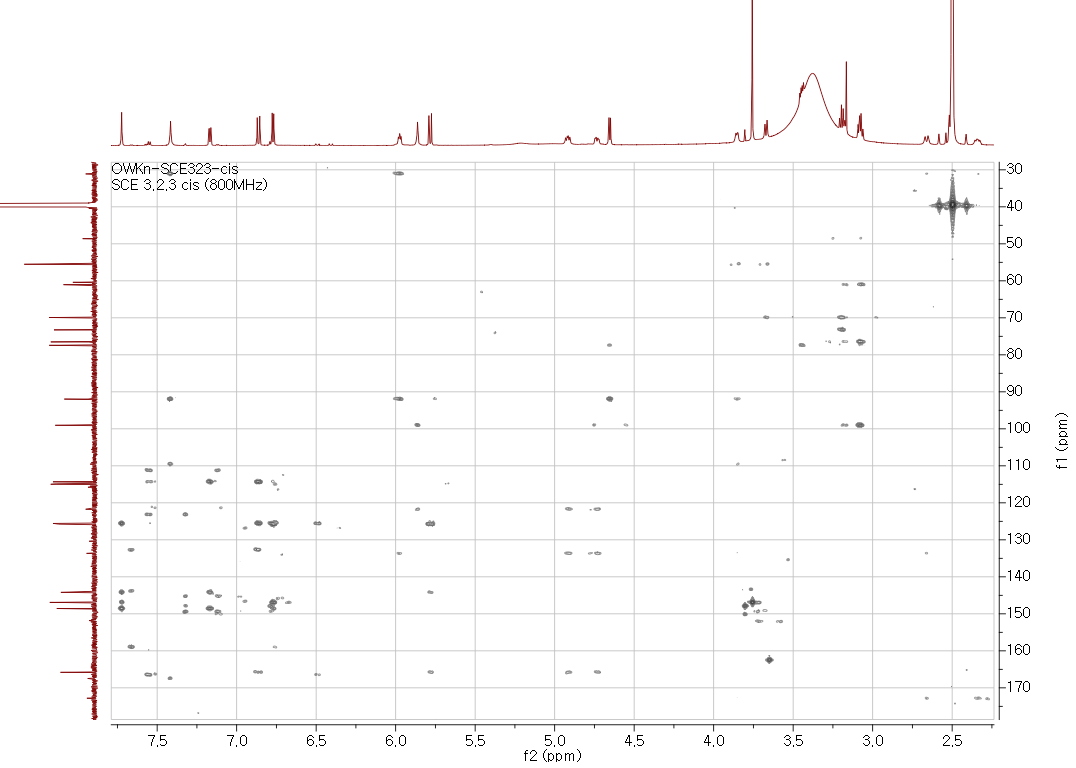
**

**Figure S6:** HSQC and HMBC NMR spectra of compound **2** (800 MHz, DMSO-*d_6_*)

**
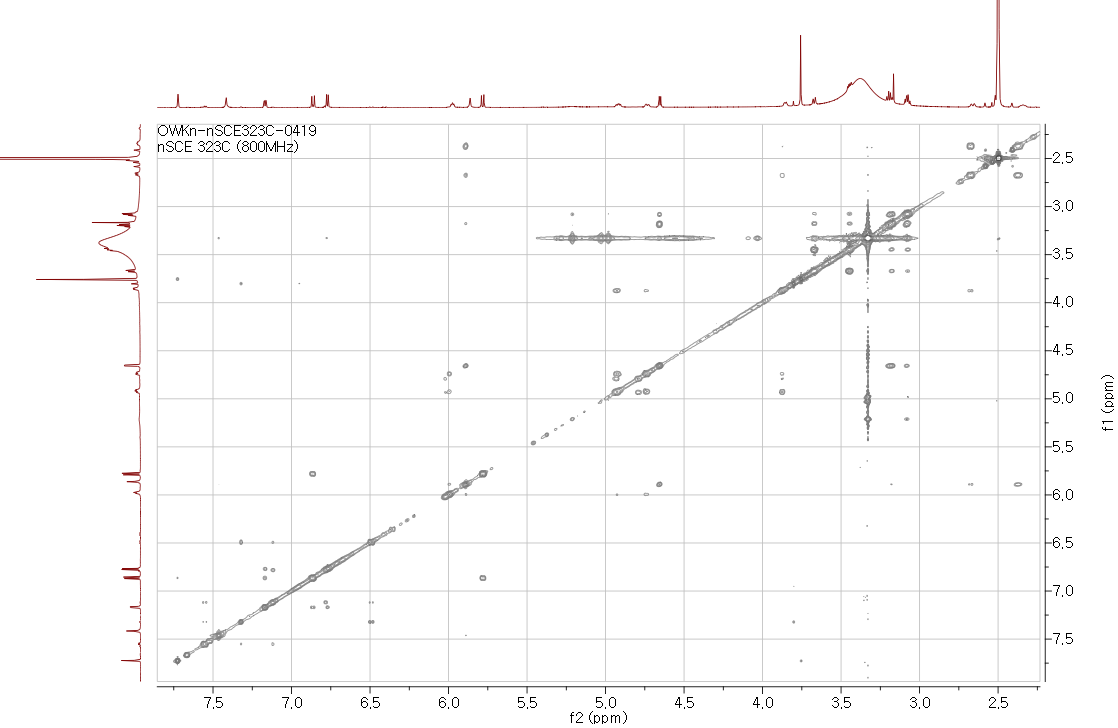
Figure S7:** NOESY spectrum of compound **2** (800 MHz, DMSO-*d*_6_)


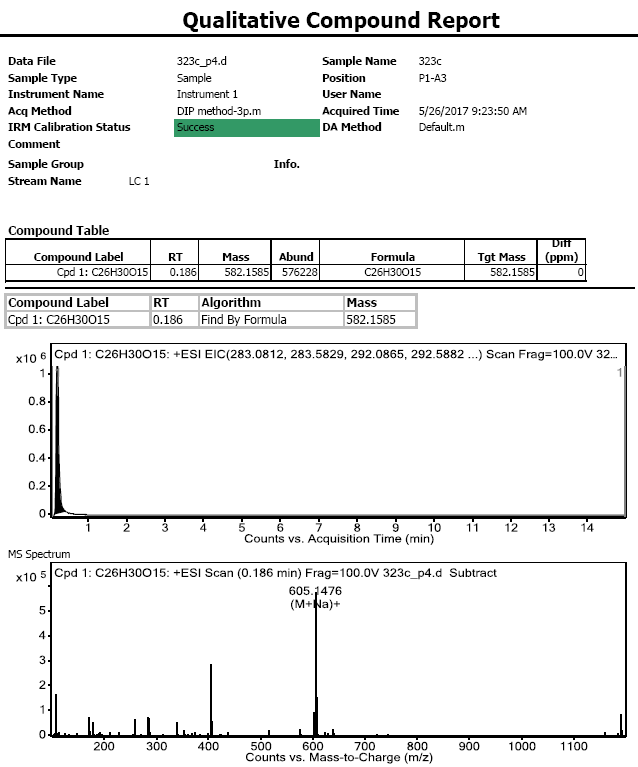


**Figure S8:** HR-ESI(+)MS of compound **2**

**
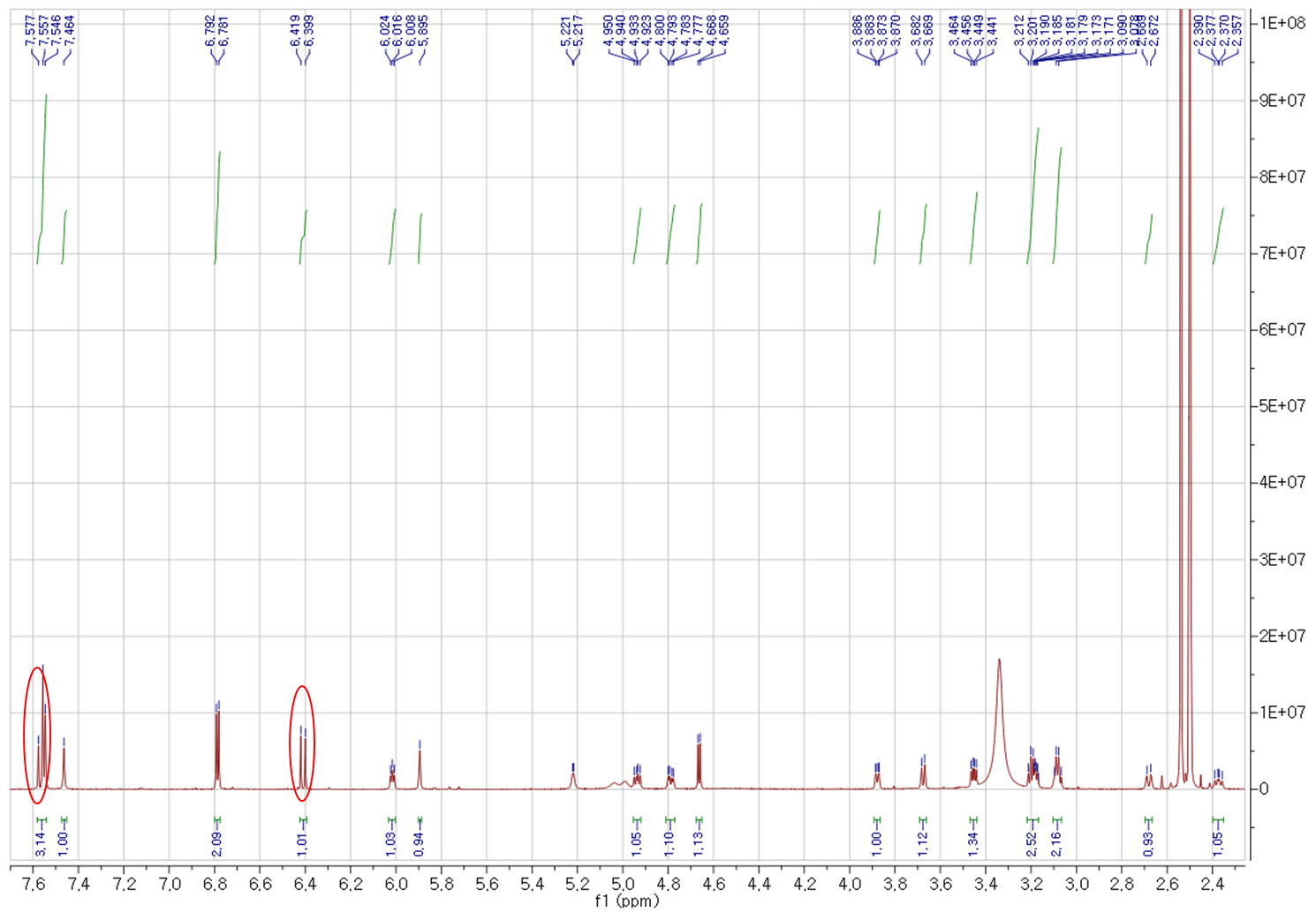
**


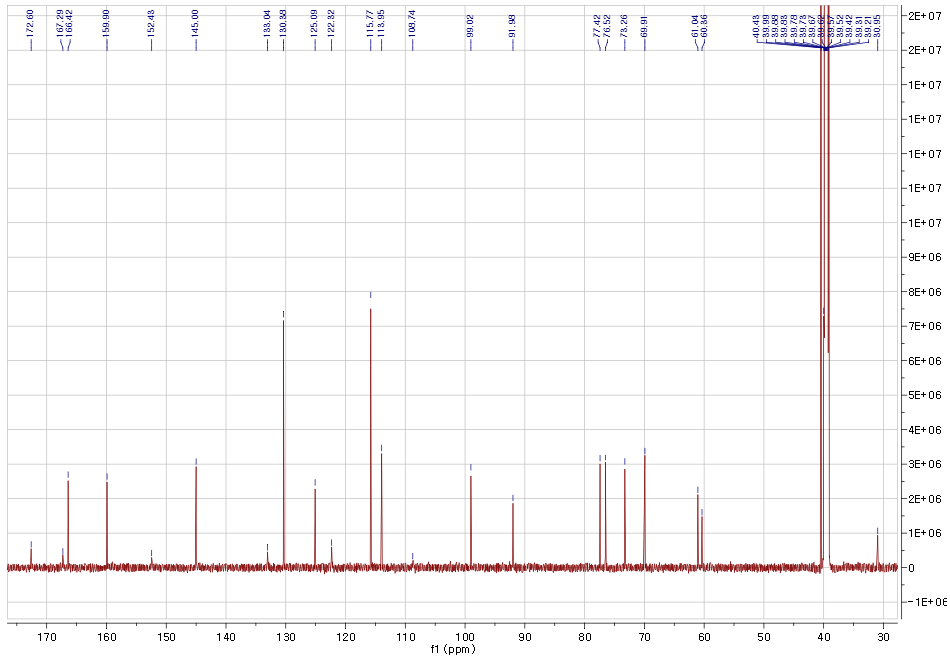


**Figure S9:** ^1^H and ^13^C NMR spectra of compound **3** (800, 200 MHz, DMSO-*d*_6_)

**
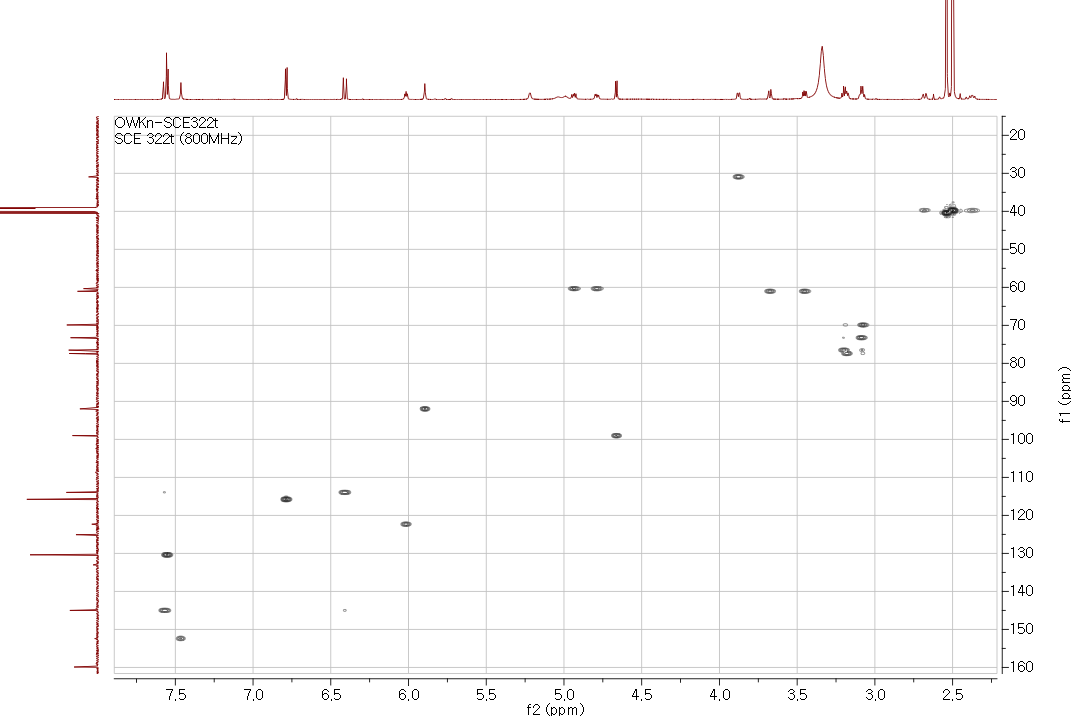
**


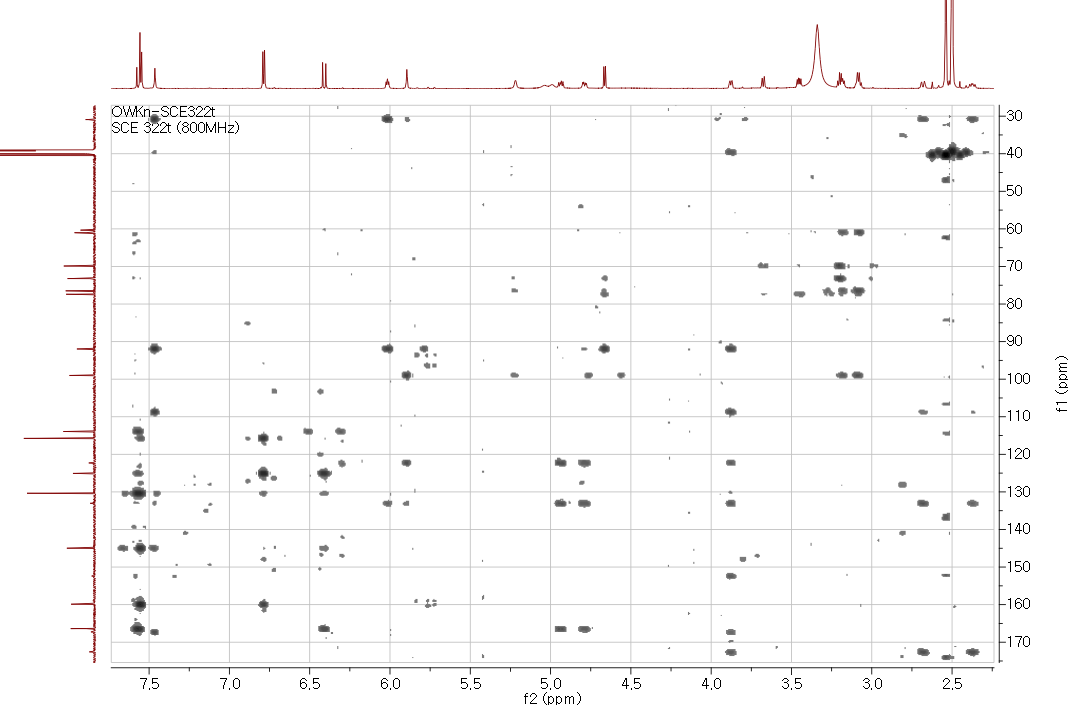


**Figure S10:** HSQC and HMBC NMR spectra of compound **3** (800 MHz, DMSO-*d_6_*)


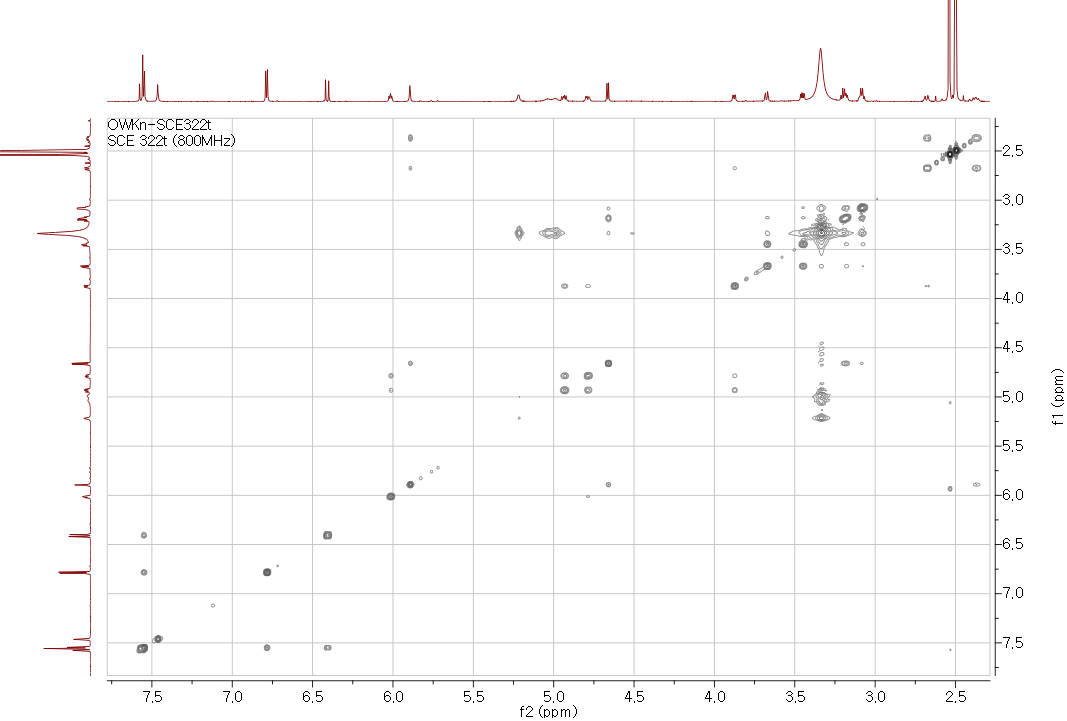


**Figure S11:** NOESY spectrum of compound **3** (800 MHz, DMSO-*d*_6_)


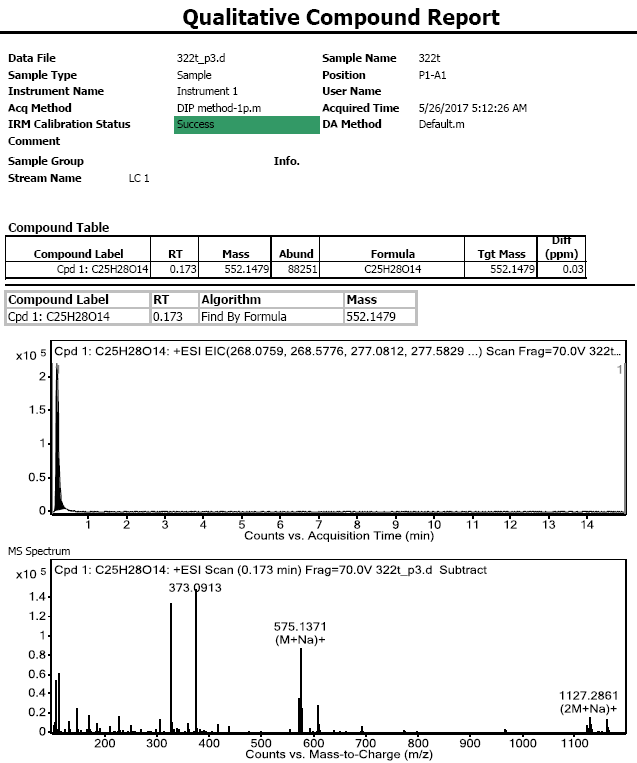


**Figure S12:** HR-ESI(+)MS of compound **3**


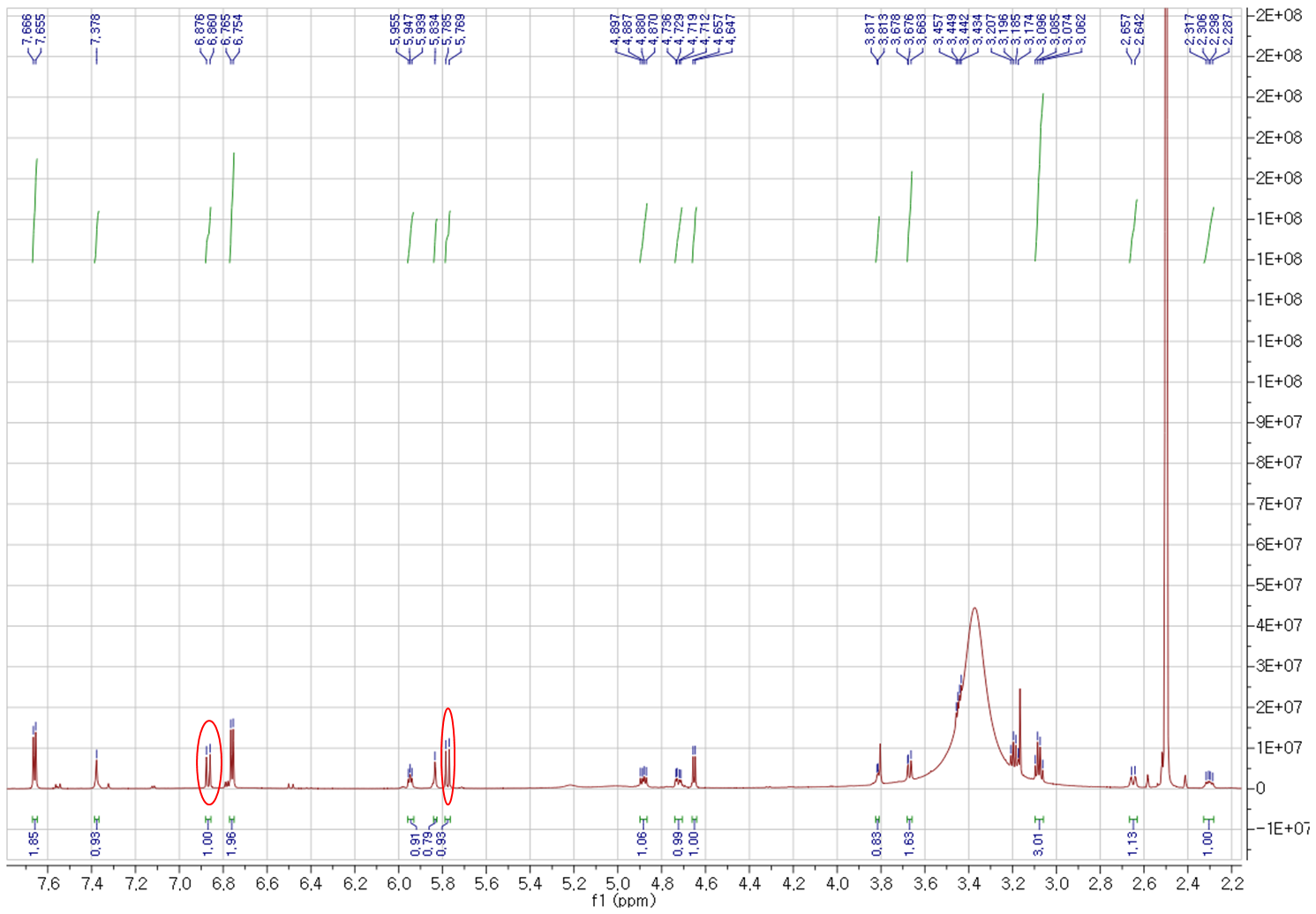


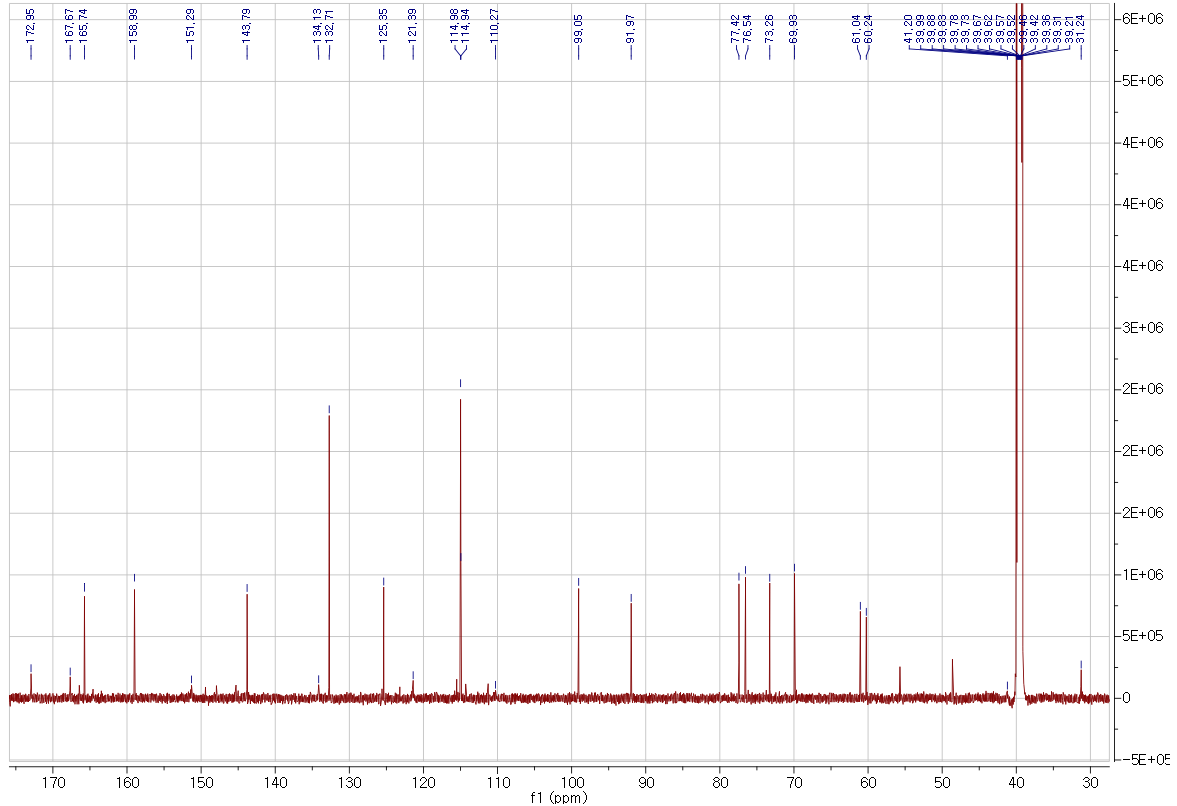


**Figure S13:** ^1^H and ^13^C NMR spectra of compound **4** (800, 200 MHz, DMSO-*d*_6_)


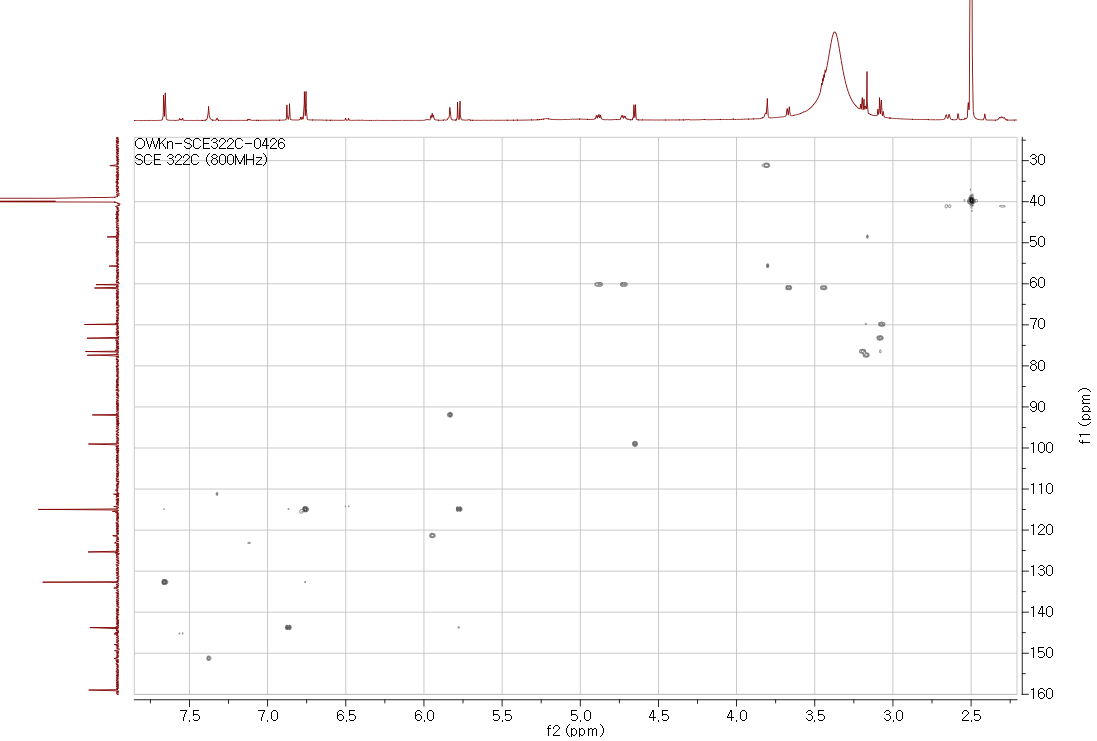


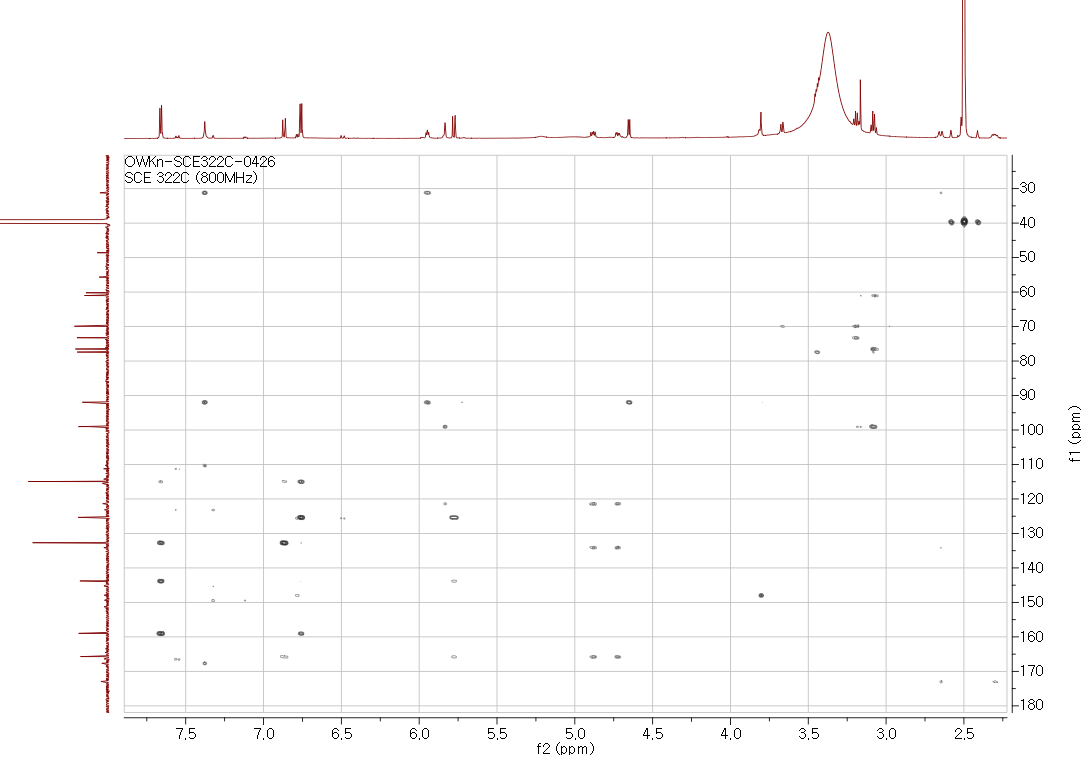


**Figure S14:** HSQC and HMBC NMR spectra of compound **4** (800 MHz, DMSO-*d_6_*)

**
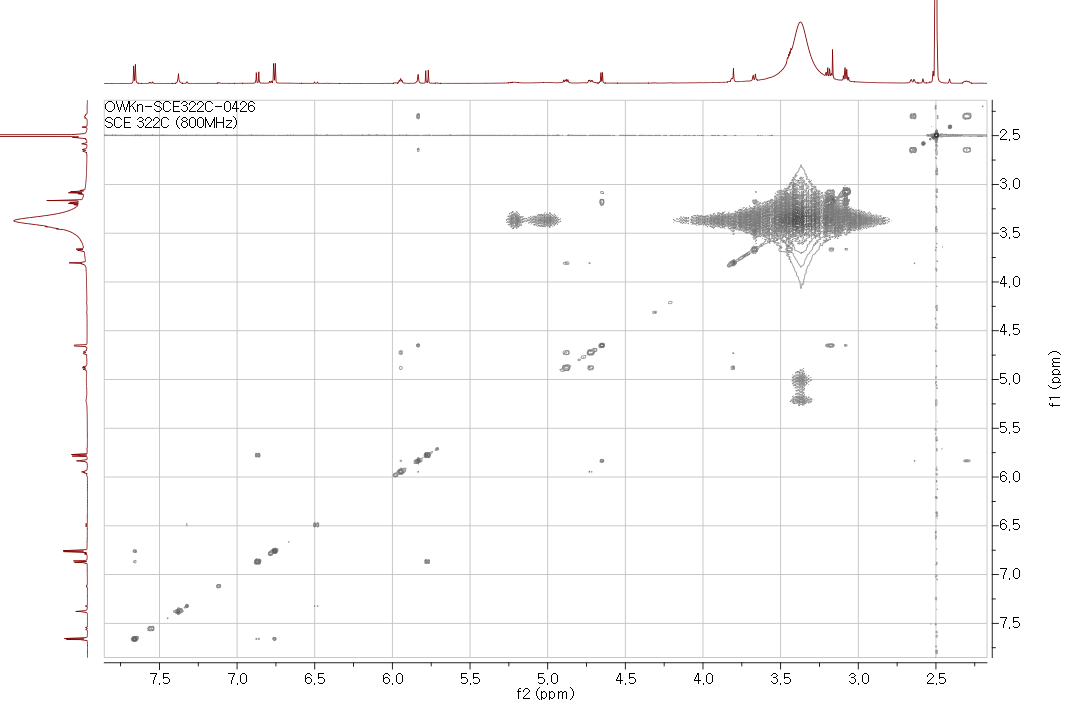
**

**Figure S15:** NOESY spectrum of compound **4** (800 MHz, DMSO-*d*_6_)


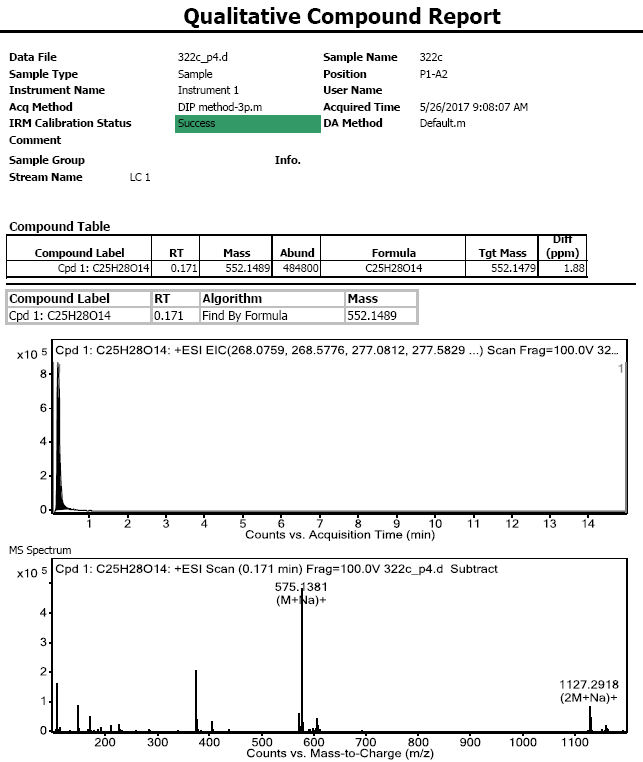


**Figure S16:** HR-ESI(+)MS of compound

**
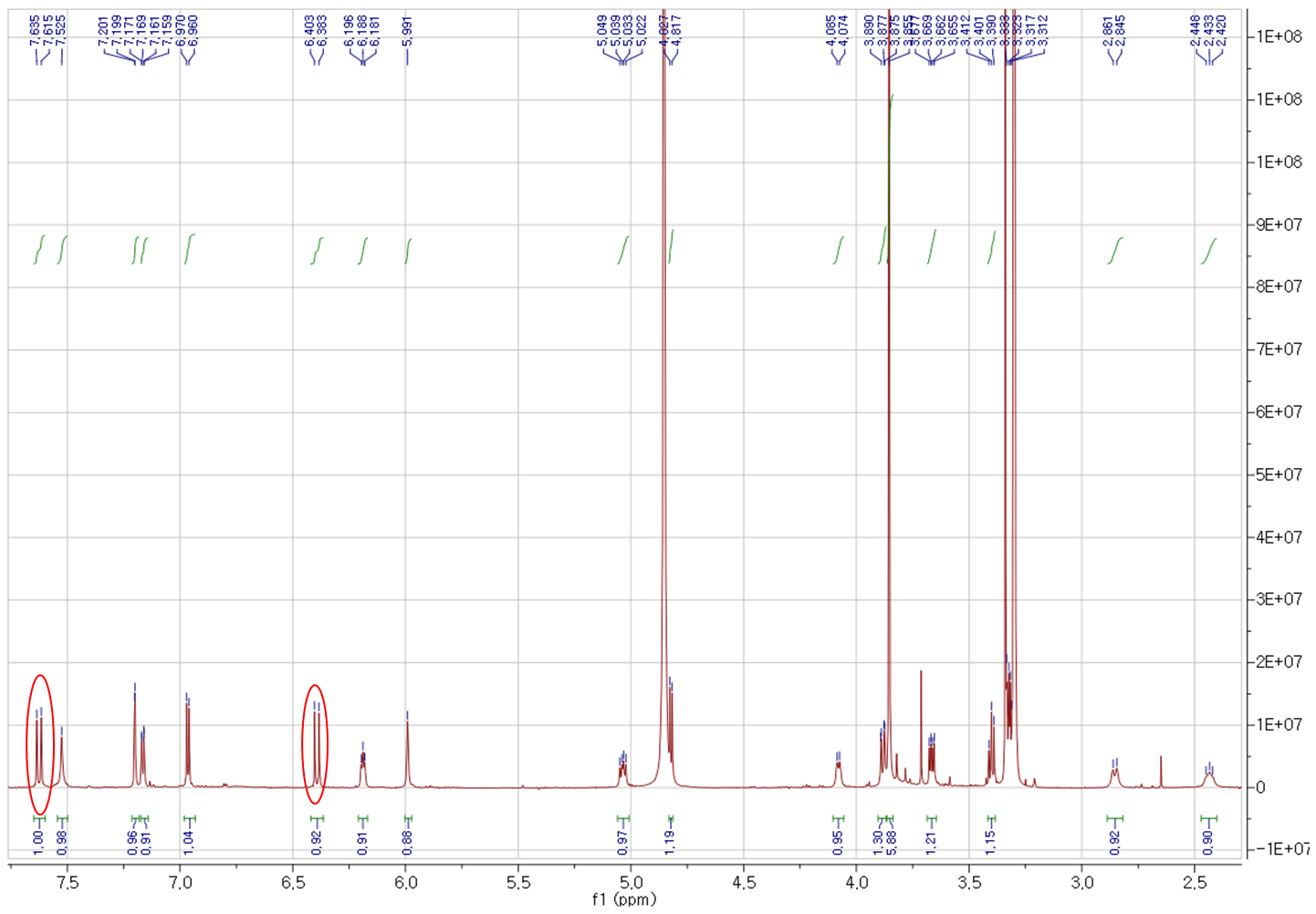
**

**
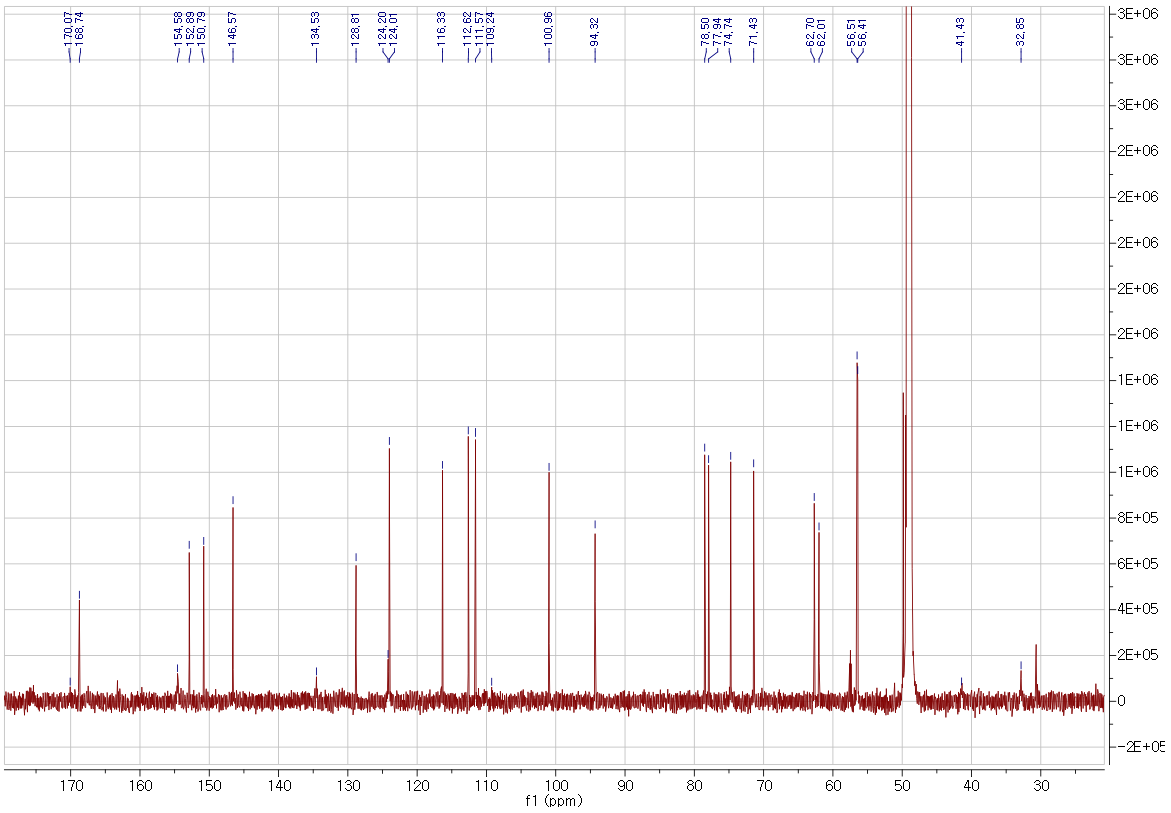
**

**Figure S17:** ^1^H and ^13^C NMR spectra of compound **5** (800, 200 MHz, CD_3_OD)

**
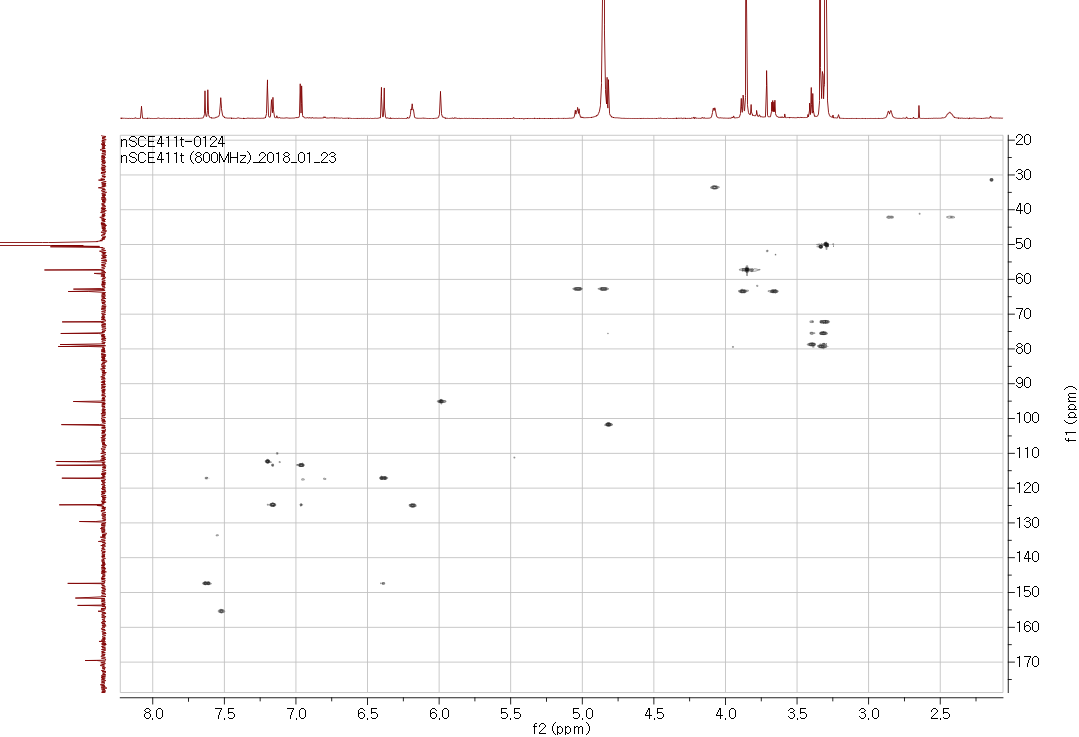
**

**
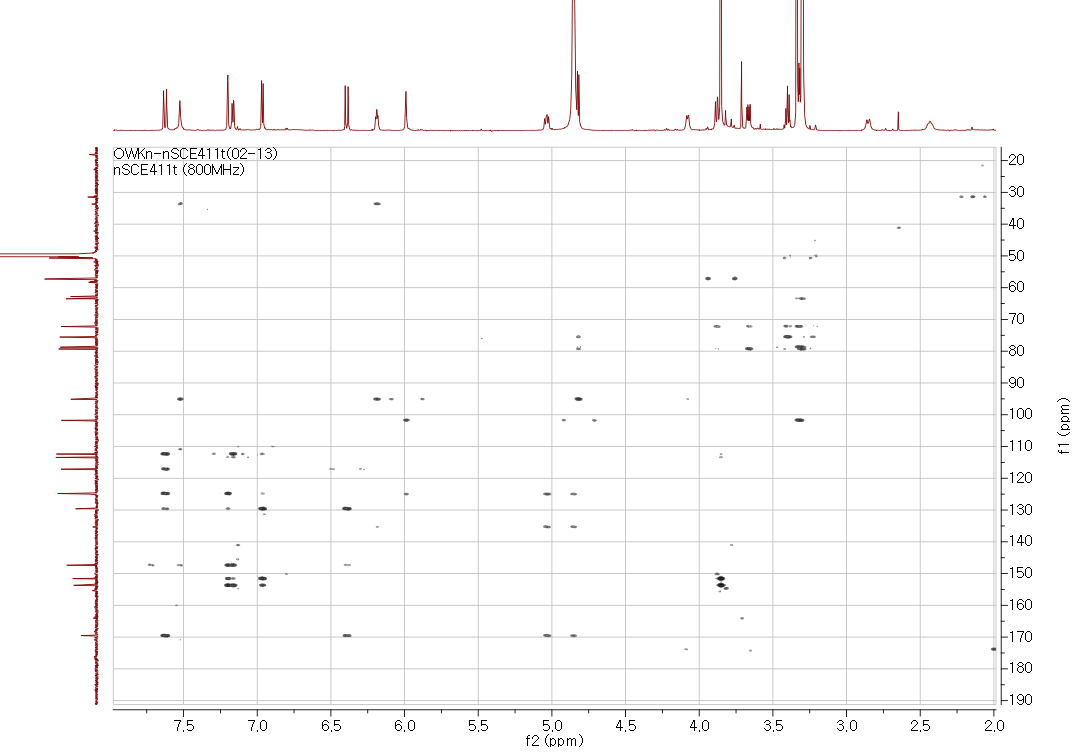
**

**Figure S18:** HSQC and HMBC NMR spectra of compound **5** (800 MHz, CD_3_OD)

**
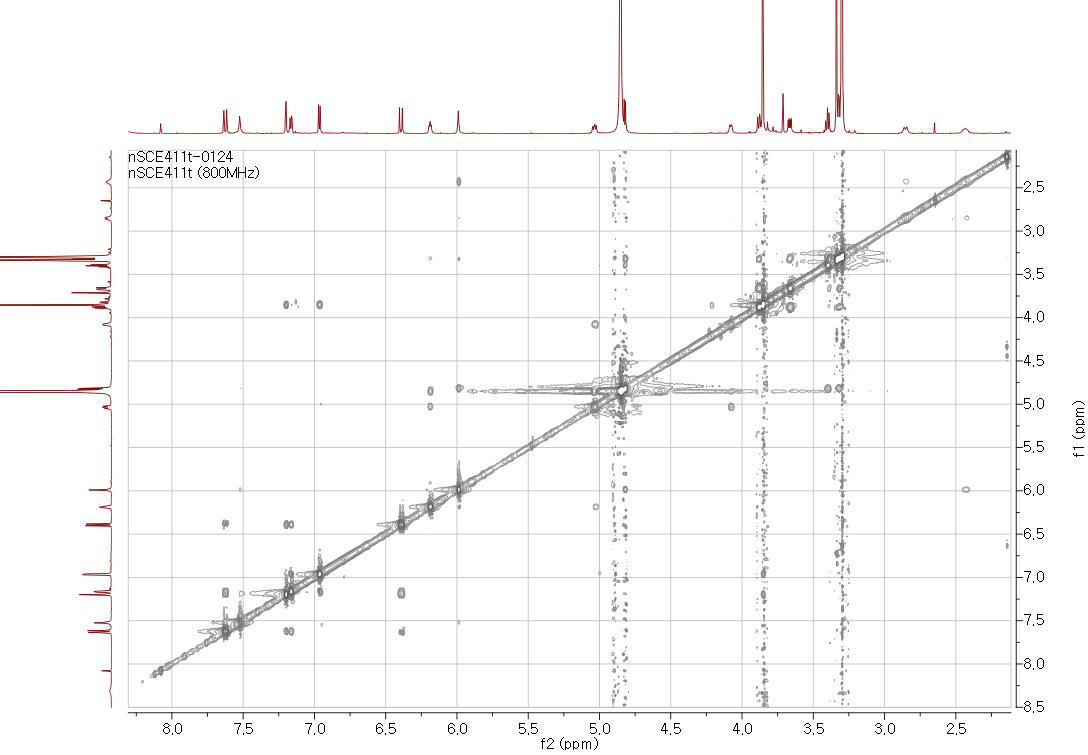
Figure S19:** ROESY spectrum of compound **5** (800 MHz, CD_3_OD)


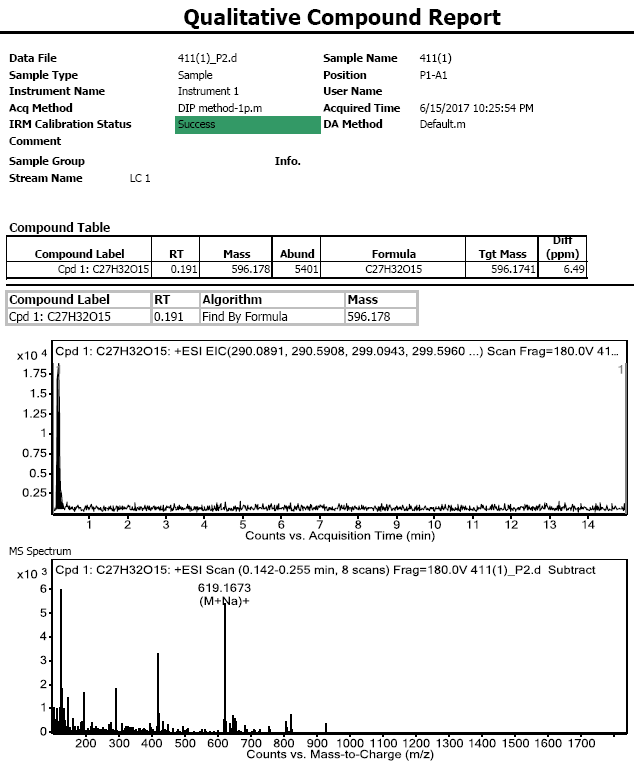


**Figure S20:** HR-ESI(+)MS of compound **5**

**
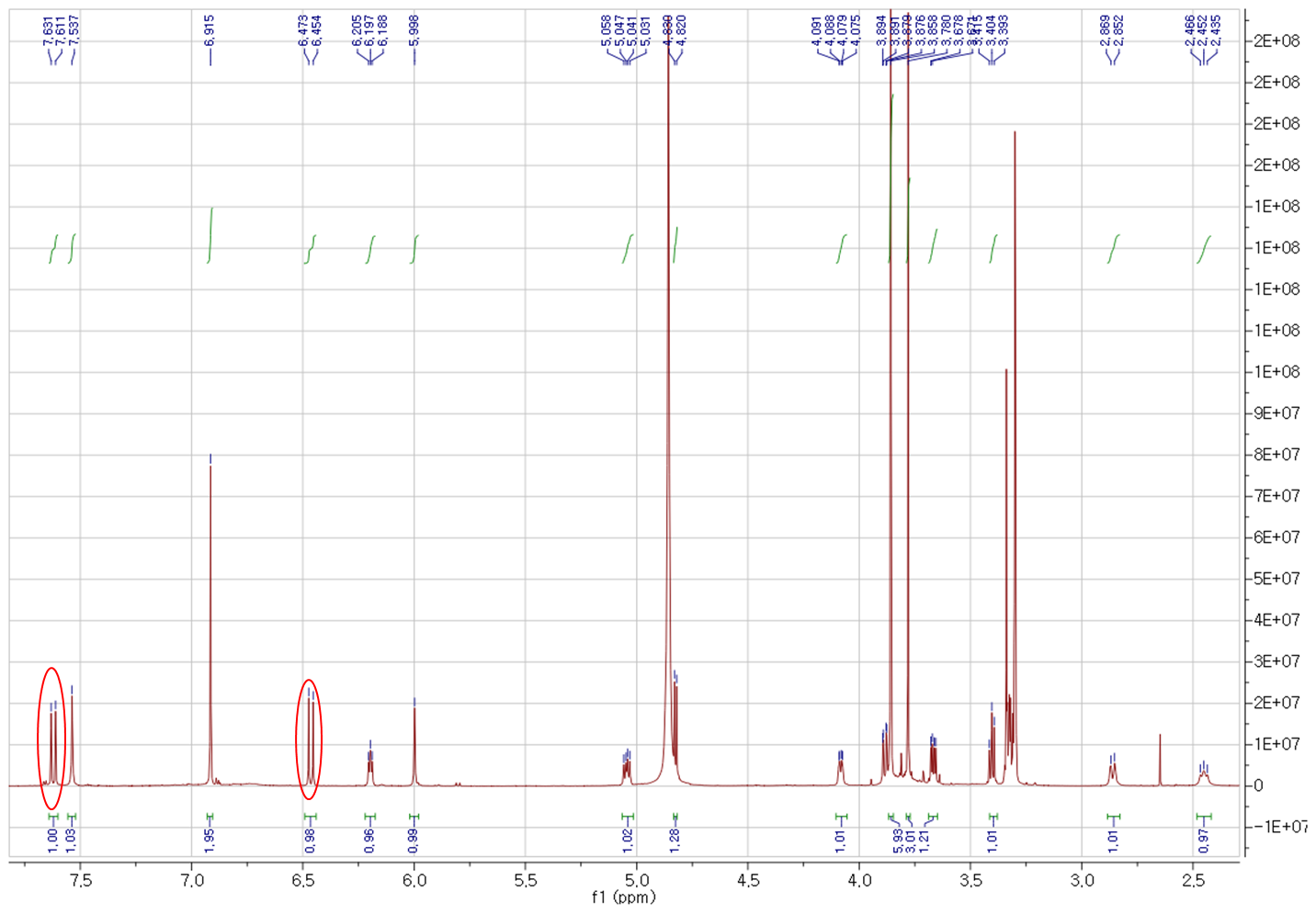
**

**
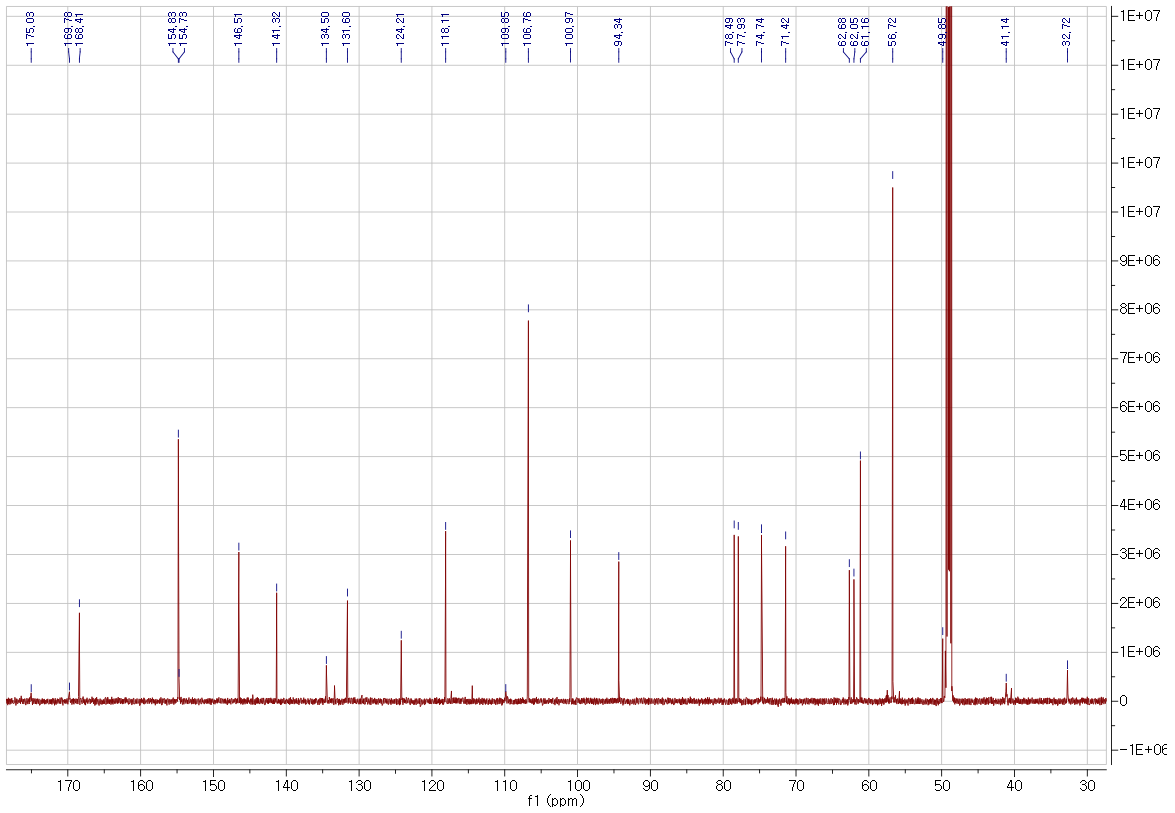
**

**Figure S21:** ^1^H and ^13^C NMR spectra of compound **6** (800, 200 MHz, CD_3_OD)


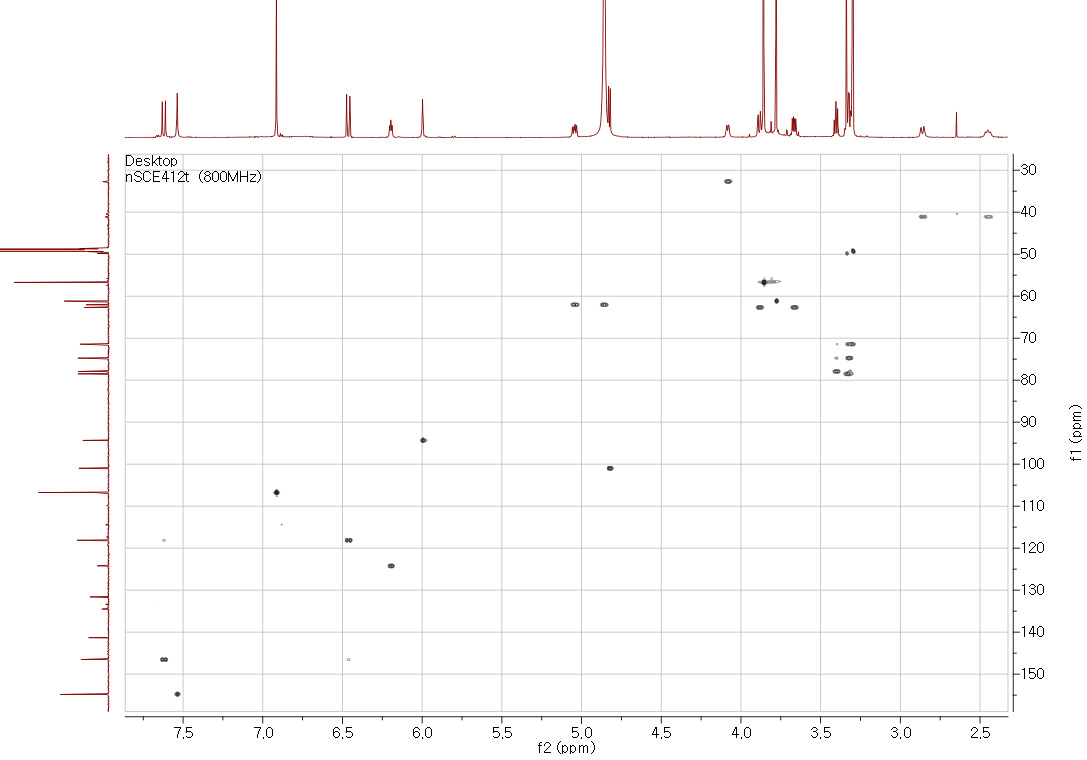


**
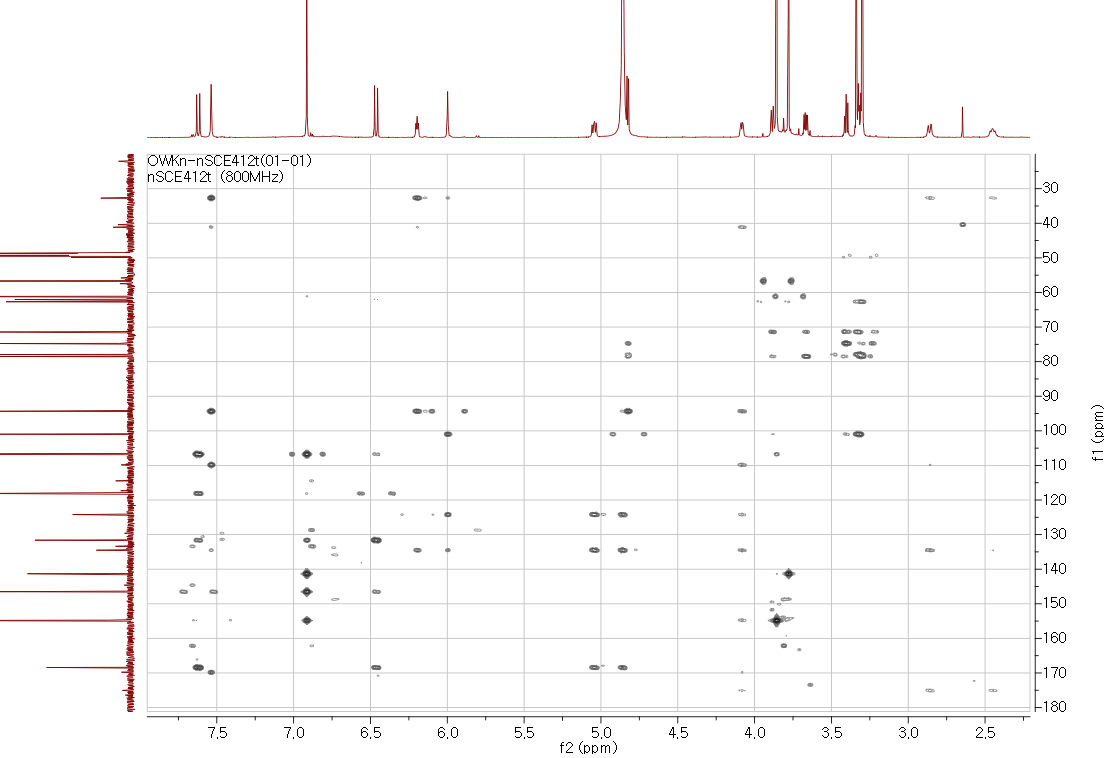
**

**Figure S22:** HSQC and HMBC NMR spectra of compound **6** (800 MHz, CD_3_OD)

**
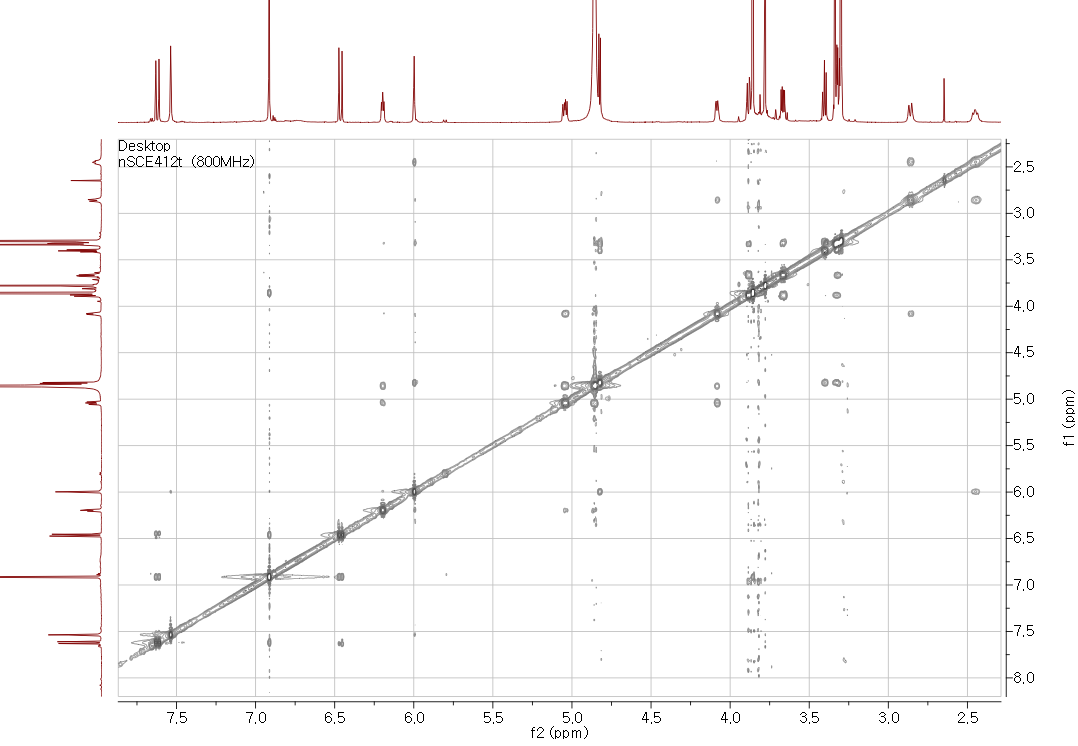
**

**Figure S23:** ROESY NMR spectrum of compound **6** (800 MHz, CD_3_OD)


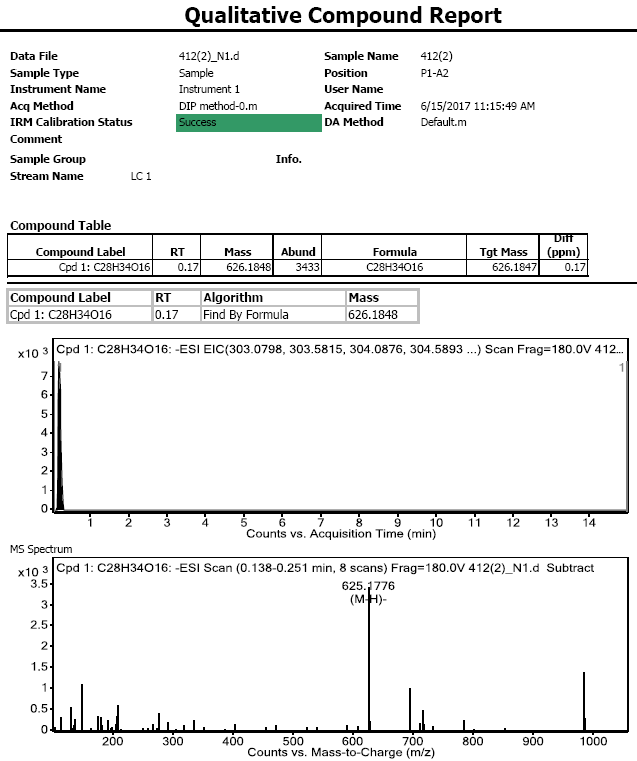


**Figure S24:** HR-ESI(-)MS of compound **6**

**
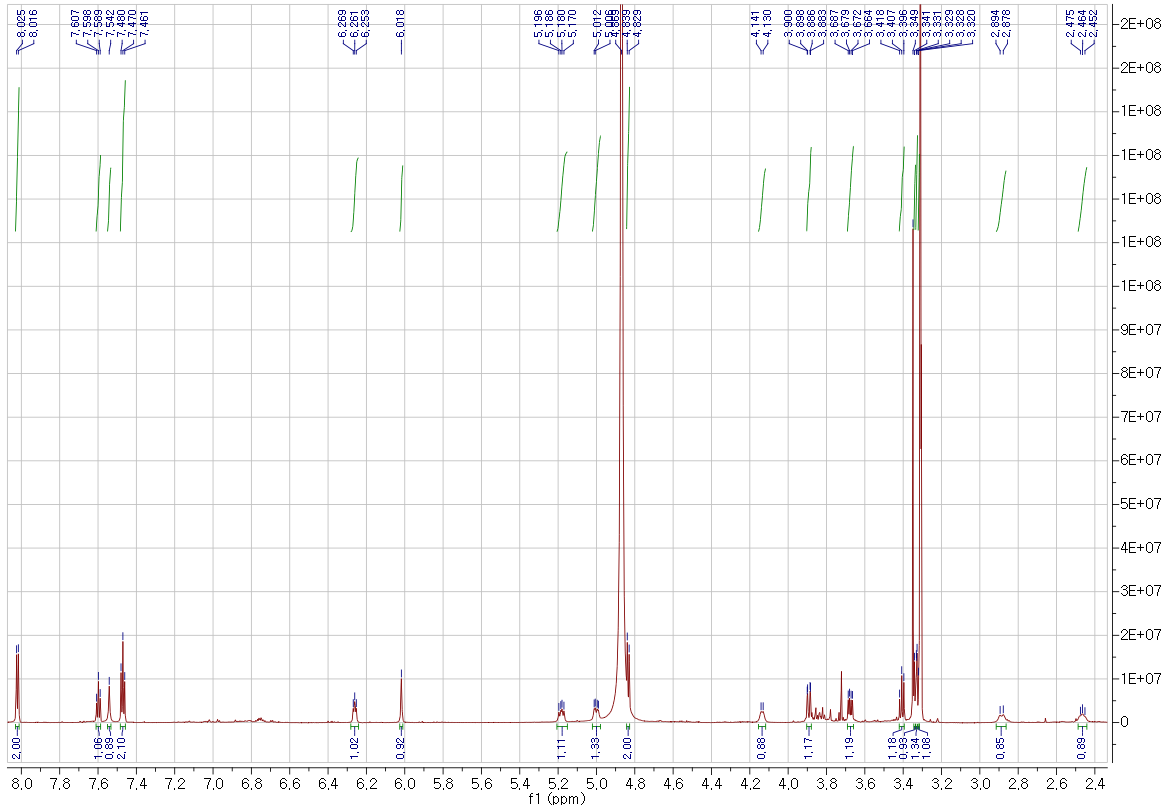
**

**
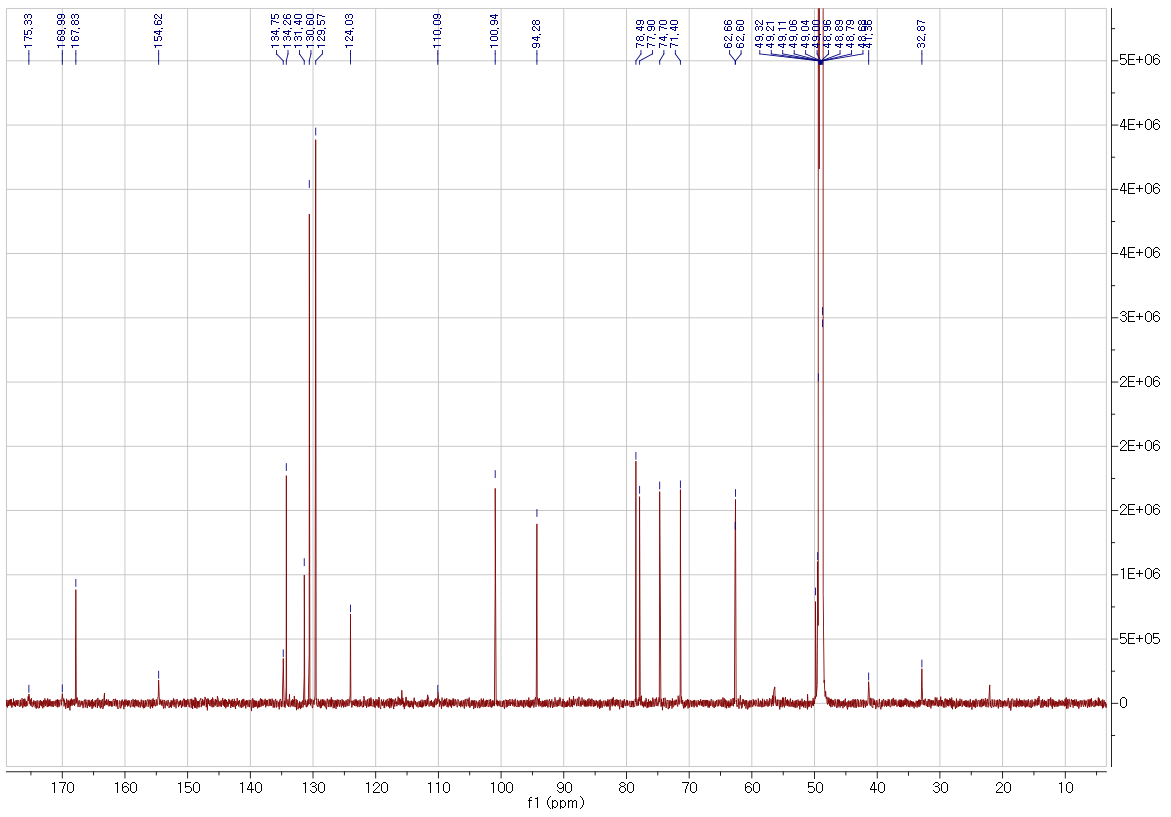
**

**Figure S25:** ^1^H and ^13^C NMR spectra of compound **8** (800, 200 MHz, CD_3_OD)

**
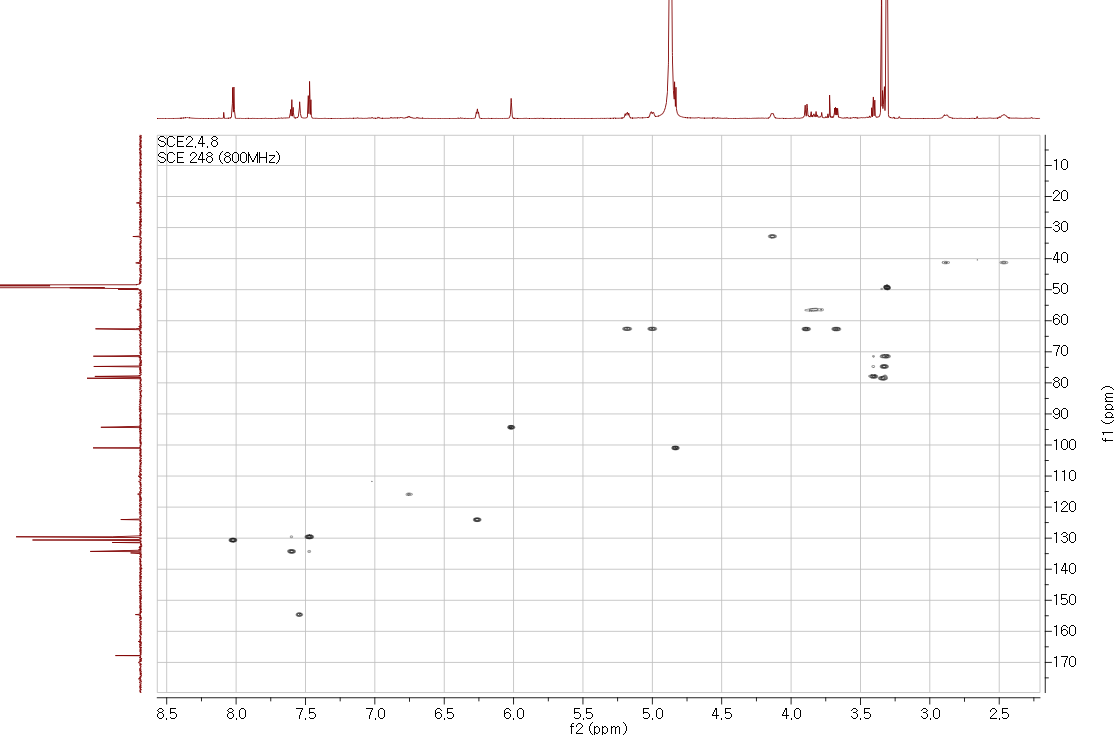
**

**
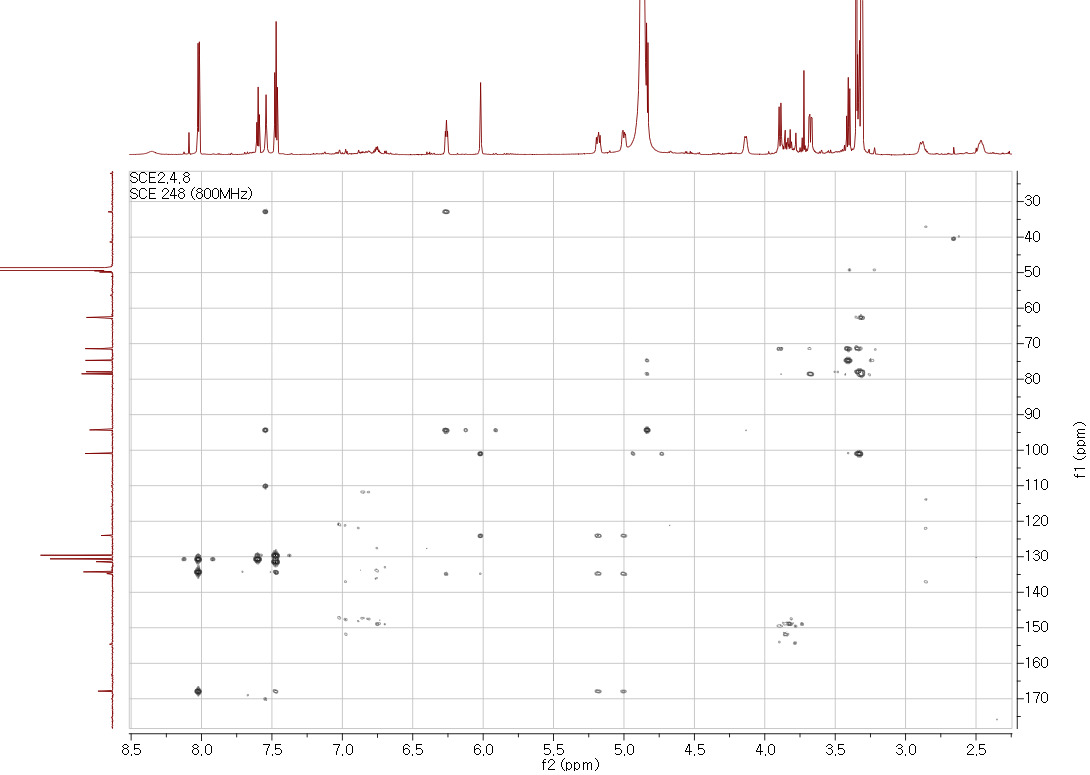
**

**Figure S26:** HSQC and HMBC NMR spectra of compound **8** (800 MHz, CD_3_OD)

**
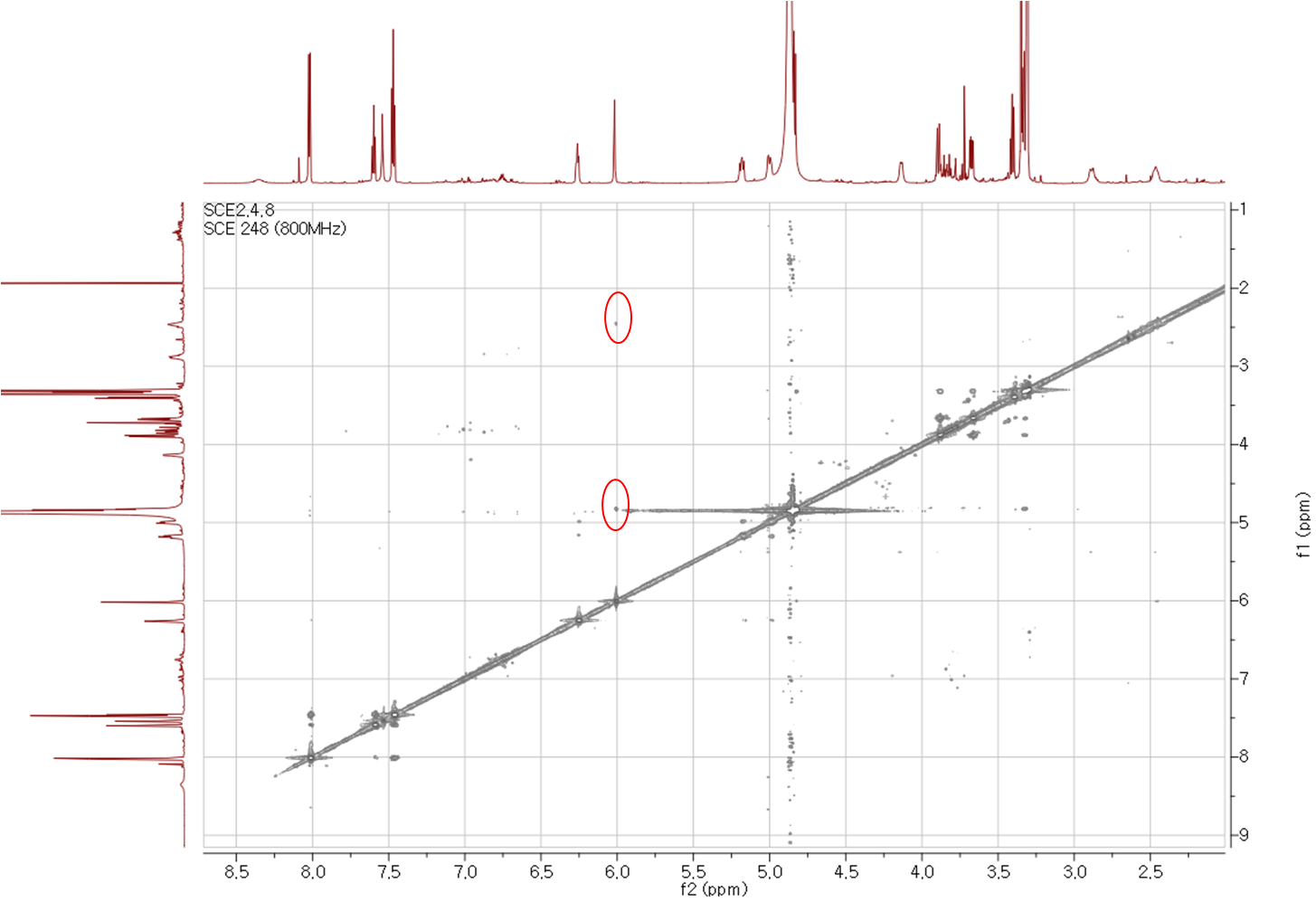
**


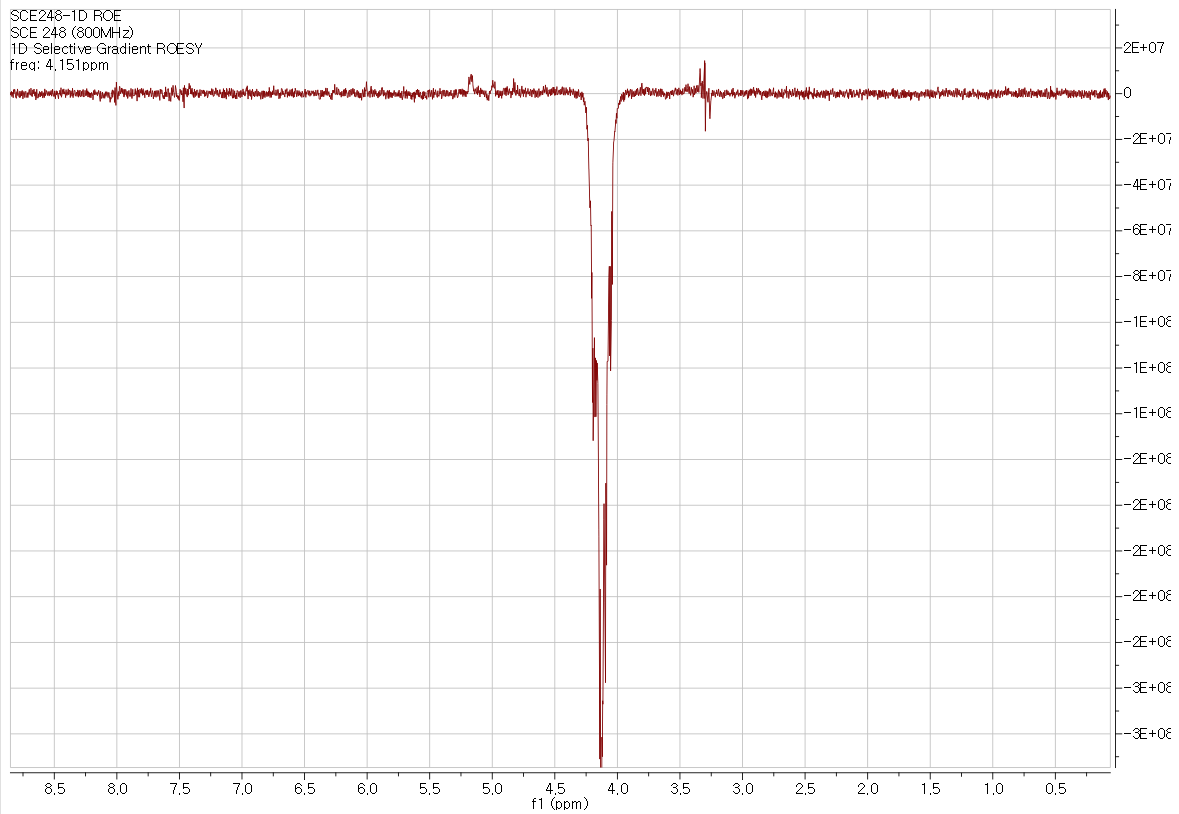


**Figure S27:** NOESY and 1D**-**ROE spectra of compound **8** (800 MHz, CD_3_OD)


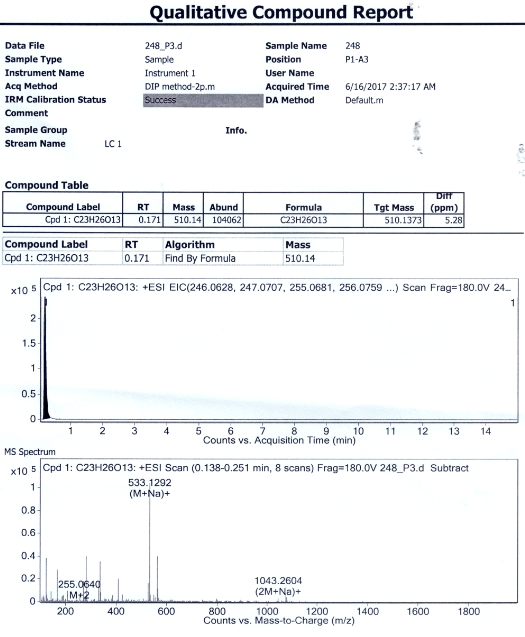


**Figure S28:** HR-ESI(+)MS of compound **8**

**
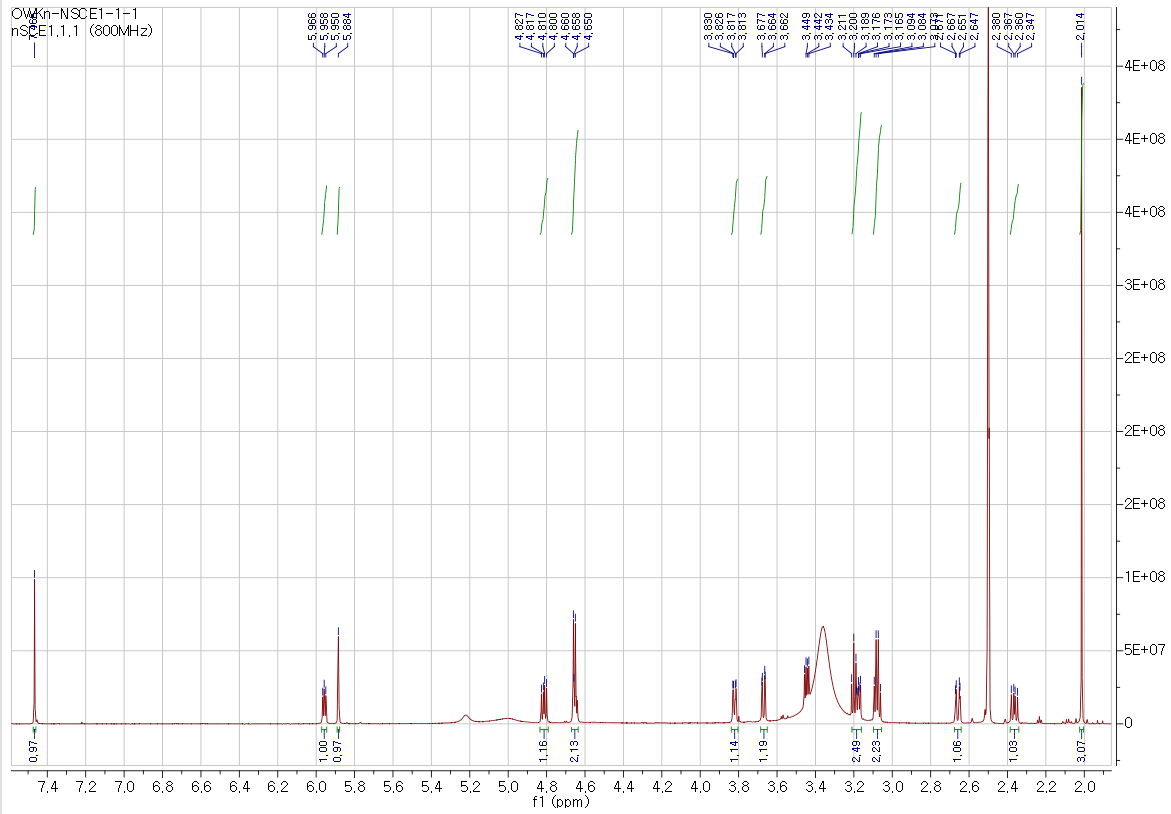
**

**
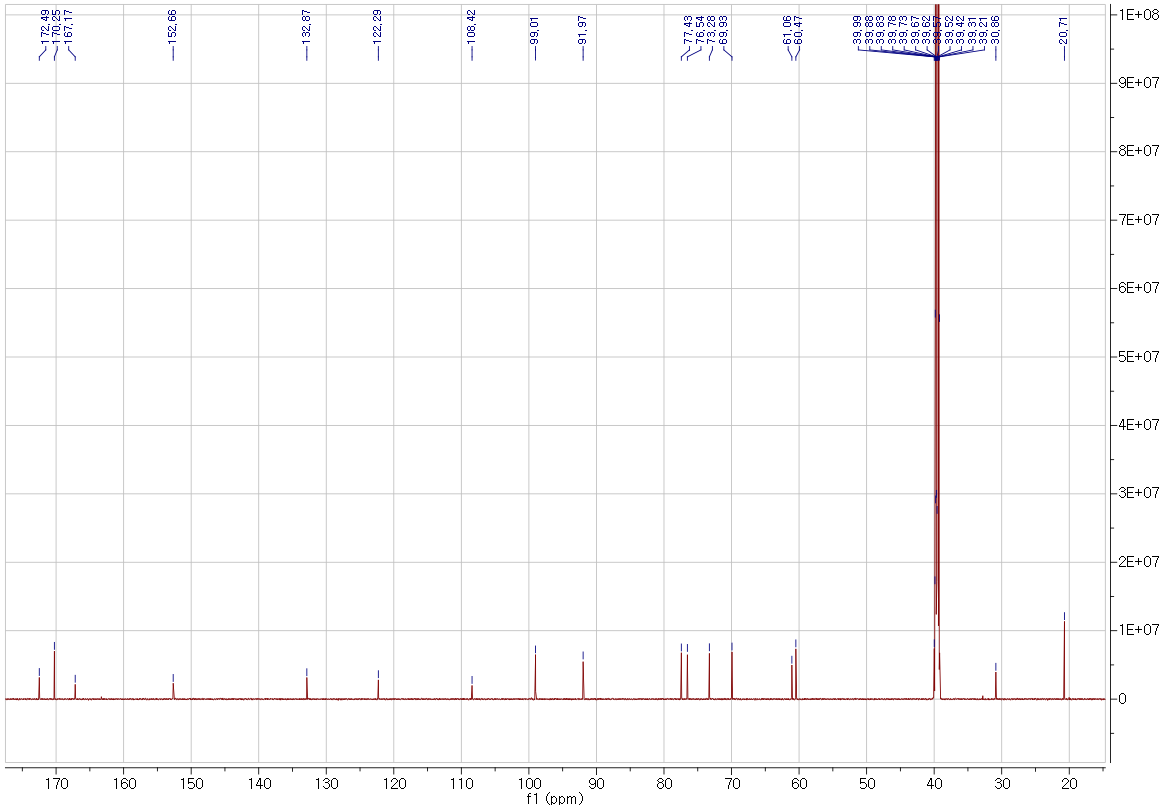
**

**Figure S29:** ^1^H and ^13^C NMR spectra of compound **9** (800, 200 MHz, DMSO-*d*_6_)

**
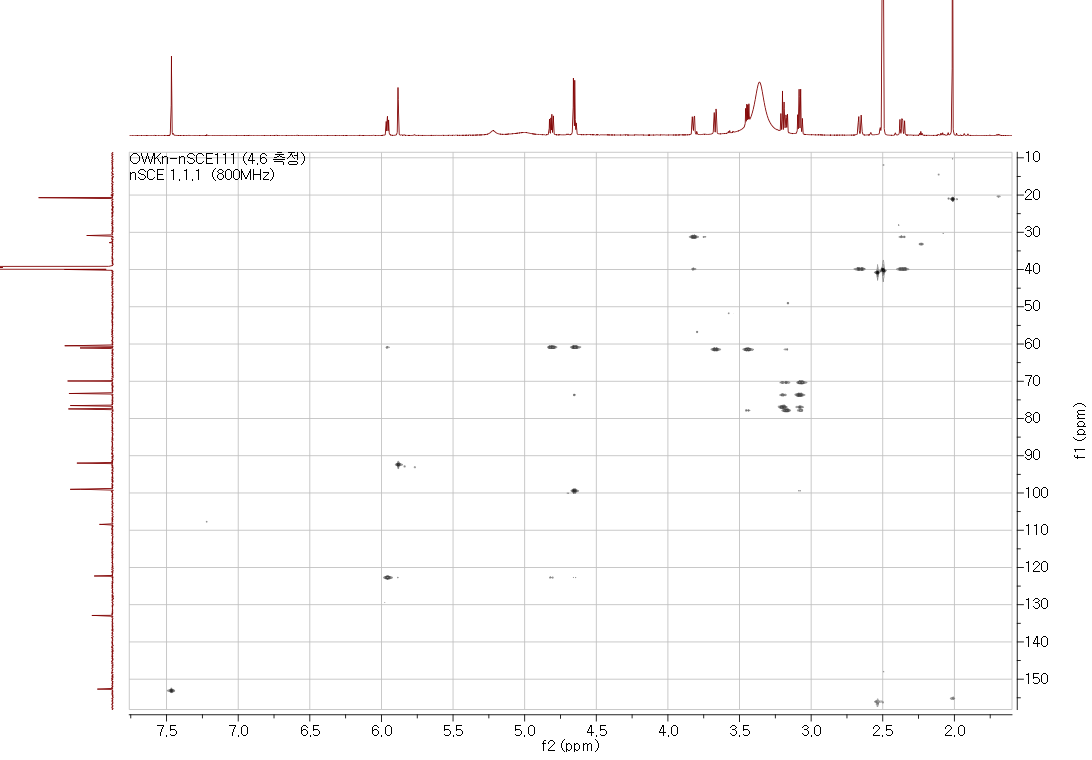
**

**
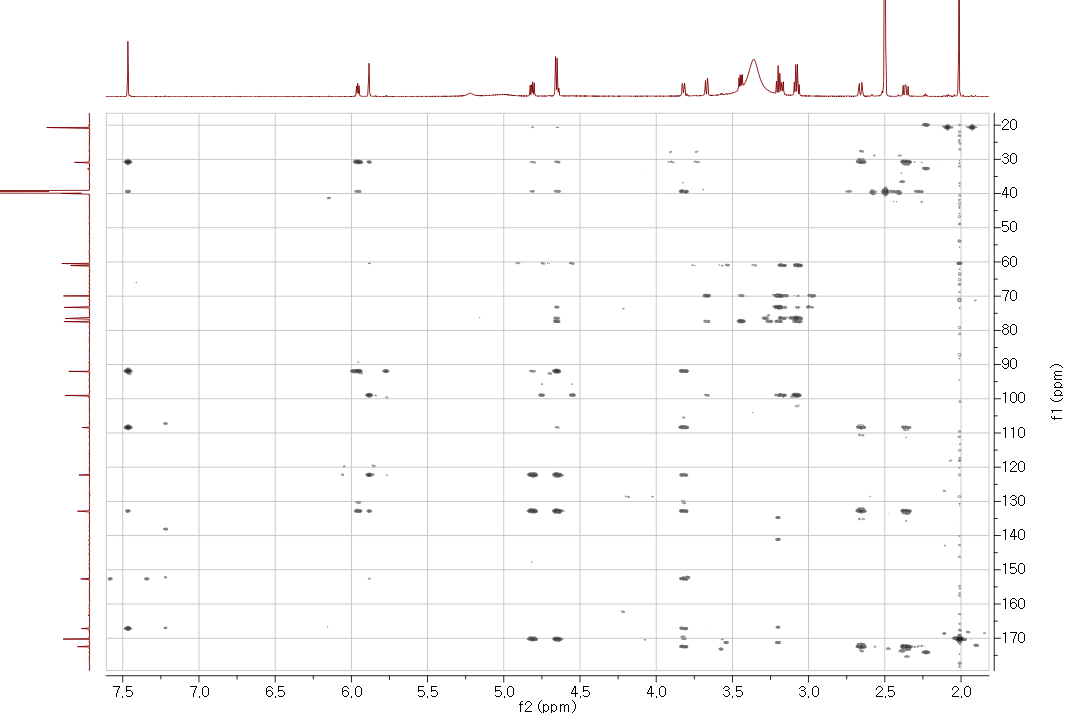
**

**Figure S30:** HSQC and HMBC NMR spectra of compound **9** (800 MHz, DMSO-*d_6_*)

**
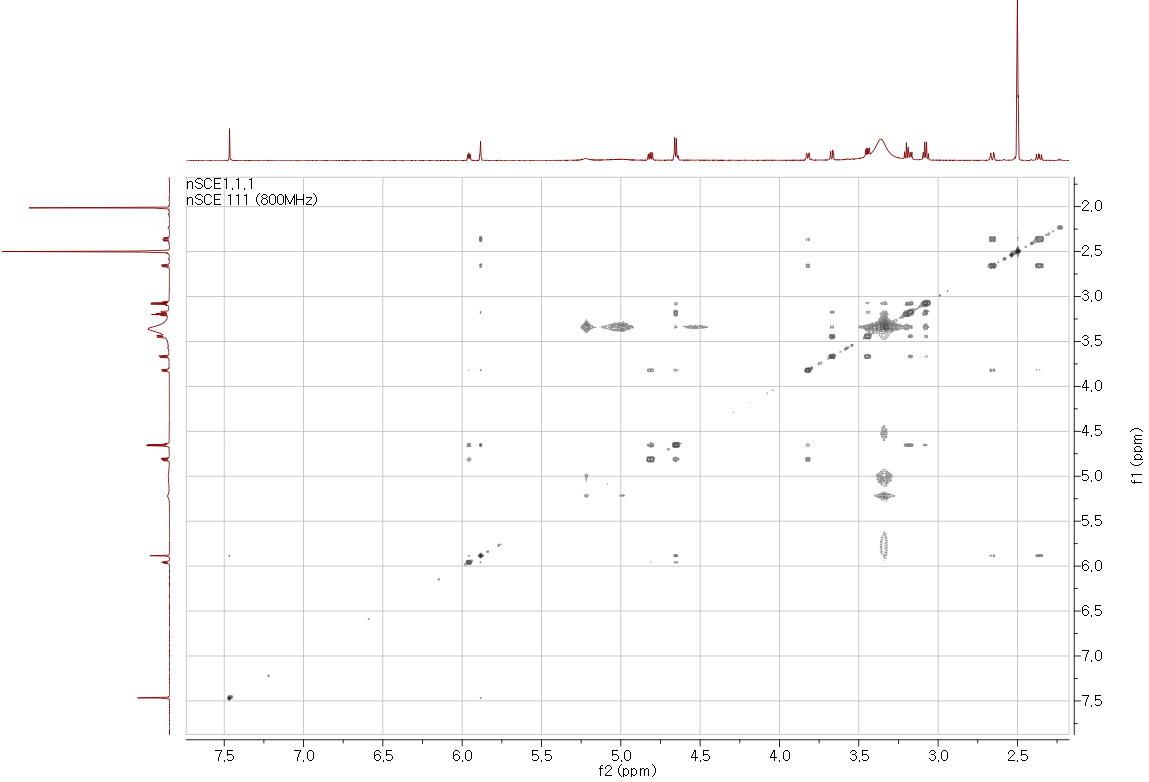
**

**Figure S31:** NOESY NMR spectrum of compound **9** (800 MHz, DMSO-*d*_6_)


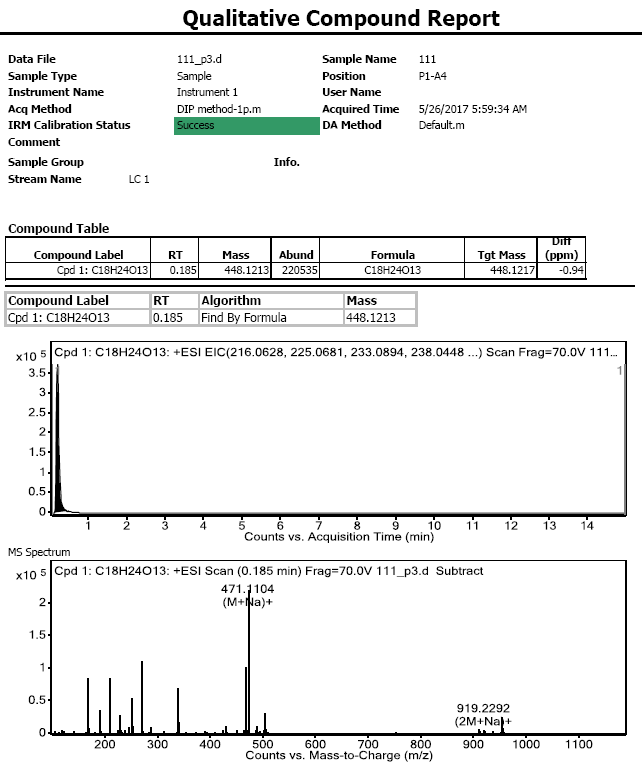


**Figure S32:** HR-ESI(+)MS of compound **9**


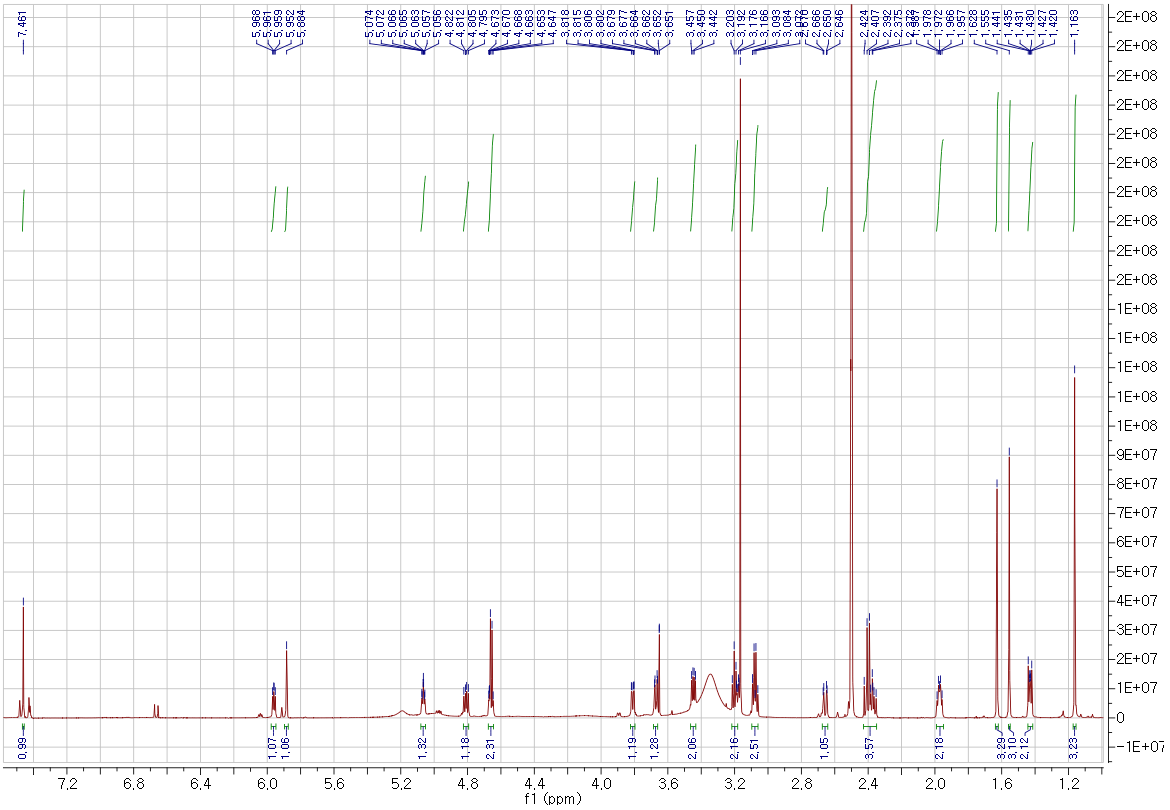


**
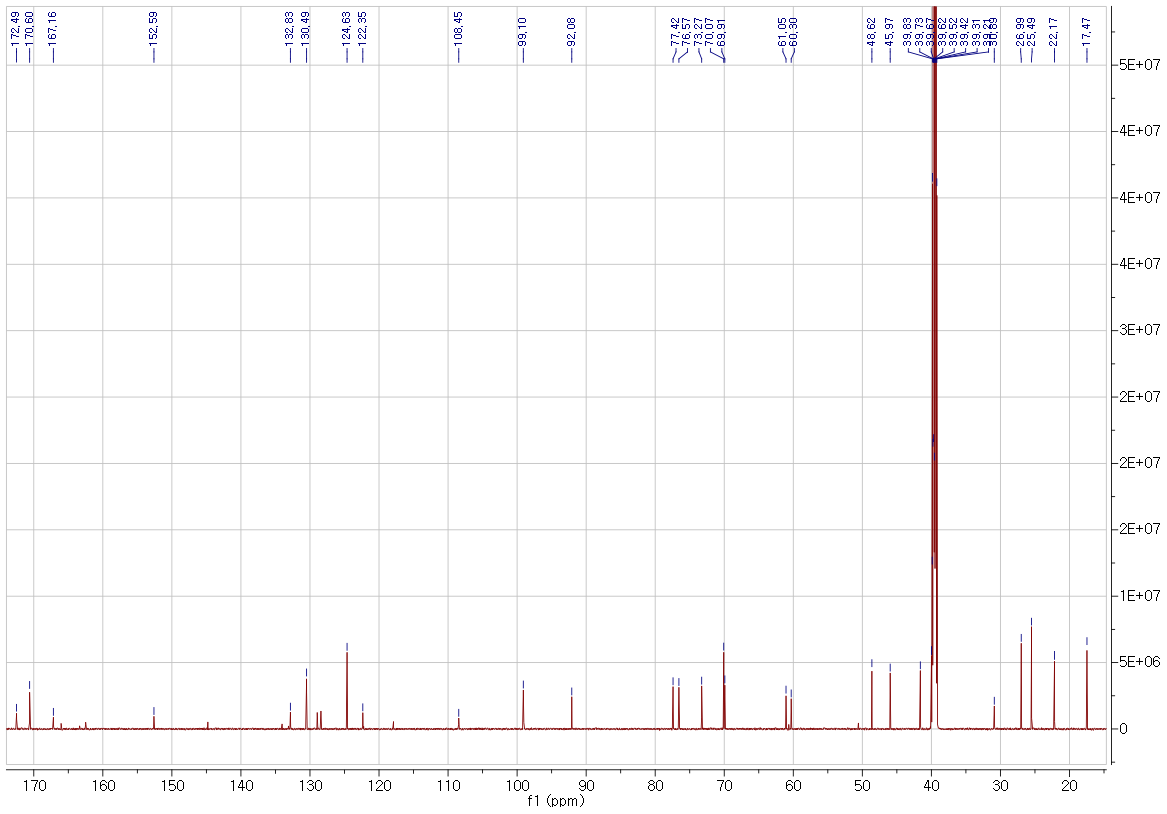
**

**Figure S33:** ^1^H and ^13^C NMR spectra of compound **10** (800, 200 MHz, DMSO-*d*_6_)

**
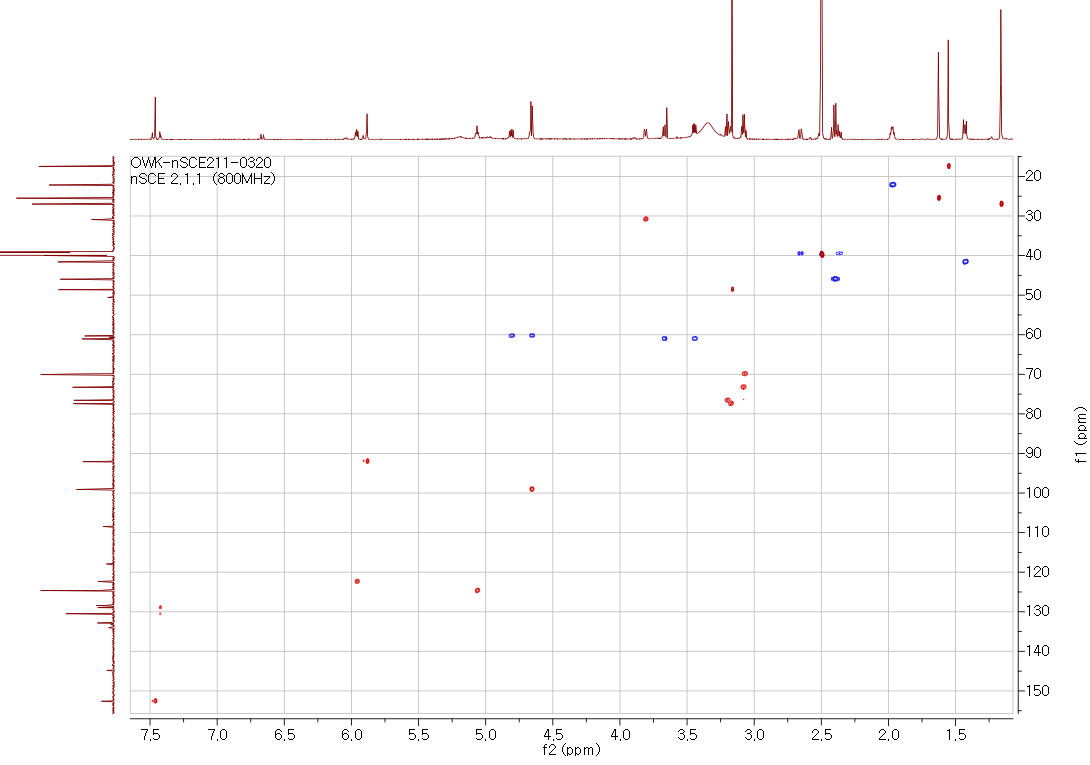
**

**
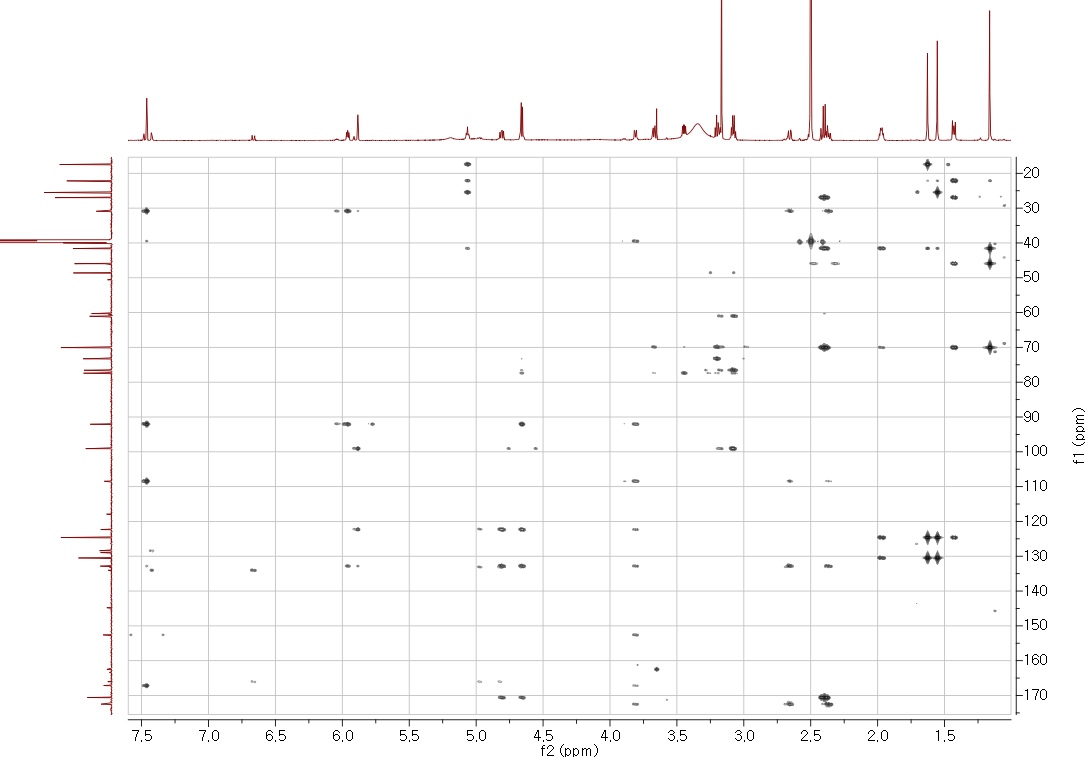
**

**Figure S34:** HSQC and HMBC NMR spectra of compound **10** (800 MHz, DMSO-*d_6_*)


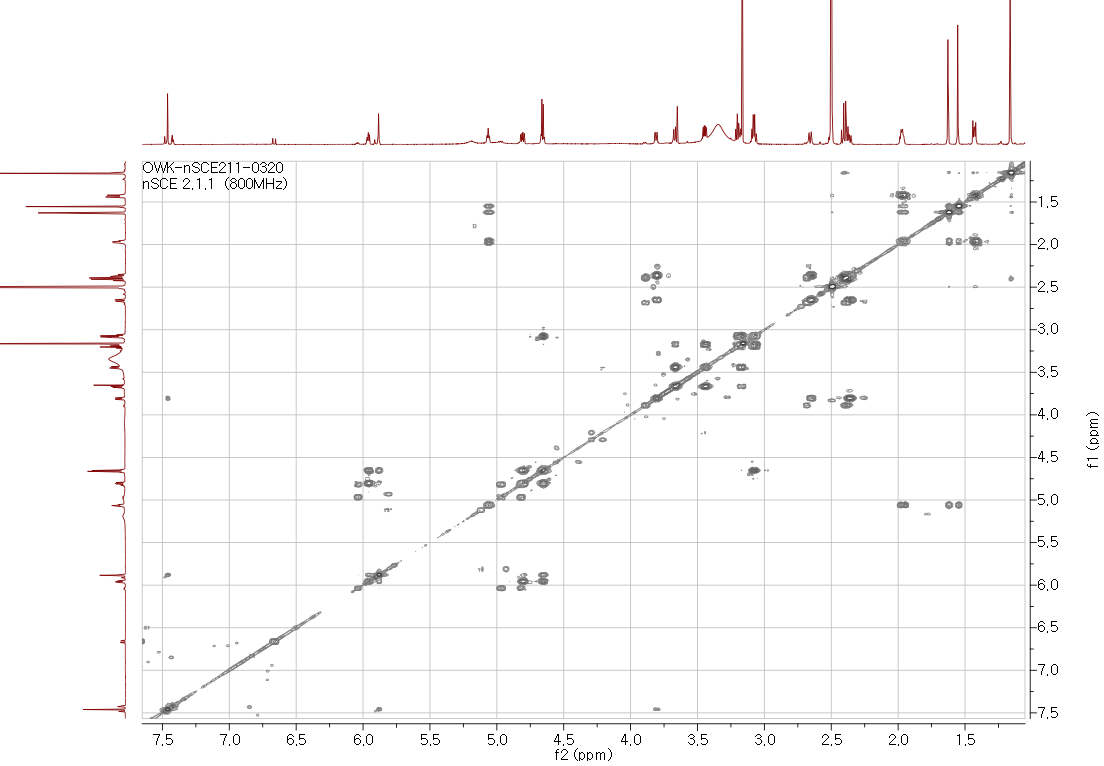


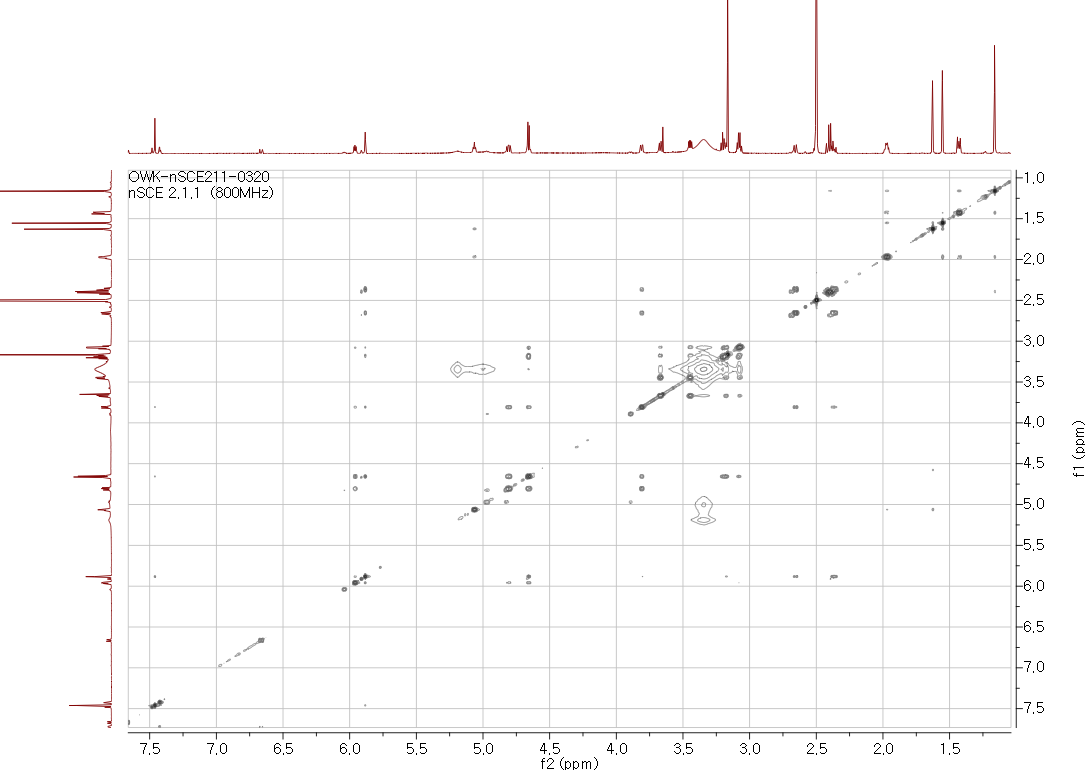


**Figure S35:** COSY and ROESY NMR spectra of compound **10** (800 MHz, DMSO-*d*_6_)


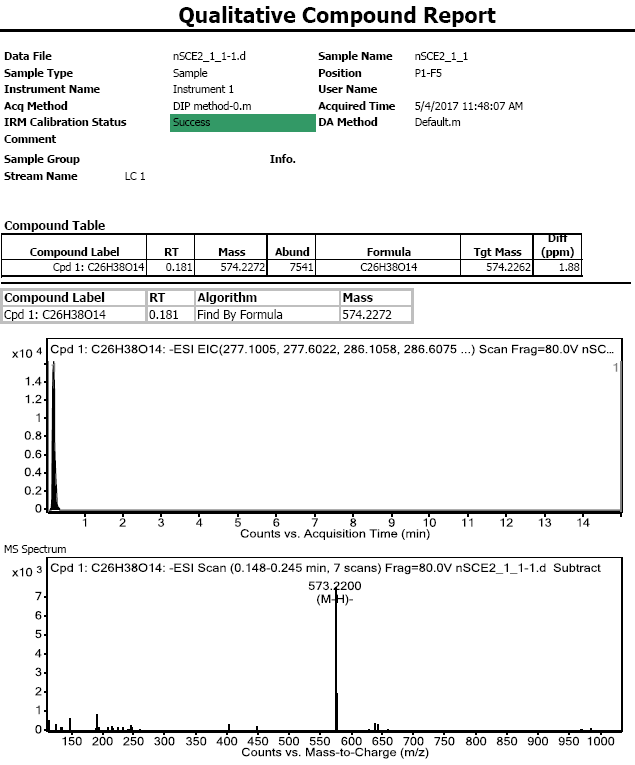


**Figure S36:** HR-ESI(-)MS of compound **10**


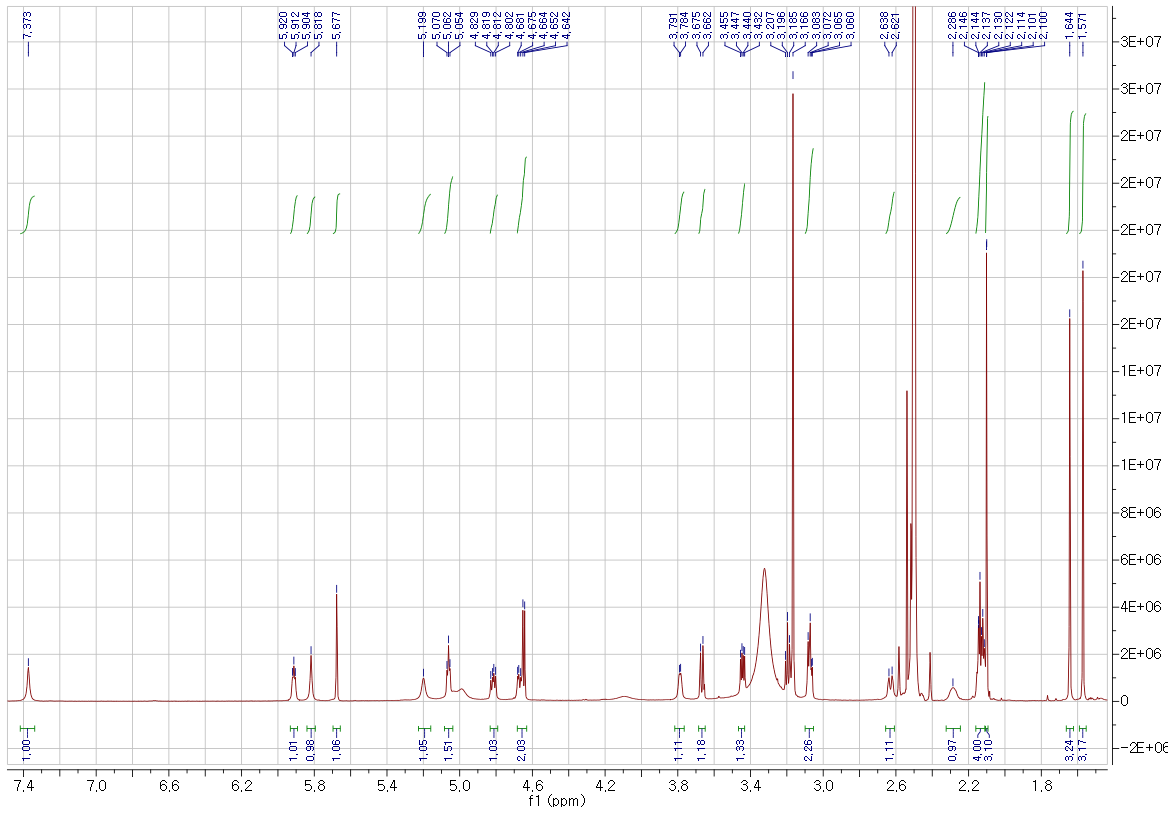


**
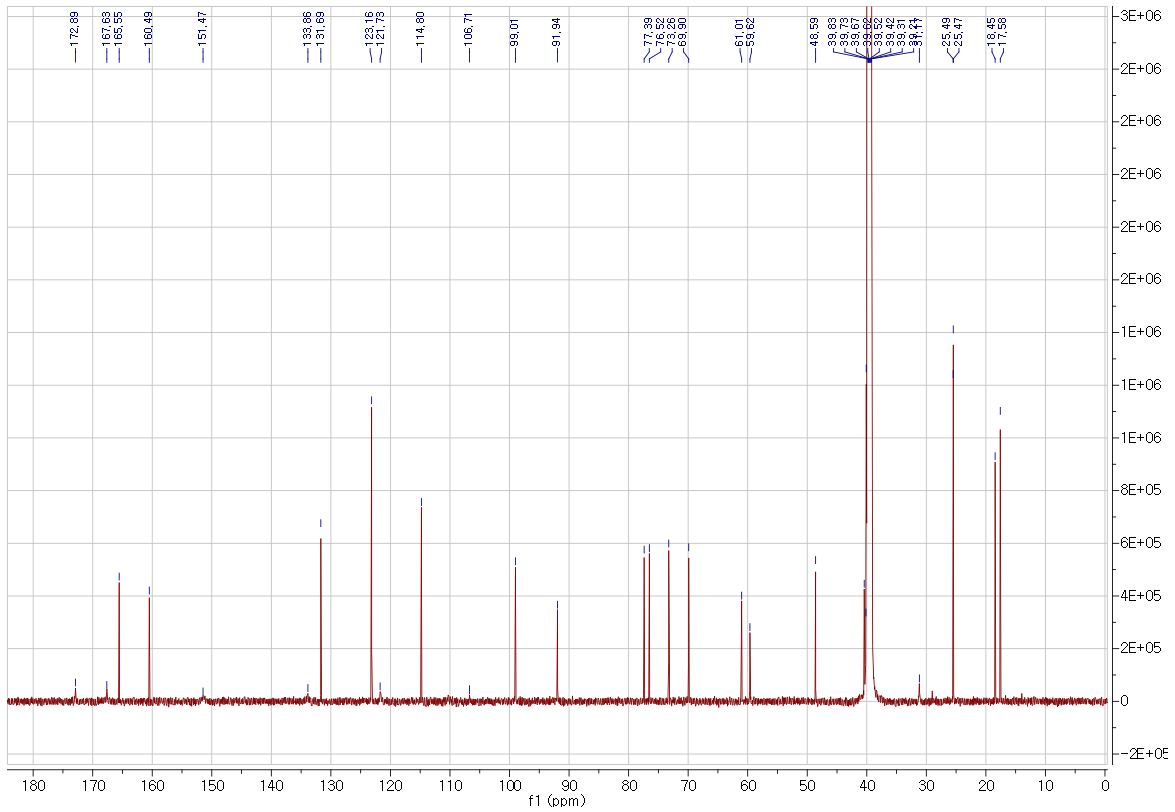
**

**Figure S37:** ^1^H and ^13^C NMR spectra of compound **11** (800, 200 MHz, DMSO-*d*_6_)

**
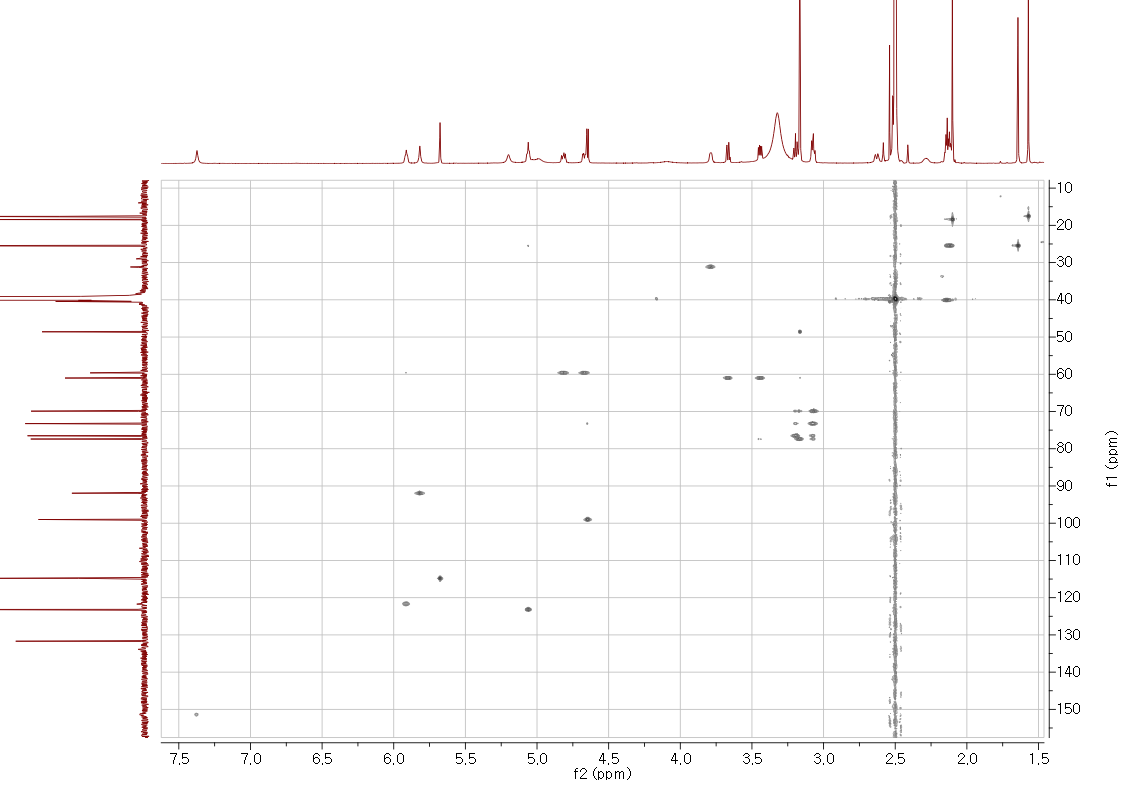
**

**
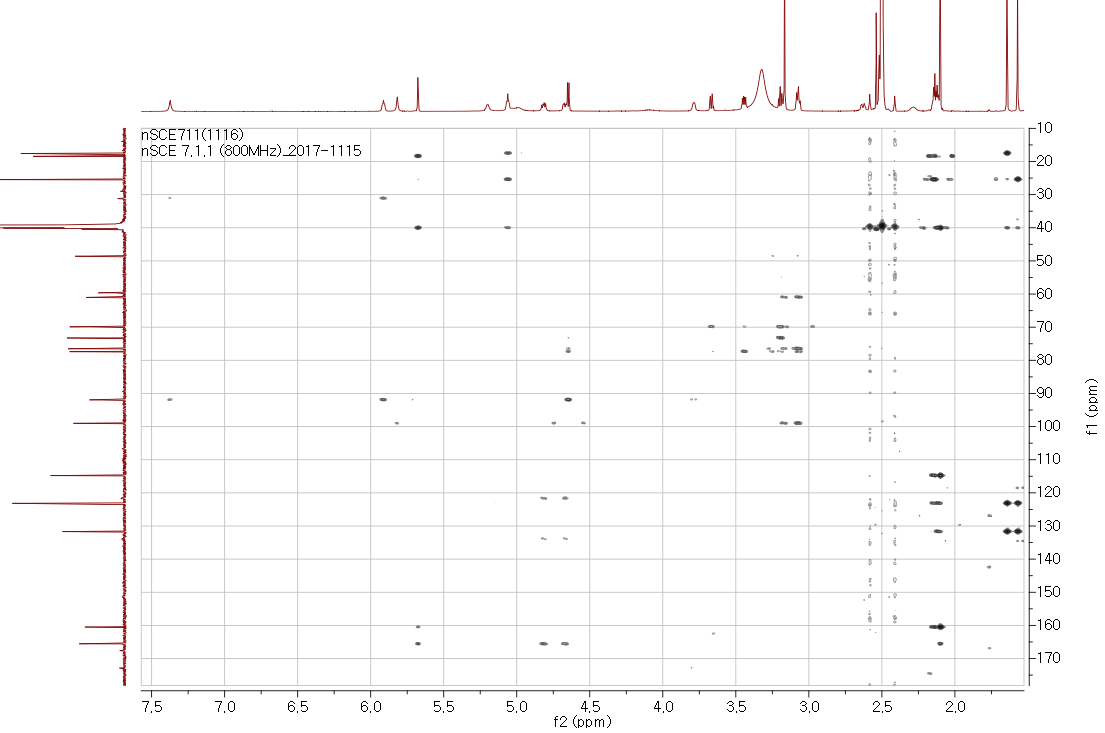
**

**Figure S38:** HSQC and HMBC NMR spectra of compound **11** (800 MHz, DMSO-*d_6_*)


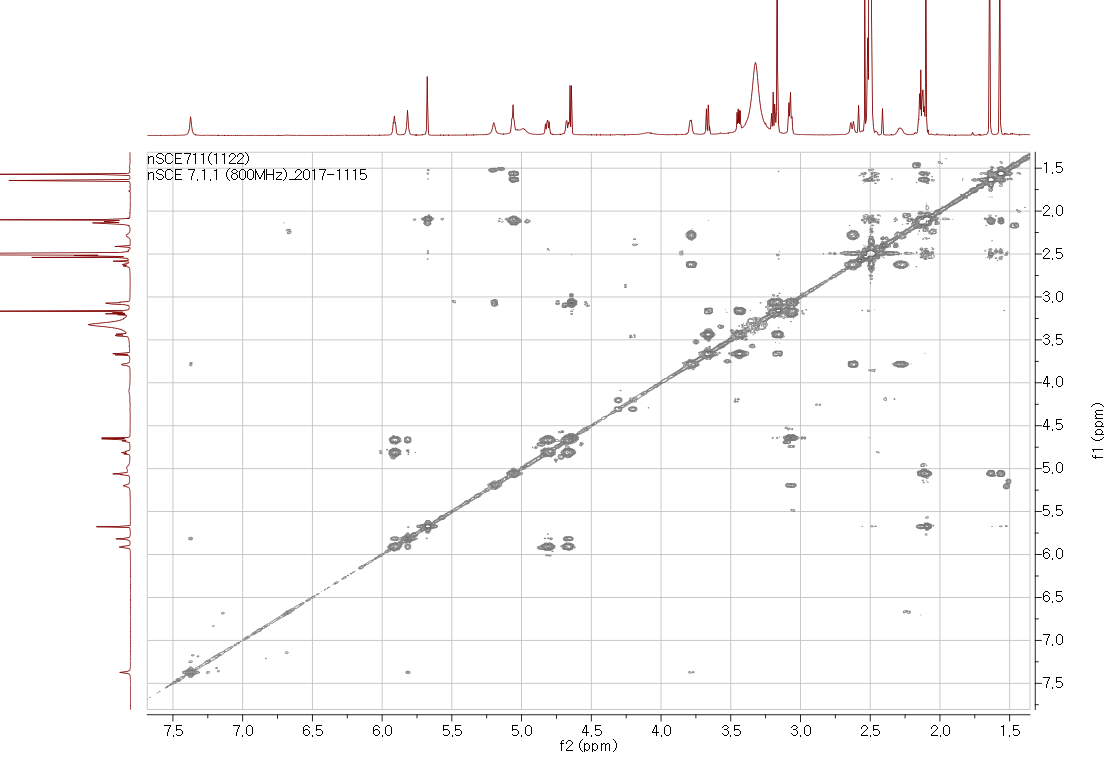


**
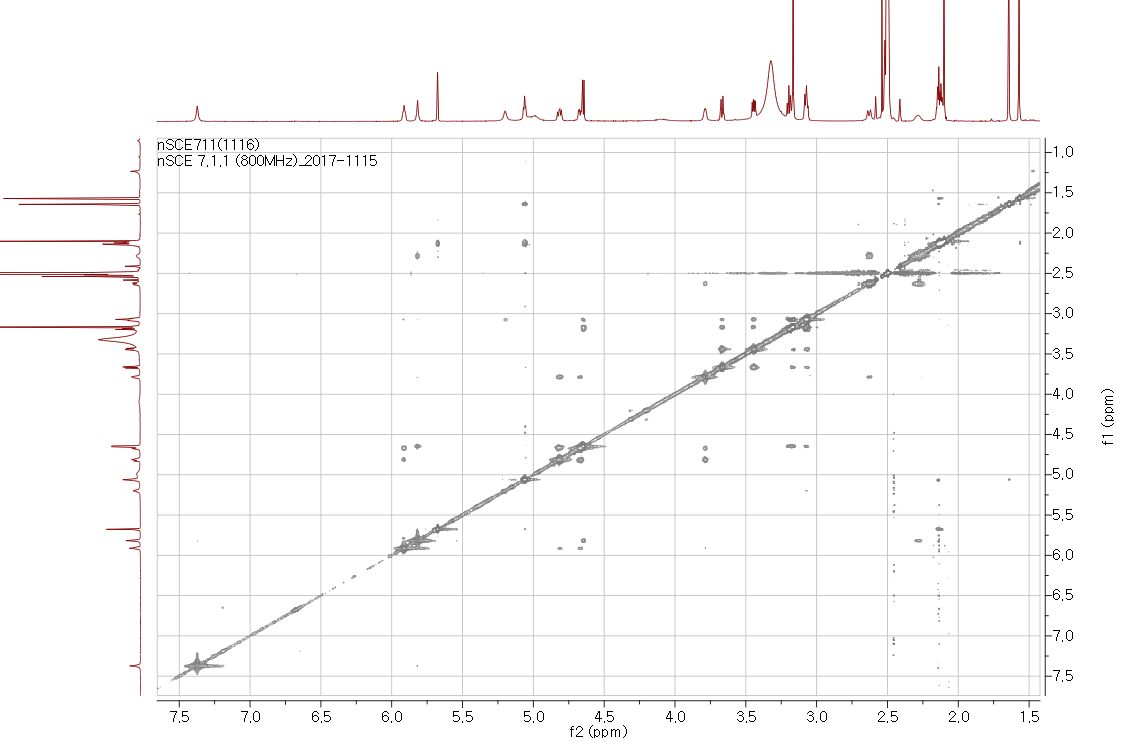
**

**Figure S39:** COSY and ROESY NMR spectra of compound **11** (800 MHz, DMSO-*d*_6_)

**
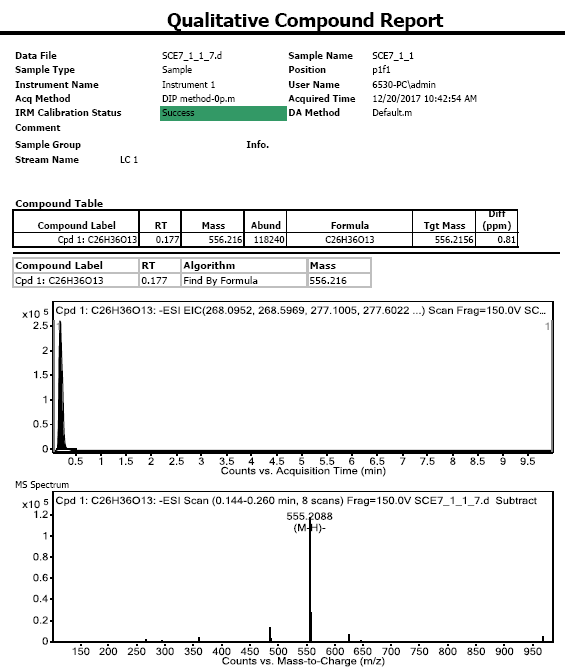
**

**Figure S40:** HR-ESI(-)MS of compound **11**

**
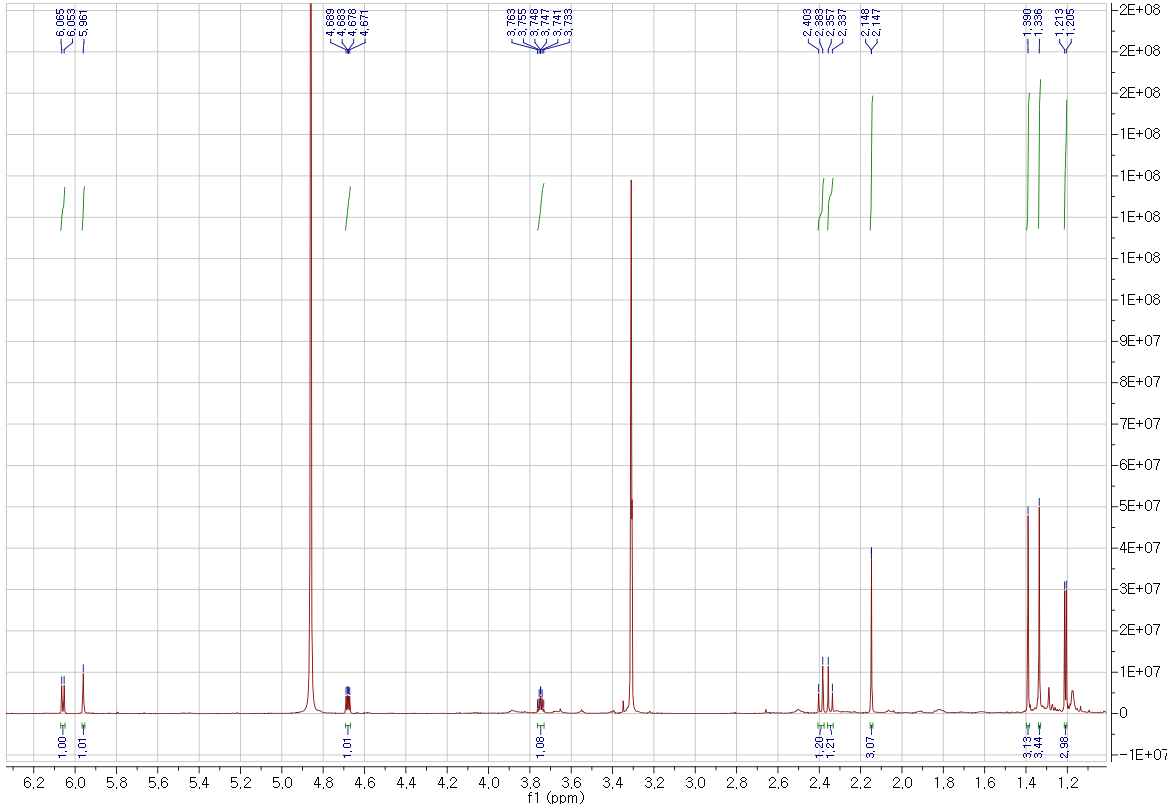
**

**
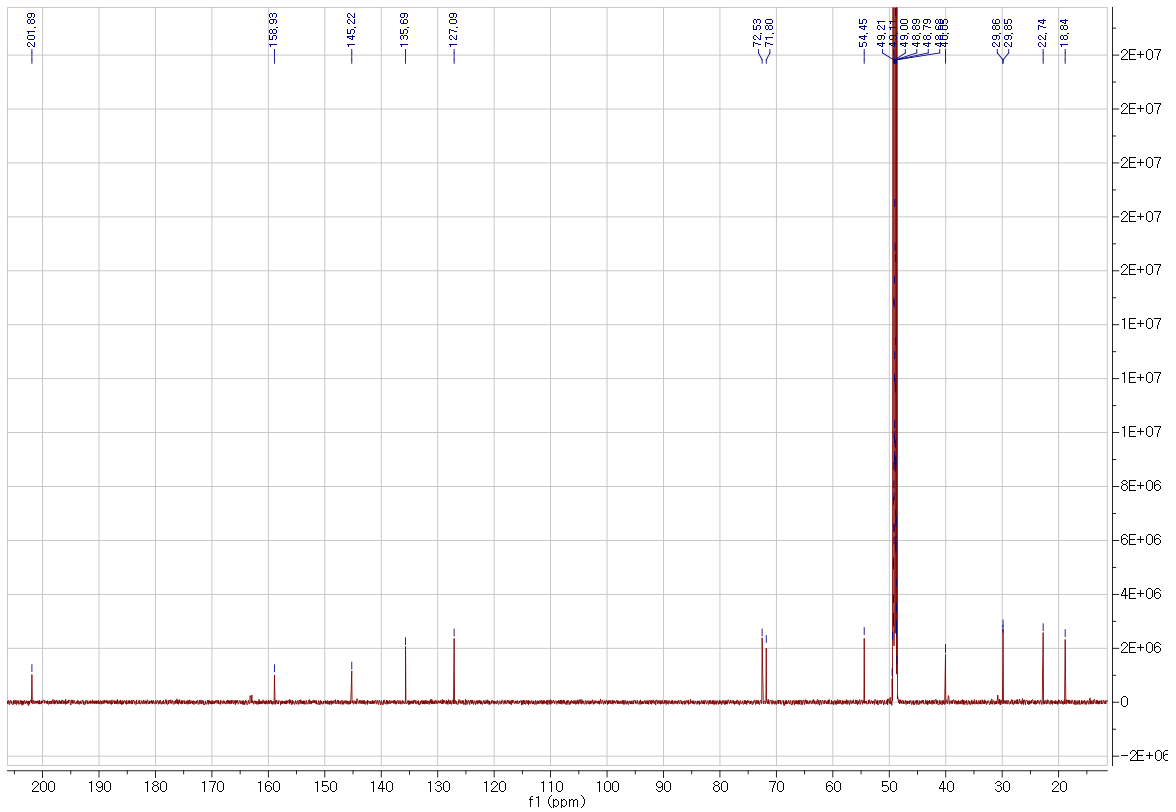
**

**Figure S41:** ^1^H and ^13^C NMR spectra of compound **12** (800, 200 MHz, CD_3_OD)

**
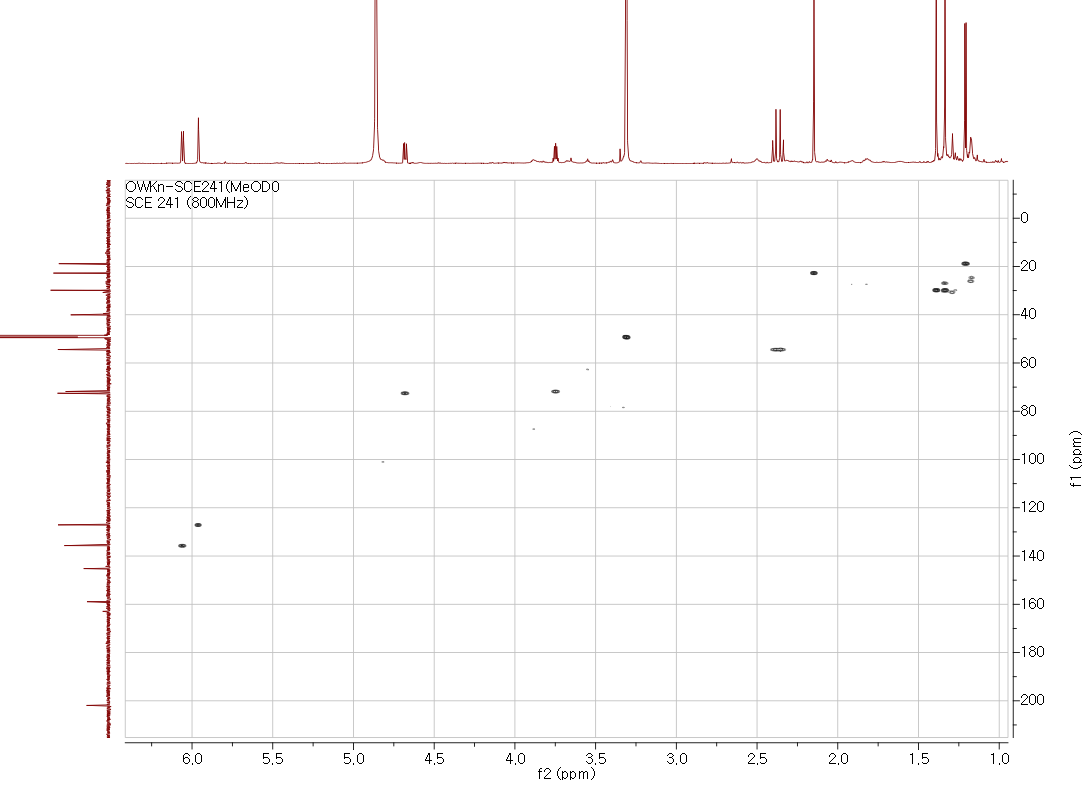
**

**
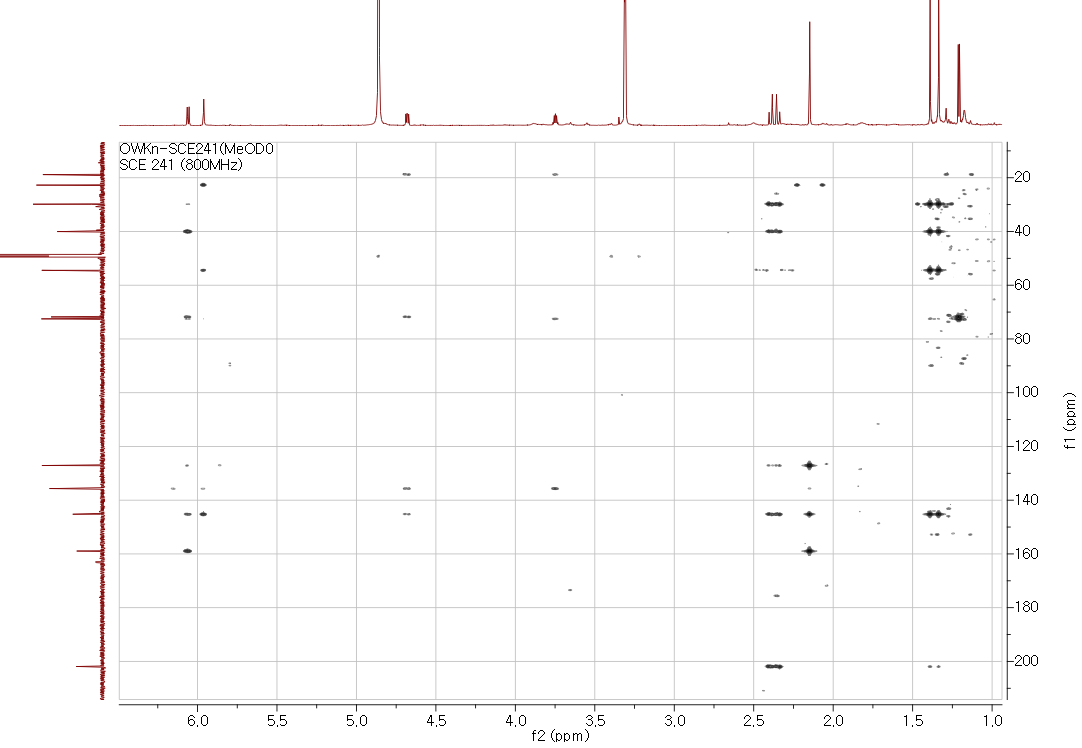
**

**Figure S42:** HSQC and HMBC NMR spectra of compound **12** (800 MHz, CD_3_OD)

**
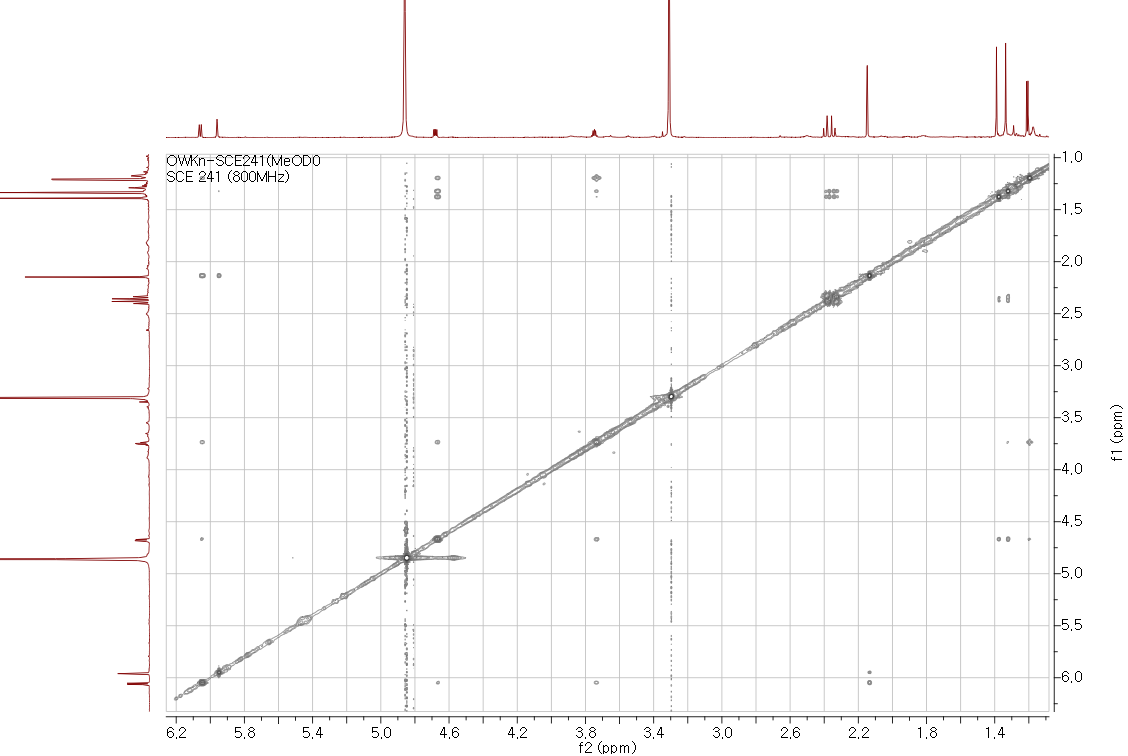
**

**Figure S43:** ROESY NMR spectrum of compound **12** (800 MHz, CD_3_OD)

**
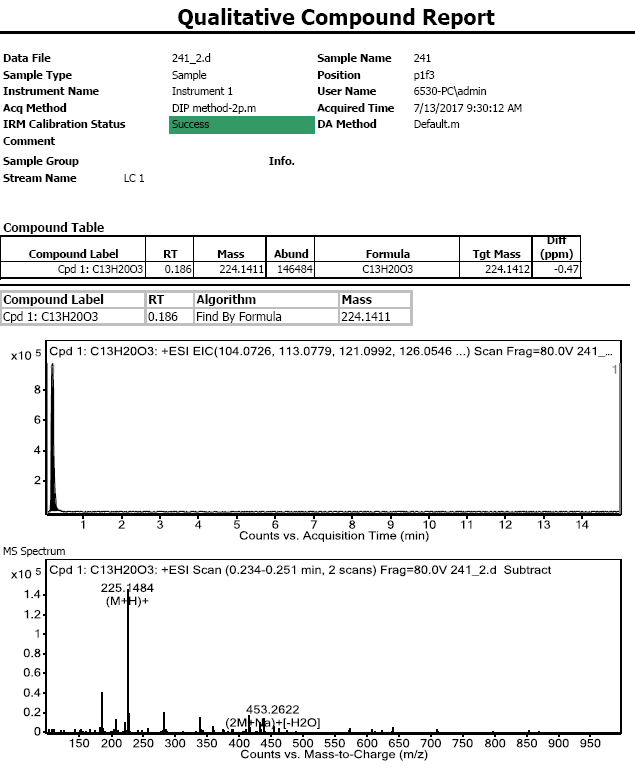
**

**Figure S44:** HR-ESI(+)MS of compound **12**

**
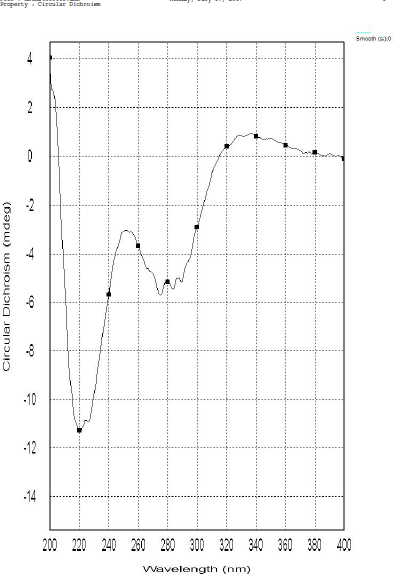
**

**Figure S45:** Experimental CD spectrum of compound **12** (MeOH)

**
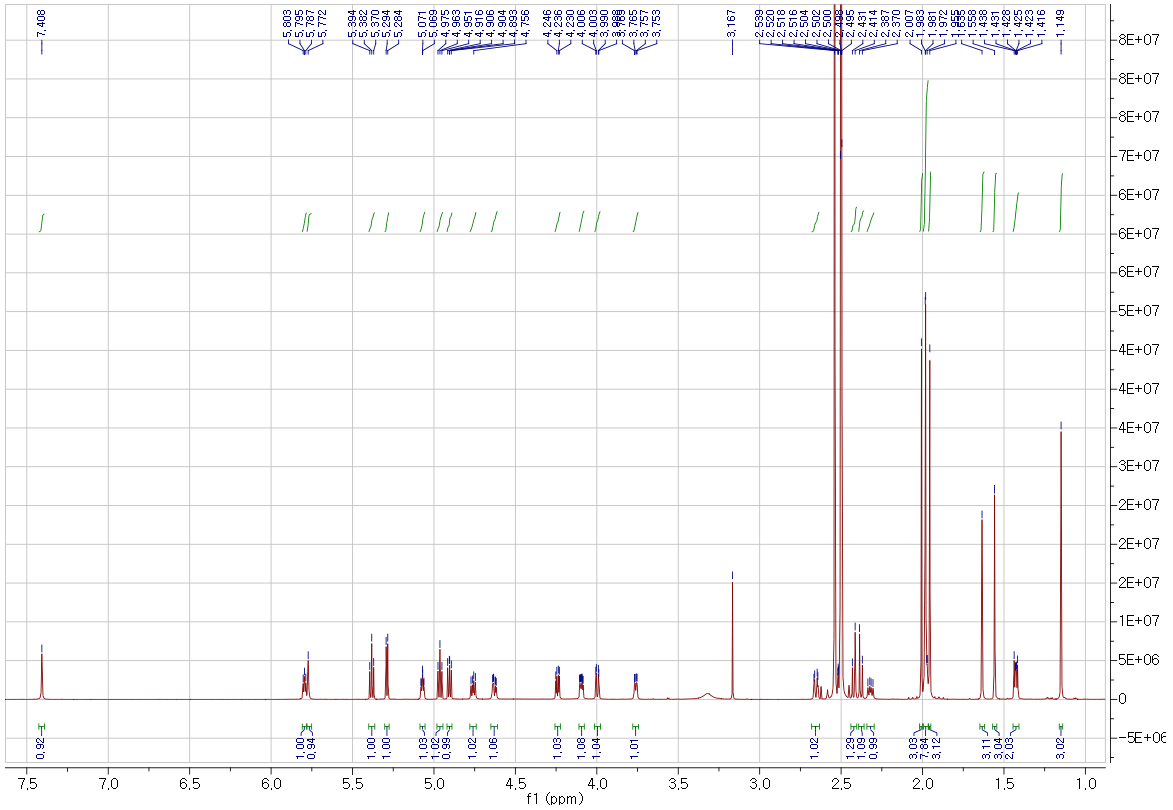
**

**
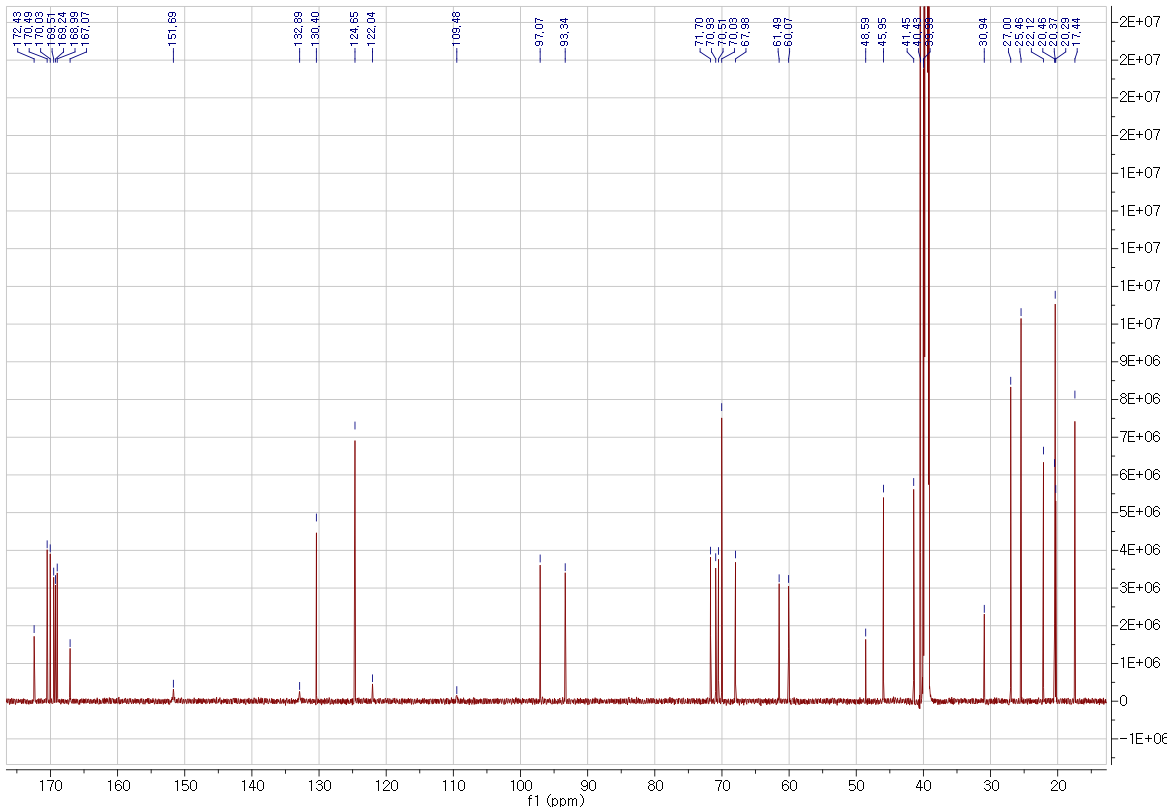
**

**Figure S46:** ^1^H and ^13^C NMR spectra of compound **10a** (800, 200 MHz, DMSO-*d*_6_)

**
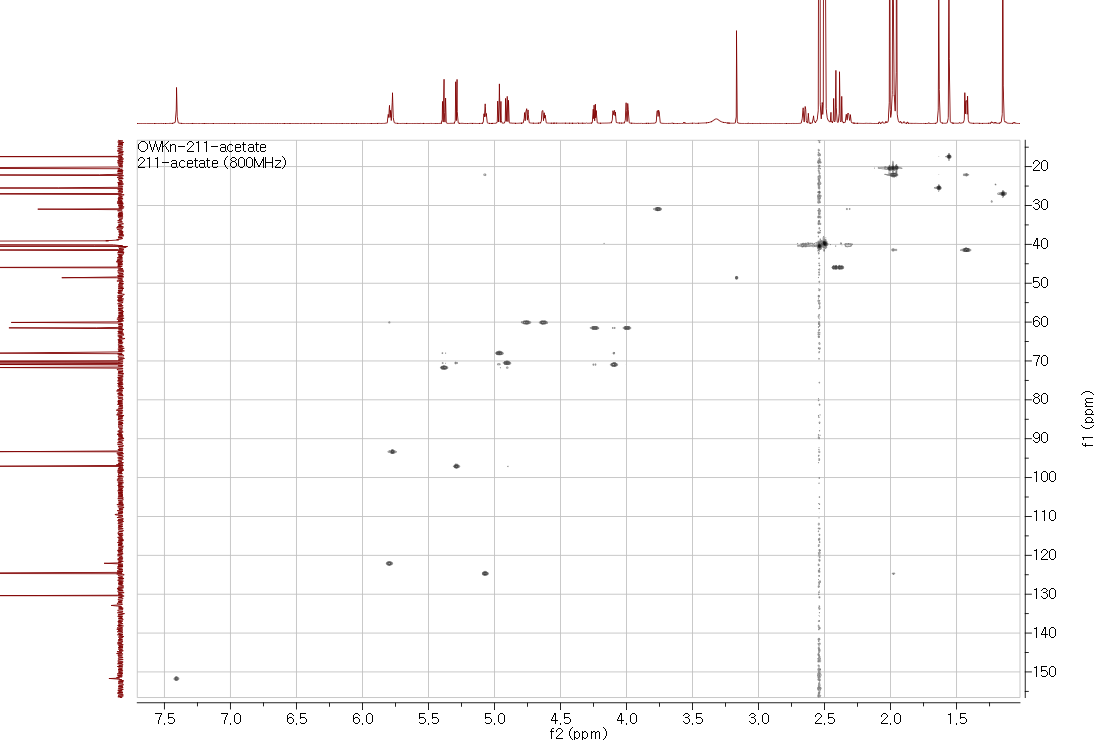
**

**
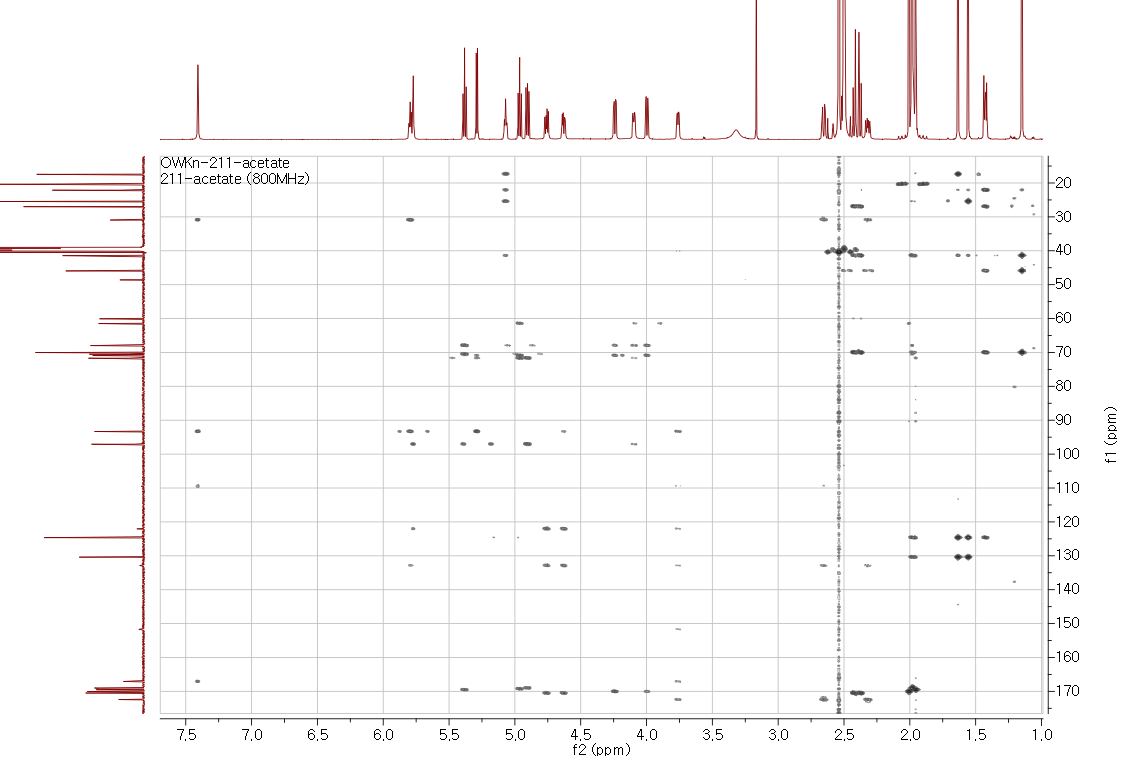
**

**Figure S47:** HSQC and HMBC NMR spectra of compound **10a** (800 MHz, DMSO-*d_6_*)

**
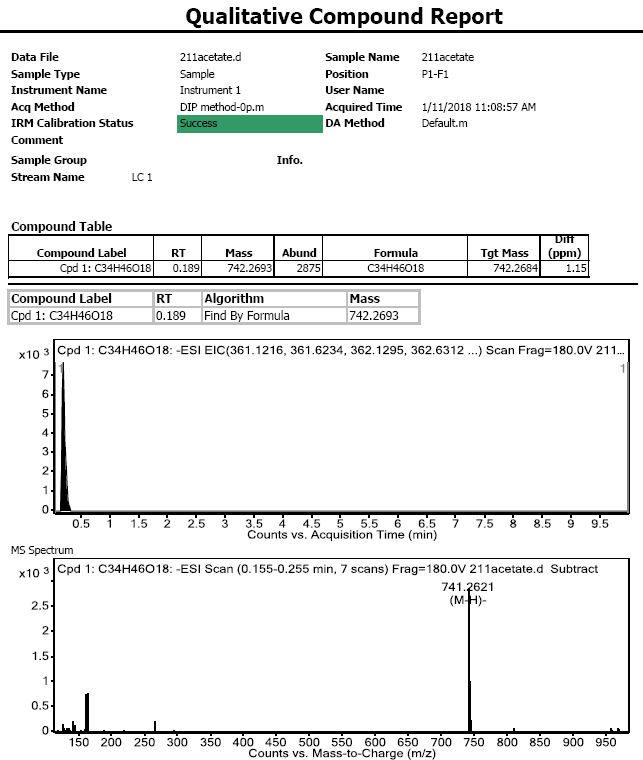
**

**Figure S48:** HR-ESI(-)MS of compound **10a**

**
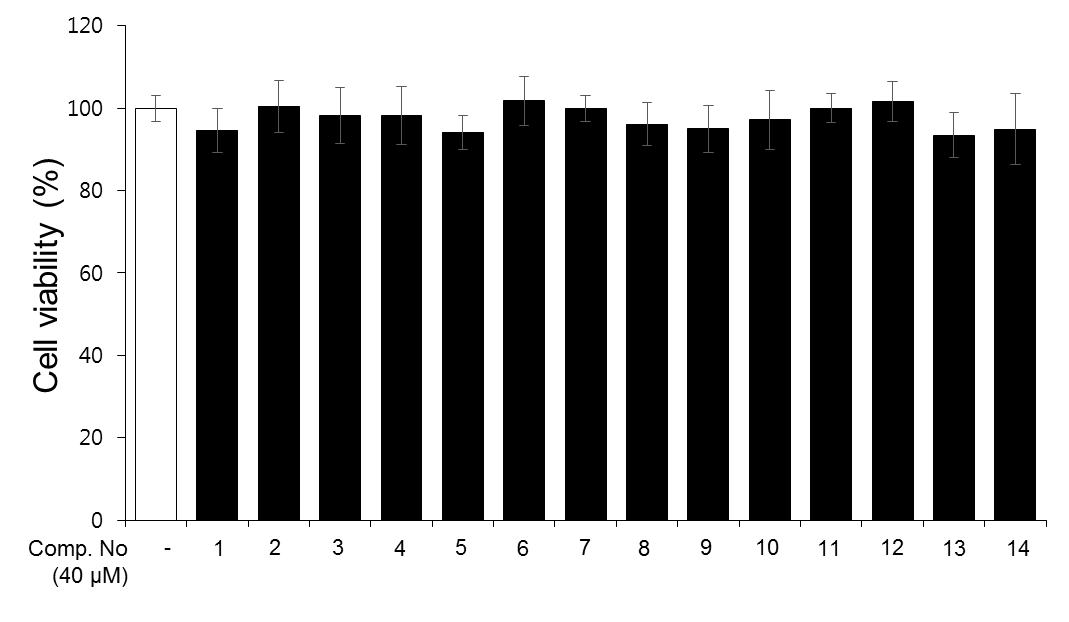
**

**Figure S49: The effect of all compounds 1-14 on cytotoxicity of 3T3-L1 adipocytes.** The cells were exposed with compounds (40 *μ*M) for 24 hours at 37 °C. The MTT assay was then performed as described in the experimental section. Data were calculated as the mean ± SD (*n* = 3), compared to the vehicle.

**
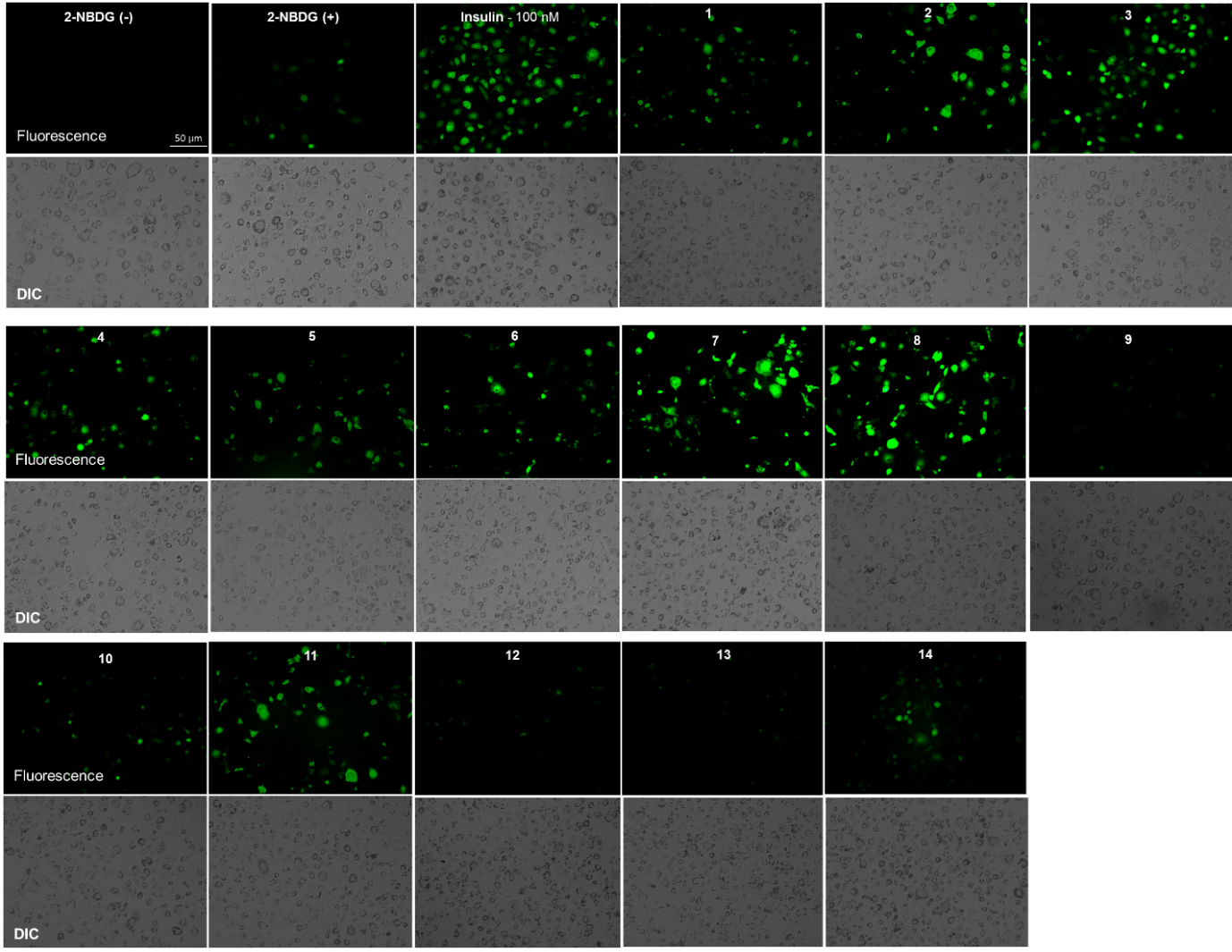
**

**Figure S50: The effects of compounds 1-14 (40 *μ*M) on 2-NBDG uptake in 3T3-L1 adipocytes.** Images were obtained by the fluorescence microscopy method.


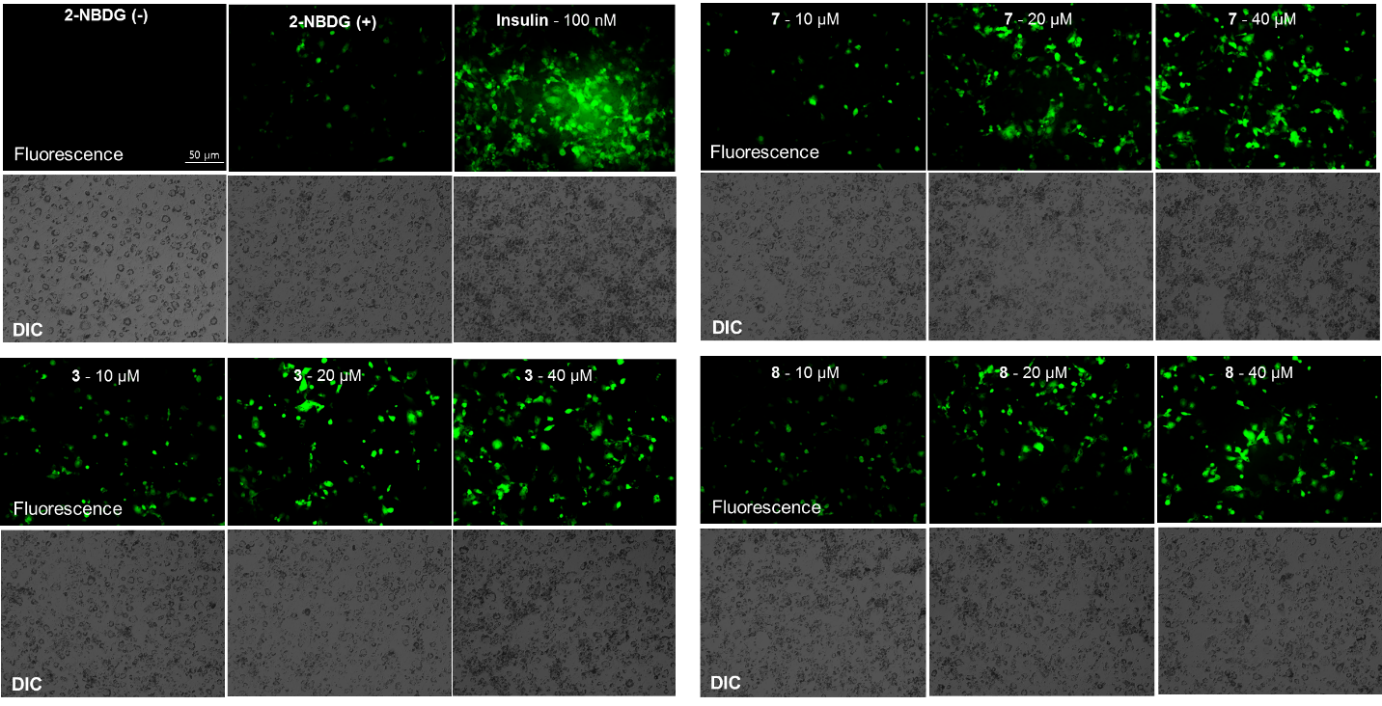


**Figure S51:** **The effects of compounds 3, 7, and 8 at different concentrations on 2-NBDG uptake in 3T3-L1 adipocytes.** Images were obtained by the fluorescence microscopy method.

**
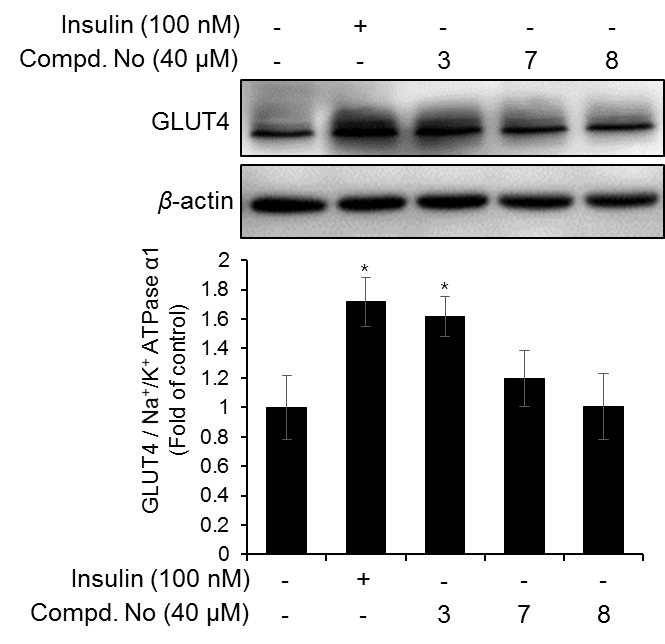
**

**Figure S52: The effects of compounds 3, 7, and 8 (40 *μ*M) on the expression of GLUT4 in whole cell lysates.** After treatment with test compounds, the cells were lysed using RIPA buffer and western blot assay was carried out as described in experimental section. The protein expression levels were normalized against *β*-actin. Results were calculated as the mean ± SD (*n*=2), ** p* < 0.05, compared to negative control. Full length western blots are provided in Supplementary Fig. S57.

**
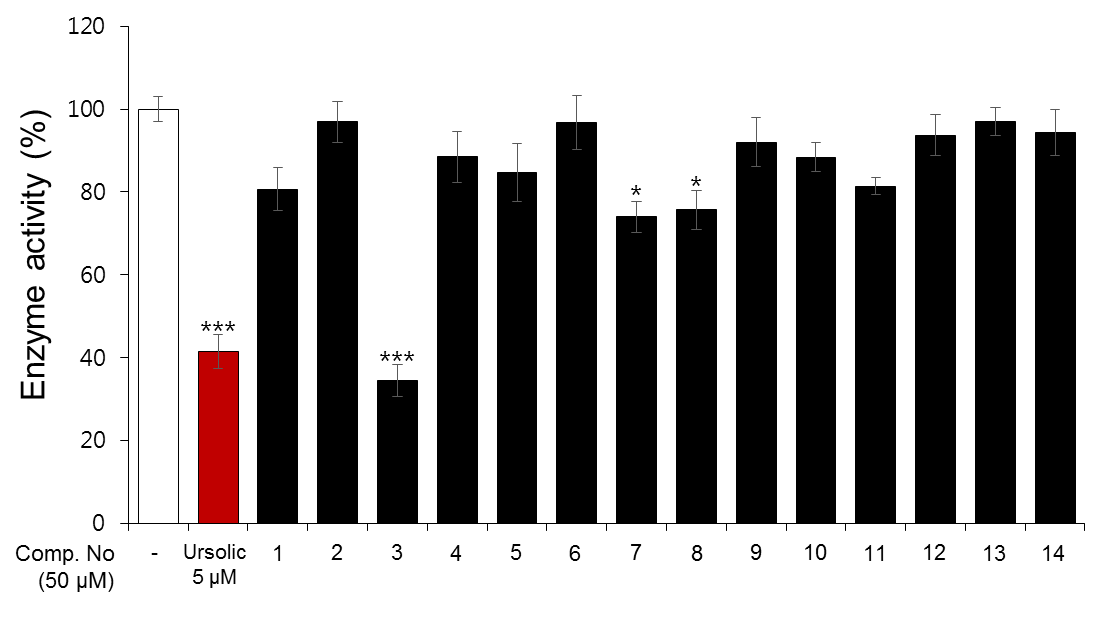
**

**Figure S53: Inhibitory effect of all compounds 1-14 on PTP1B enzyme.** Results were expressed as the mean ± SD of three independent experiments. Statistical significance was accepted at * *p* < 0.05, and *** *p* < 0.001, compared to the negative control.

**Figure S54:** Inhibitory effect of ursolic acid and compounds **3** on PTP1B enzyme with IC_50_ values of 4.58 ± 0.56 and 19.54 ± 0.67 *μ*M, respectively. Results were calculated as the mean ± SD (*n* = 3).

**
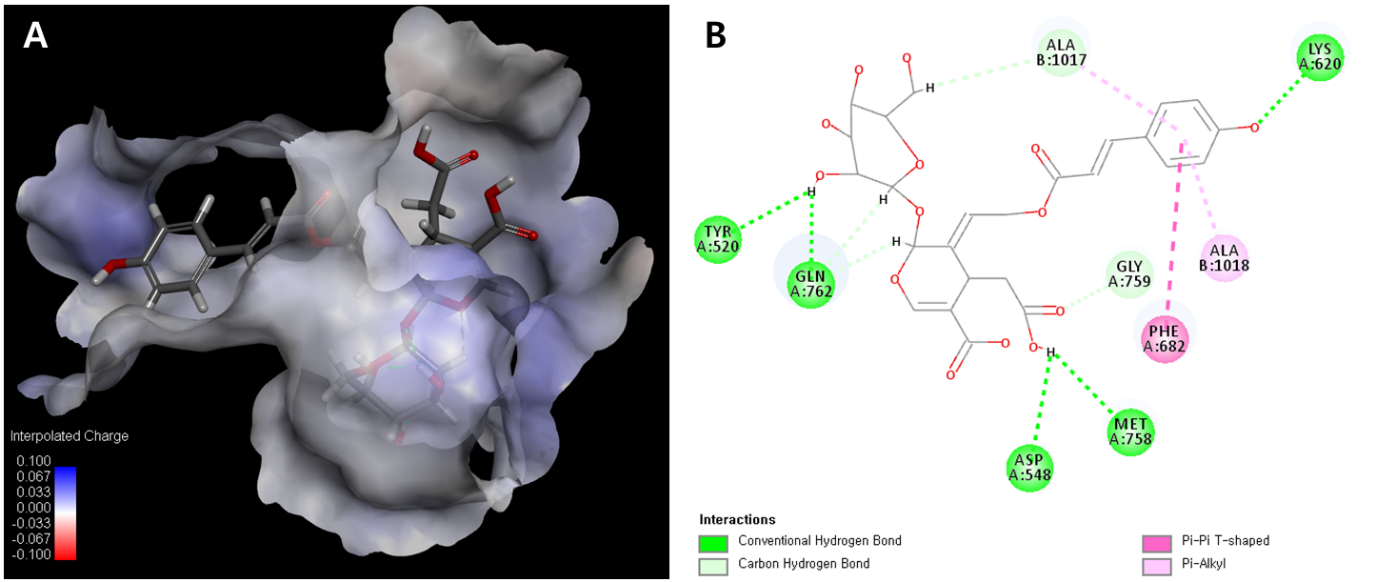
**

**Figure S55:** (**A**) 3D docking simulation of compound **3** into the active site of PTP1B (PDB code 1Q6T). (**B**) 2D diagram of the ligand interactions between compound **3** and the PTP1B enzyme.

**
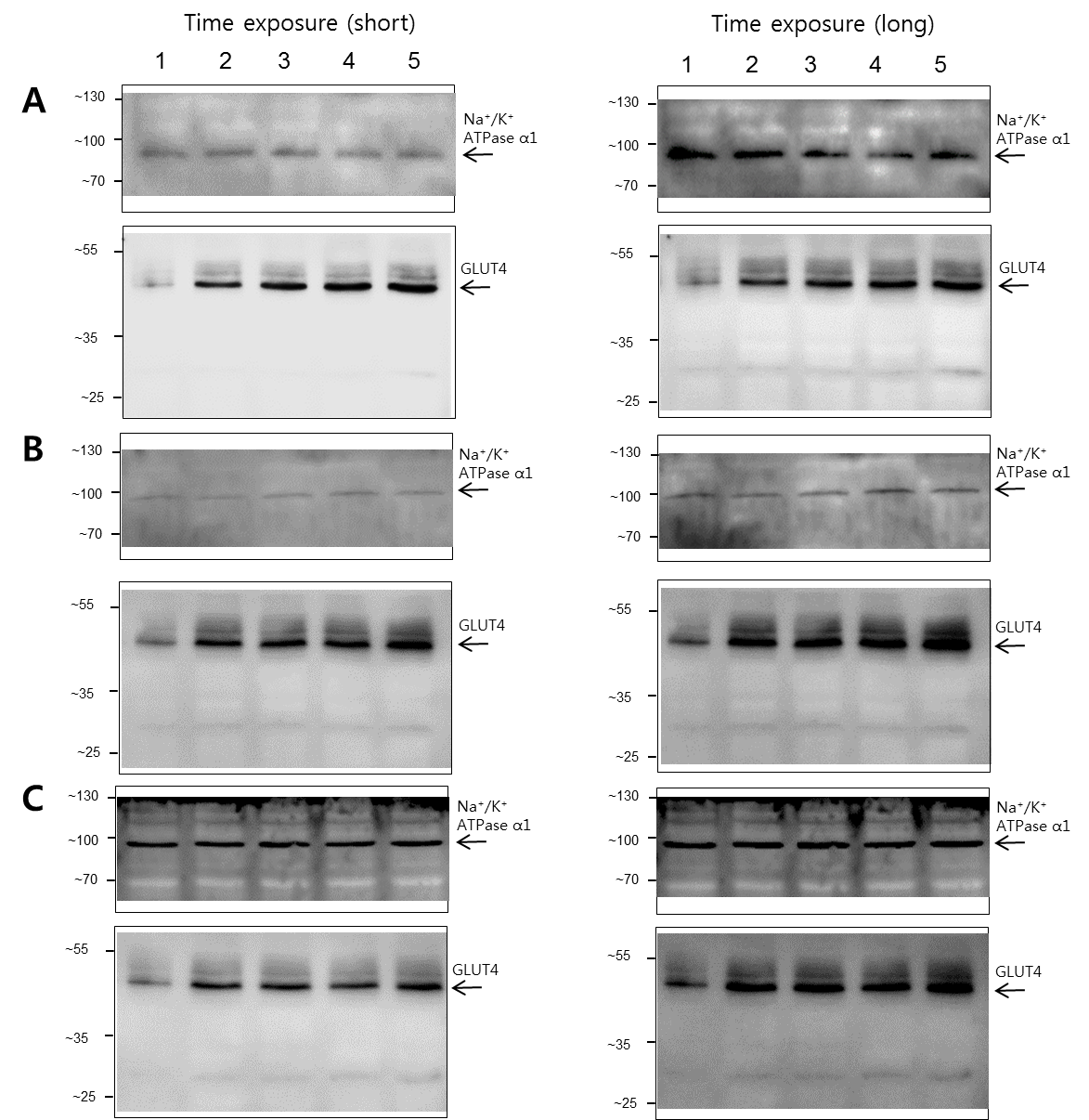
**

**
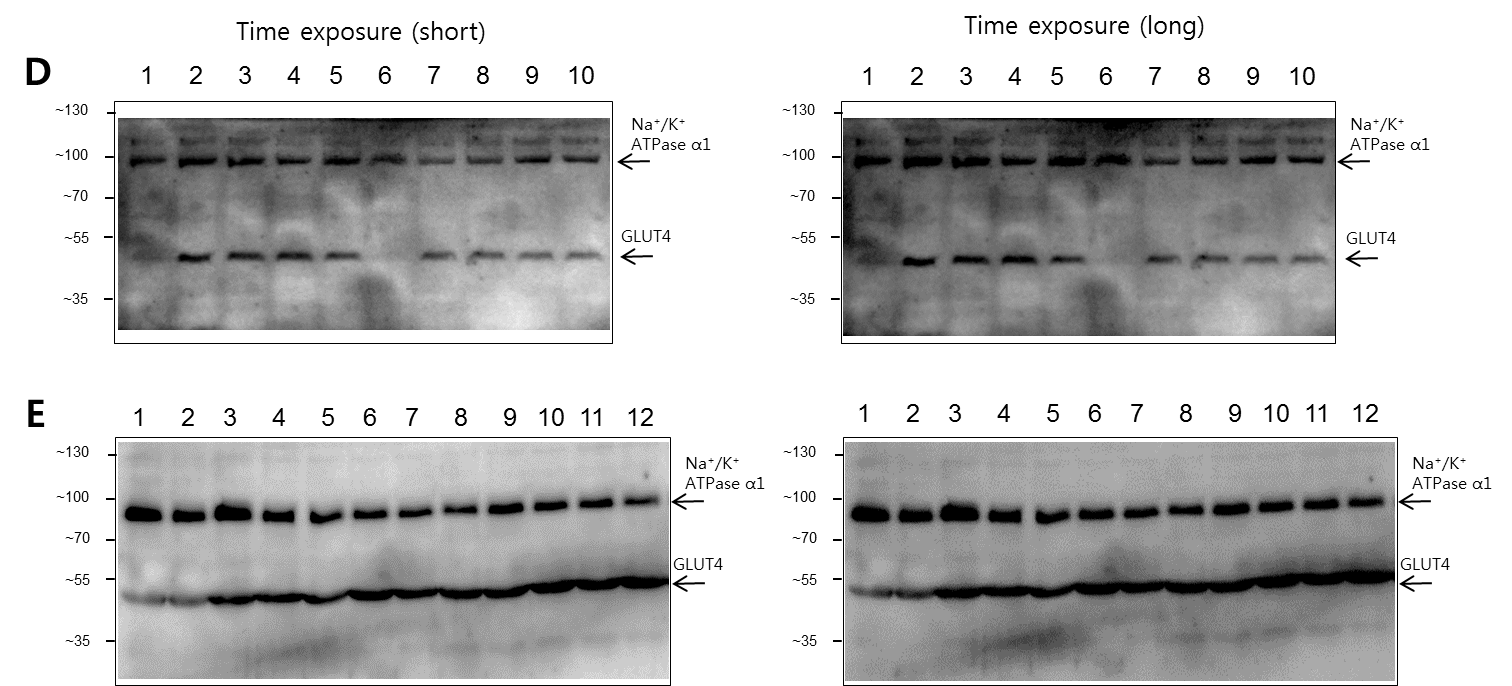
**

**Figure S56: The effects of compounds 3, 7, and 8 from *S. cochinchinensis* on GLUT4 translocation to the plasma membrane of 3T3-L1 adipocytes; original uncropped blots.** The cells were exposed with compounds (40 *μ*M) for 24 hours or insulin (100 nM) for 2 hours. After that, the plasma membrane fractions were isolated from the whole cell protein extractions as described in experiment section. Equal amounts of plasma membrane fractions were heated up to 37°C for 10 minutes and loaded on SDS-polyacrylamide gels. (A,B) The gels were transferred with condition at 30V for 3 hours. Then, membranes were cut based on protein sizes. (C) For detecting these blots, we performed experiments as described below. Equal proteins of samples (Ctrl, insulin 100 nM, compounds **3**, **7**, and **8** (40 *μ*M)) were loaded in different gels. After running gels, the gels were transferred with different conditions: 30V for 3 hours to detect GLUT4 protein; while 30V for 4 hours to detect Na^+^/K^+^ ATPase α1 protein. The cut-membranes were then incubated with GLUT4 and Na^+^/K^+^ ATPase α1 antibodies. (D,E) The transfer conditions of these blots were as follows: 30V for 4 hours and 30V for 3.5 hours, respectively. The membranes were co-incubated with GLUT4 and Na^+^/K^+^ ATPase α1 antibodies. The bands were detected using LAS 4000 luminescent image analyzer. Figure C was included in final analysis (Fig. 5). (A-C) Sample names were from 1-5 as follows: Ctrl, insulin 100 nM, compounds **3**, **7**, and **8** (40 *μ*M). (D) Sample names of experiment 4 were from 1-5 as follows: Ctrl, insulin 100 nM, compounds **3**, **7**, and **8** (40 *μ*M); while sample names of experiment 5 were from 6-10 as follows: Ctrl, insulin 100 nM, compounds **3**, **7**, and **8** (40 *μ*M). (E) Sample names of experiment 6 were from 1-12 as follows: Ctrl1, Ctrl2, insulin 100 nM, compounds **3**, **7**, and **8** (40 *μ*M)-1, compounds **3**, **7**, and **8** (40 *μ*M)-2, re-load compounds **3**, **7**, and **8** (40 *μ*M)-1.

**
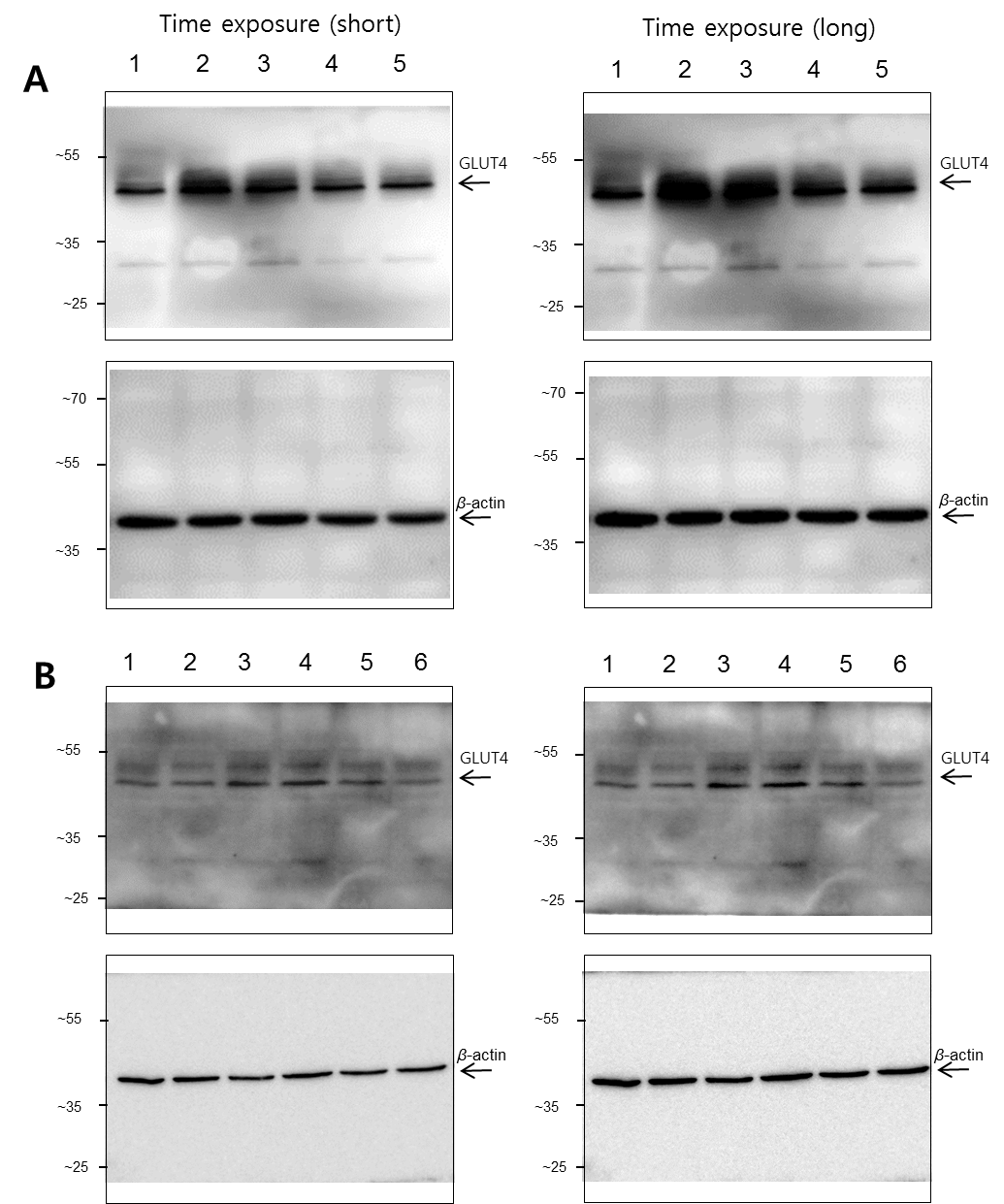
**

**Figure S57: The effects of compounds 3, 7, and 8 (40 *μ*M) on the expression of GLUT4 in whole cell lysates, original uncropped blots**. 3T3-L1 adipocytes were treated with compounds for 24 hours or insulin for 2 hours. The expressions of GLUT4 protein in whole cell lysates were evaluated by Western blotting with the transfer condition at 30V for 3 hours.. (A) For detecting GLUT4 protein, equal amounts of proteins were heated up to 37°C for 10 minutes and loaded on 12% SDS-polyacrylamide gels. For *β*-actin protein detection, however, samples were boiled at 95°C for 5 minutes, then they were separated by the 12% SDS-polyacrylamide gels. After transferred to PVDF membranes, the membranes were incubated with GLUT4 and *β*-actin antibodies. (B) Equal amounts of proteins were heated up to 50°C for 5 minutes and Western blotting was performed as above described. The expression of GLUT4 in the membrane was firstly detected, then the membrane was removed the primary and secondary antibodies using a Restore^TM^ Western blot stripping buffer (Thermo Sci.). The membrane was continually incubated with *β*-actin antibody. Figure A was included in final analysis (Fig. S91). (A) Sample names were from 1-5 as follows: Ctrl, insulin 100 nM, compounds **3**, **7**, and **8** (40 *μ*M). (B) Sample names were from 1-6 as follows: Ctrl1, Ctrl2, insulin 100 nM, compounds **3**, **7**, and **8** (40 *μ*M).

**
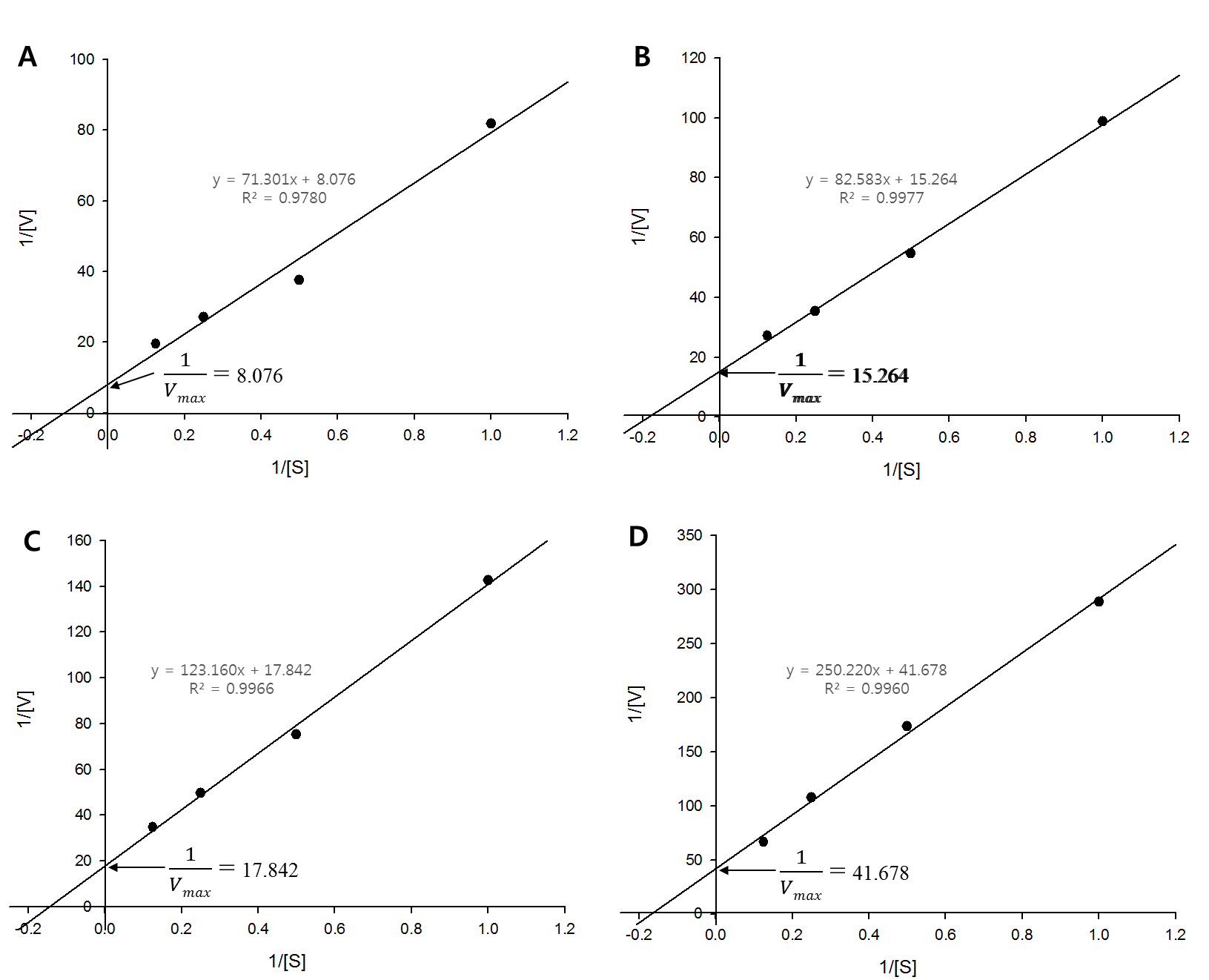
**

**Figure S58: Determination of the Ki value for noncompetitive inhibition of compound 3.** In noncompetitive inhibition type, the Ki value can be calculated using the formula:. As shown in this figure (A-D), V_max_ values were calculated at different concentrations of compound **3** (0, 10, 20, and 40 *μ*M, respectively). The Ki value was 12.46 ± 3.62 *μ*M.

$V_{\max}^{+I}= V_{\max}^{-I}/(1+\frac{[I]}{\mathrm{Ki}})$

.

**
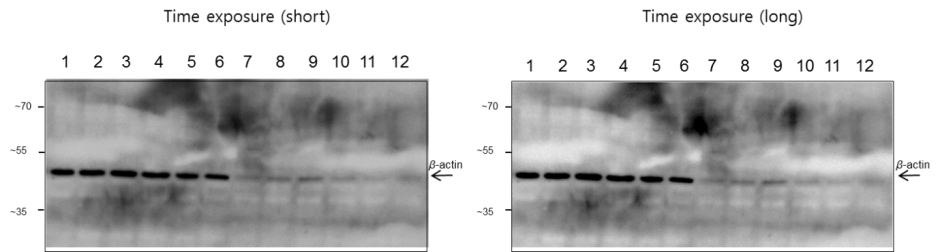
**

**Figure S59: Comparison the expression of *β*-actin protein in the whole cell lysates and the plasma membrane fractions were evaluated by Western blotting.** Equal amounts of proteins were boiled at 95°C for 5 minutes and Western blotting was performed as described in the experimental section. The transfer condition of this blot was carried out at 30V for 3 hours. Sample names of the whole cell lysates were from 1-6 as follows: Ctrl1, Ctrl2, insulin 100 nM, compounds **3**, **7**, and **8** (40 *μ*M); while sample names of the plasma membrane fractions were from 7-12 as follows: Ctrl1, Ctrl2, insulin 100 nM, compounds **3**, **7**, and **8** (40 *μ*M).


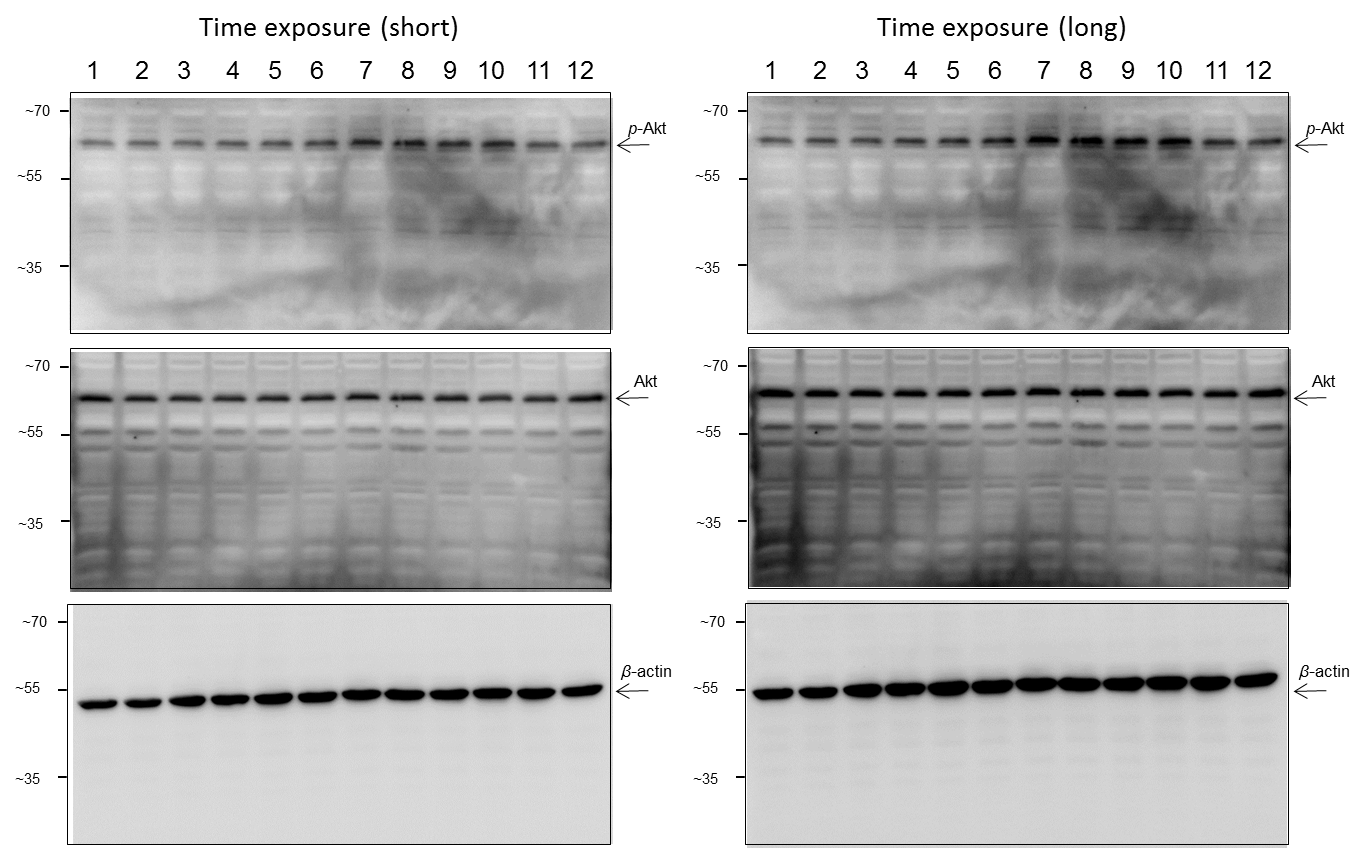


**Figure S60: The effects of incubation time on the phosphorylation of Akt by compound 3 (40 *μ*M), original uncropped blots**. 3T3-L1 adipocytes were treated with compound **3** from 1 to 24 hours of incubation. The expressions of *p*-Akt protein were evaluated by Western blotting. Equal amounts of proteins were boiled at 95°C for 5 minutes and loaded on SDS-polyacrylamide gel. After transferred to PVDF membrane, the membrane was incubated with *p*-Akt antibody. The band was detected using LAS 4000 luminescent image analyzer. Then, the membrane was removed the primary and secondary antibodies using a Restore^TM^ Western blot stripping buffer (Thermo Sci.), and incubated with Akt and *β*-actin antibodies, respectively. Sample names were from 1-12 as follows: Ctrl (1, 2, 4, 6, 12, 24 h), compound **3** (1, 2, 4, 6, 12, 24 h). Result in this figure clearly indicated that the incubation time from 1-6 hours significantly increased the expressions of *p*-Akt protein. Therefore, 2 hours of incubation was chosen for measurement of *p*-Akt protein.


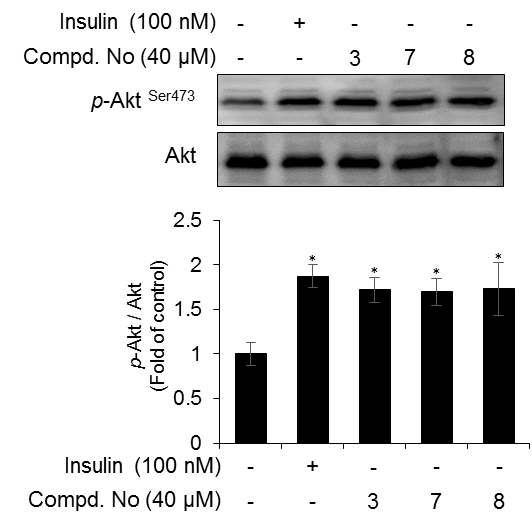


**Figure S61: The effects of compounds 3, 7, and 8 (40 *μ*M) on the phosphorylation of Akt protein.** After incubated with test compounds or insulin for 2 hours, 3T3-L1 adipocytes were lysed and Western blotting was performed as described in the experimental section. The protein expression levels were normalized against Akt protein. Data were calculated as the mean ± SD (*n*=3), ** p* < 0.05, compared to negative group.

**
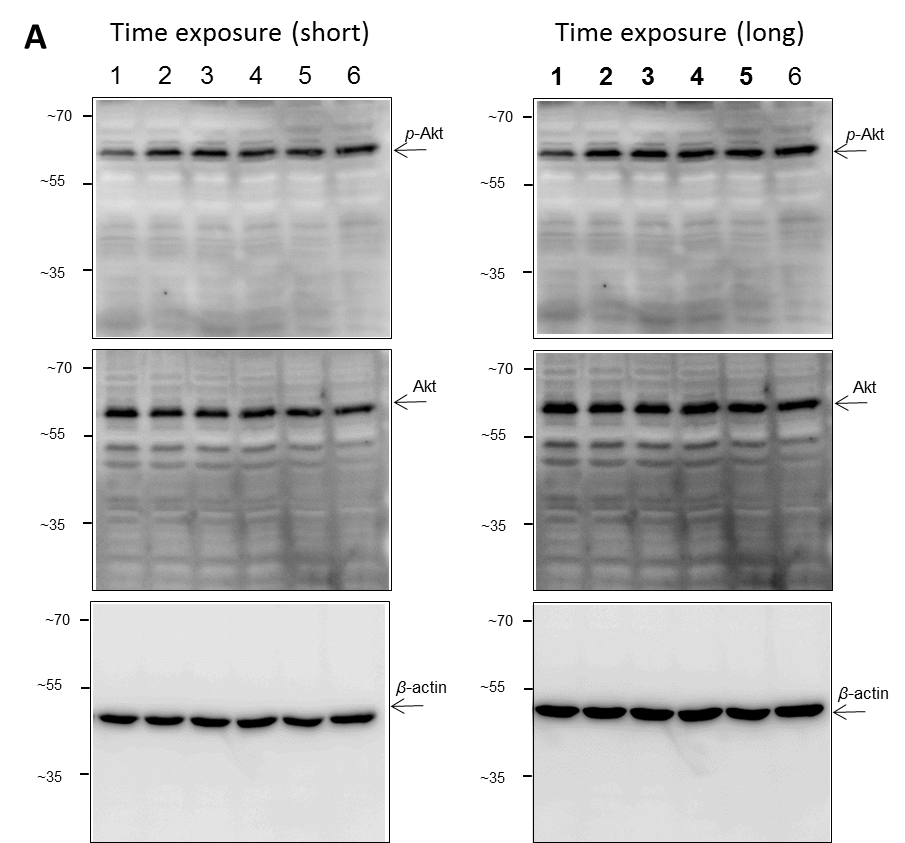
**

**
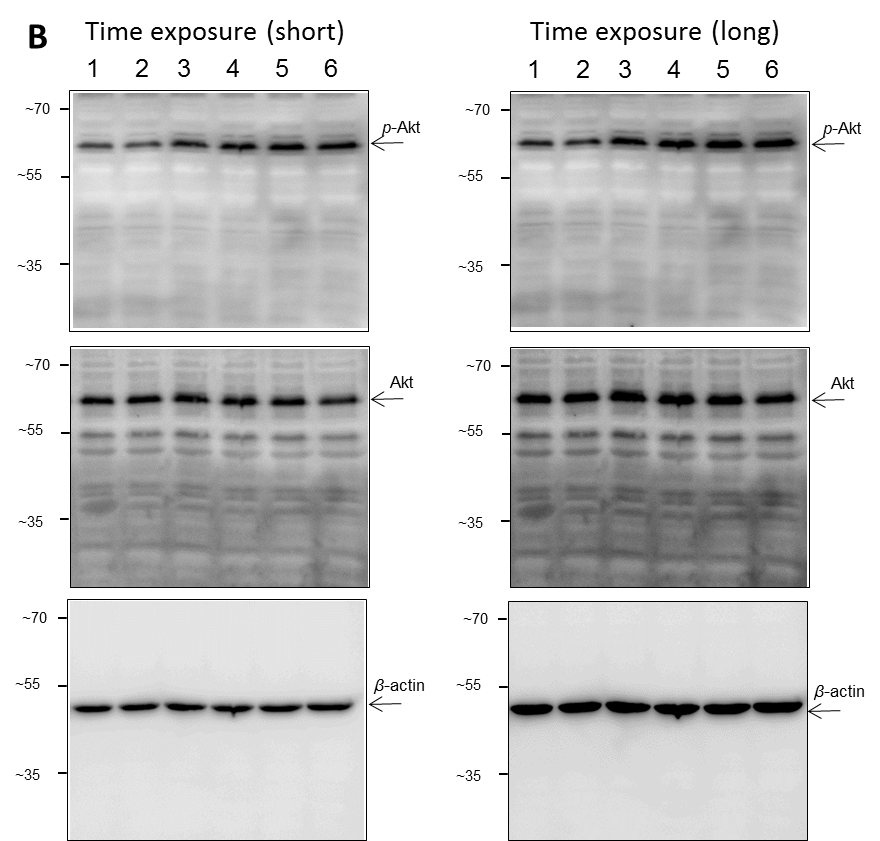
**

**
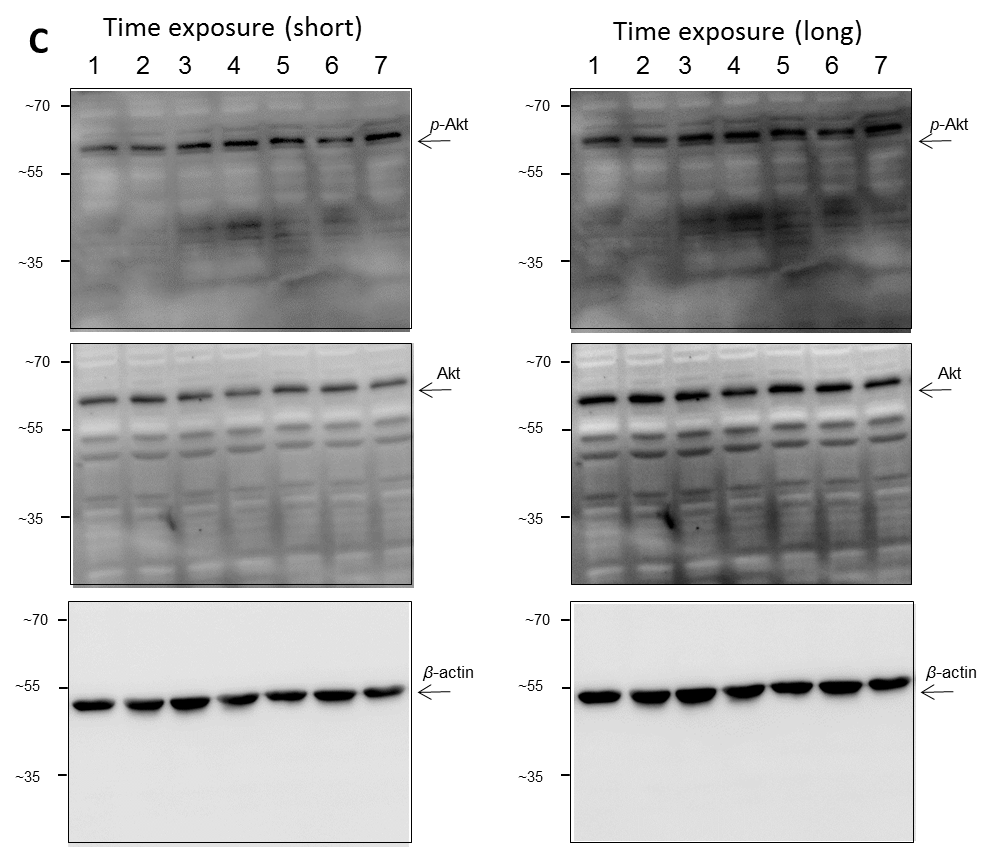
**

**Figure S62: The effects of compounds 3, 7 and 8 (40 *μ*M) on the phosphorylation of Akt in whole cell lysates (original uncropped blots)**. 3T3-L1 adipocytes were incubated with test compounds (40 *μ*M) or insulin (100 nM) for 2 hours. Equal amounts of protein were boiled at 95 °C for 5 minutes and Western blotting was performed as described in the Experimental section. Expression of *p*-AKT in whole cell lysates was first detected and primary and secondary antibodies were then removed using Restore^TM^ Western blot stripping buffer (Thermo Sci.) from the membranes. The blots were continually incubated with AKT and *β*-actin antibodies. All membranes were detected using a LAS 4000 luminescent image analyzer. Sample names were from 1-6 (A and B) and 1-7 (C) as follows: (A) Ctrl, insulin 100 nM, compounds **3**, **7** and **8** (40 *μ*M), reload insulin 100 nM; (B) Ctrl, reload Ctrl, insulin 100 nM, compounds **3**, **7** and **8** (40 *μ*M); (C) Ctrl, reload Ctrl, compounds **3**, **7**, **8** and **12** (40 *μ*M), insulin 100 nM.

**Table 1**. ^1^H and ^13^C NMR Data of compounds **10a** and **12** (^1^H 800 MHz and ^13^C 200 MHz)

|  | **10a*^a^*** | | **12*^b^*** | |
| --- | --- | --- | --- | --- |
| **position** | ***δ*_C_** | ***δ*_H_ mult. (*J* in Hz)** | ***δ_C_*** | **δ_H_ mult. (*J* in Hz)** |
| 1 | 93.3 | 5.77, s | 40.1 |  |
| 2 |  |  | 54.5 | 2.39, d (16.0) |
|  |  |  |  | 2.35, d (16.0) |
| 3 | 151.7 | 7.41, s | 201.9 |  |
| 4 | 109.5 |  | 127.1 | 5.96, s |
| 5 | 30.9 | 3.76, dd (9.3, 3.5) | 158.7 |  |
| 6 | 40.0 | 2.65, dd (15.6. 3.3) | 145.2 |  |
|  |  | 2.32, dd (15.9, 9.6) |  |  |
| 7 | 172.4 |  | 135.7 | 6.06, d (9.2) |
| 8 | 128.4 | 5.80, t (6.3) | 72.5 | 4.68, dd (9.4, 5.3) |
| 9 | 132.9 |  | 71.8 | 3.75, m |
| 10 | 60.1 | 4.76, dd (13.5, 8.0) | 18.8 | 1.21, d (6.4) |
|  |  | 4.63, m |  |  |
| 11 | 167.1 |  | 29.85 | 1.39, s |
| 12 |  |  | 29.86 | 1.34, s |
| 13 |  |  | 22.7 | 2.15, d (0.8) |
| 1’ | 170.5 |  |  |  |
| 2’ | 46.0 | 2.42, d (13.6) |  |  |
|  |  | 2.38, d (13.9) |  |  |
| 3’ | 70.0 |  |  |  |
| 4’ | 41.5 | 1.43, m |  |  |
| 5’ | 22.1 | 1.98, overlap |  |  |
| 6’ | 124.7 | 5.07, m |  |  |
| 7’ | 130.4 |  |  |  |
| 8’ | 17.4 | 1.56, br s |  |  |
| 9’ | 27.0 | 1.15, s |  |  |
| 10’ | 25.5 | 1.64, br s |  |  |
| 1’’ | 97.1 | 5.29, d (8.1) |  |  |
| 2’’ | 71.7 | 5.38, t (9.3) |  |  |
| 3’’ | 70.5 | 4.90, dd (9.9, 8.1) |  |  |
| 4’’ | 67.9 | 4.96, t (9.6) |  |  |
| 5’’ | 70.9 | 4.03, m |  |  |
| 6’’ | 61.5 | 4.24, dd (12.4, 5.0) |  |  |
|  |  | 4.00, dd (12.4, 2.1) |  |  |

*^a^* Data were measured in DMSO-*d_6_*

*^b^* Data were measured in CD_3_OD
